# PARG inhibition reduces ssDNA levels and limits RPA loading upon replication fork collapse

**DOI:** 10.1038/s44319-026-00848-8

**Published:** 2026-07-04

**Authors:** Alexandra Mihuț, Debanjan Ghosh, Adrián Kószó, Dávid Szüts, Sarka Andrs Salajkova, Martin Andrs, Jana Dobrovolna, Roberta Fajka-Boja, Gyula Timinszky

**Affiliations:** 1 Laboratory of DNA Damage and Nuclear Dynamics, Institute of Genetics, HUN-REN Biological Research Centre, H-6726 Szeged, Hungary; 2 Doctoral School of Multidisciplinary Medical Sciences, University of Szeged, H-6720 Szeged, Hungary; 3 Doctoral School of Biology, University of Szeged, H-6726 Szeged, Hungary; 4 Institute of Molecular Life Sciences, HUN-REN Research Centre for Natural Sciences, H-1117 Budapest, Hungary; 5 Institute of Molecular Cancer Research, University of Zurich, Strickhofstrasse 40a, 8057 Zurich, Switzerland; 6 Department of Genome Biology, Institute of Experimental Medicine of the Czech Academy of Sciences, Videnska 1083, 142 00 Prague 4, Czech Republic; 7 Laboratory of Cancer Cell Biology, Institute of Molecular Genetics of the Czech Academy of Sciences, Videnska 1083, 142 00 Prague 4, Czech Republic; 8 Department of Immunology, Albert Szent-Györgyi Medical School, Faculty of Science and Informatics, University of Szeged, H-6720 Szeged, Hungary

**Keywords:** DNA Replication, Recombination & Repair, Post-translational Modifications & Proteolysis, Signal Transduction

## Abstract

Poly(ADP-ribosyl)ation (PARylation) is a transient post-translational modification catalyzed by PARP enzymes and reversed by PARG. PARG inhibition causes sustained PARylation and is being explored as an anticancer strategy, but its cellular consequences remain incompletely understood. Here, we examine how persistent PARylation influences cellular responses to replication stress and DNA damage. We show that sustained PARylation reduces phosphorylated and chromatin-bound RPA most strongly under fork-stalling conditions that progress toward fork collapse. This effect requires PARP1 activity and is restrained by intact ATR–CHK1 signaling, as checkpoint inhibition renders otherwise resistant cells permissive for PARG inhibitor-associated phosphorylated RPA loss from the chromatin. The reduction of RPA phosphorylation is not dependent on BRCA1 and it is not accompanied by increased RAD51 loading. Instead, reduced chromatin-bound RPA coincides with decreased exposed ssDNA. Our results identify a checkpoint-dependent fork-collapse state in which sustained PARylation limits ssDNA and RPA levels.

## Synopsis

Sustained PARylation due to PARG inhibition decreases ssDNA levels and reduces chromatin-bound RPA. This response depends on impaired ATR–CHK1 signaling during replication stress.

- Fork collapse resulting from deficient ATR–CHK1 signaling during replication stress activates PARP1.
- PARP1 activation leads to PARylation that coincides with DNA damage markers.
- PARG inhibition shifts transient PARylation into sustained PARylation, reducing ssDNA and chromatin-bound RPA.

## Abstract (web summary)

Sustained PARylation due to PARG inhibition decreases ssDNA levels and reduces chromatin-bound RPA. This response depends on impaired ATR–CHK1 signaling during replication stress.

## Graphical abstract

**Figure:**
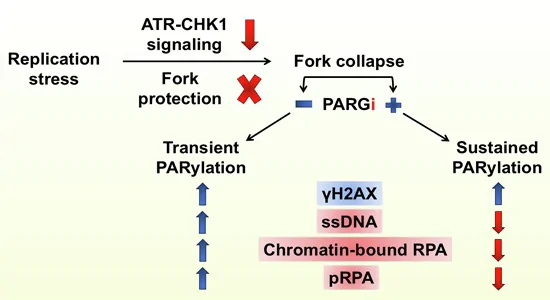

**Figure:**
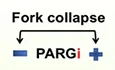

## Introduction

During DNA replication, fork progression is frequently challenged by DNA lesions, difficult-to-replicate sequences, and conflicts with transcription. Faithful genome duplication depends on the replication stress response to manage stalled replication forks and limit fork collapse (Zeman and Cimprich, 2014; Saxena and Zou, 2022). One such response is the activation of the replication checkpoint, mediated by the Ataxia telangiectasia and Rad3-related (ATR) checkpoint kinase and its effector Checkpoint kinase 1 (CHK1). The importance of this pathway is emphasized by the observation that ATR-deficient mice are embryonically lethal (Brown and Baltimore, 2000; de Klein et al, 2000). The primary activator of ATR-checkpoint signaling is the replication protein A (RPA)-coated single-stranded DNA (ssDNA), generated as a result of helicase-polymerase uncoupling (Byun et al, 2005) or DNA end resection (Saldivar et al, 2017; Zhao et al, 2020). Once activated, the ATR-checkpoint aims to stabilize the stressed forks directly by controlled remodeling and indirectly by suppressing origin firing and premature mitotic entry (Cimprich and Cortez, 2008), the failure of which results in fork collapse and marks the transition from replication stress to DNA damage (Cortez, 2015).

PARP activation triggers ADP-ribosylation (ADPr) signaling, whose main outcomes include chromatin remodeling, repair factor recruitment, protein and nucleic acid modification, ubiquitin-mediated degradation and signaling (Dasovich and Leung, 2023), thereby playing roles in DNA damage response, replication, transcriptional regulation, and cell fate decisions. ADPr is a reversible post-translational modification; the poly(ADP-ribose) (PAR) chains are rapidly removed by Poly(ADP-ribose) glycohydrolase (PARG). PARG is the principal enzyme for hydrolyzing PAR, thereby controlling the magnitude and duration of ADPr signaling (Crawford et al, 2018; Rack et al, 2021; Duma and Ahel, 2023). ADPr is increasingly recognized as an intrinsic component of S-phase DNA metabolism. Endogenous PAR in unperturbed proliferating cells is detected predominantly during S-phase at sites of DNA replication, increases strongly when the Okazaki-fragment processing enzymes LIG1 or FEN1 are perturbed, and is suppressed when Okazaki-fragment formation is blocked, implicating incompletely processed Okazaki fragments as a major endogenous source of PARP activity during DNA replication (Hanzlikova et al, 2018). In addition, at damaged or processed replication intermediates, PARP enzymes can be activated by DNA ends generated during fork collapse and DNA processing, but also by discontinuities associated with nascent-strand maturation (Rouleau-Turcotte et al, 2022; Ortega et al, 2025).

PARP1 and ADPr have been shown to regulate stalled-fork processing, such as fork remodeling and fork reversal (Yang et al, 2004; Bryant et al, 2009; Berti et al, 2013). PAR signaling has also been directly linked to checkpoint control, because PAR can promote CHK1 retention and efficient checkpoint activation at stalled replication forks. These findings suggest that ADPr can influence replication stress responses not only through the regulation of the replication fork but also through modulation of checkpoint output (Min et al, 2013).

Understanding how ADPr shapes fork fate is also clinically important. PARP inhibitors (PARPi) are already used to treat cancers with homologous recombination deficiency, while PARG inhibitors (PARGi) are in clinical trials targeting tumor cells with replication defects (Slade, 2020; Catara et al, 2026). Prior work implicated PARG in fork stability in both challenged and unchallenged cells (Illuzzi et al, 2014; Ray Chaudhuri et al, 2015). PARG depletion revealed that while PARG is dispensable for recovery from transient HU-induced fork stalling, defective PAR turnover during prolonged HU stress leads to excessive PAR accumulation associated with reduced RPA loading and hyperphosphorylation and impaired homologous recombination-associated recovery (Illuzzi et al, 2014). Later work suggested that PARG inhibition blocks fork restart, leading to replication catastrophe, providing a rationale for PARGi as a synthetic-lethal approach in cancers with replication vulnerability (Pillay et al, 2019). However, the mechanisms by which stabilized PAR chains become toxic remain poorly understood. Initial models suggested that reduced expression of replication factors sensitizes cells to PARGi-mediated replication catastrophe and subsequent cell death (Pillay et al, 2019; Coulson-Gilmer et al, 2021). More recent work, however, did not consistently support low expression of replication genes as an explanation for PARGi-induced replication catastrophe (Coulson-Gilmer et al, 2021). Moreover, not all instances of PARGi-mediated cytotoxicity were accompanied by canonical markers of replication catastrophe (Coulson-Gilmer et al, 2021).

Conflicting findings have also been reported regarding RPA regulation upon PARGi, a key mediator of replication stress response. In some contexts, PARG defect reduces RPA phosphorylation and chromatin-loading, leaving ssDNA unprotected (Illuzzi et al, 2014), whereas in others, PARGi increases RPA foci, marking impending replication catastrophe (Pillay et al, 2019; Coulson-Gilmer et al, 2021). Although RPA-coated ssDNA is central to fork protection and replication checkpoint activation, persistent RPA accumulation on DNA under prolonged stress reflects a shift from controlled fork stalling toward DNA damage-associated fork collapse, where PARP1-dependent signaling becomes prominent. Clarifying under which conditions, and how, sustained ADPr affects DNA-bound RPA is therefore essential to understand its potential clinical relevance. Accordingly, in this study, we examine the relationship between sustained poly(ADP-ribosyl)ation (PARylation) and RPA regulation at stressed replication forks using a range of replication stressors and cell line models.

## Results

### Sustained PARylation interferes with the replication stress response

It has been shown that PARP1 binds to and is activated at stalled replication forks, and that its activity is required for efficient recovery of replication after fork stalling (Bryant et al, 2009). However, the PAR chains generated at stressed forks are short-lived and are difficult to detect unless PARG is inhibited or depleted (Hanzlikova et al, 2018; Illuzzi et al, 2014). To examine the accumulation of PARylation upon replication stress, we added PARGi to P19 mouse teratocarcinoma cells treated with hydroxyurea (HU) or camptothecin (CPT) for various time periods. The P19 cell line was chosen because it is highly proliferative, with more than 50% of the cells in S-phase in unsynchronized populations. HU blocks DNA synthesis by inhibiting the ribonucleotide reductase enzyme and thereby decreasing the dNTP pool (Musiałek and Rybaczek, 2021) and potentially also by inhibiting DNA polymerases through oxidation (Shaw et al, 2024) while CPT binds to the Topoisomerase I–DNA complex, trapping the enzyme on the DNA and obstructing fork progression, and creating double-strand DNA breaks (DSBs) when DNA replication encounters Topoisomerase I-caused ssDNA breaks (Li et al, 2017).

**Figure 1:**
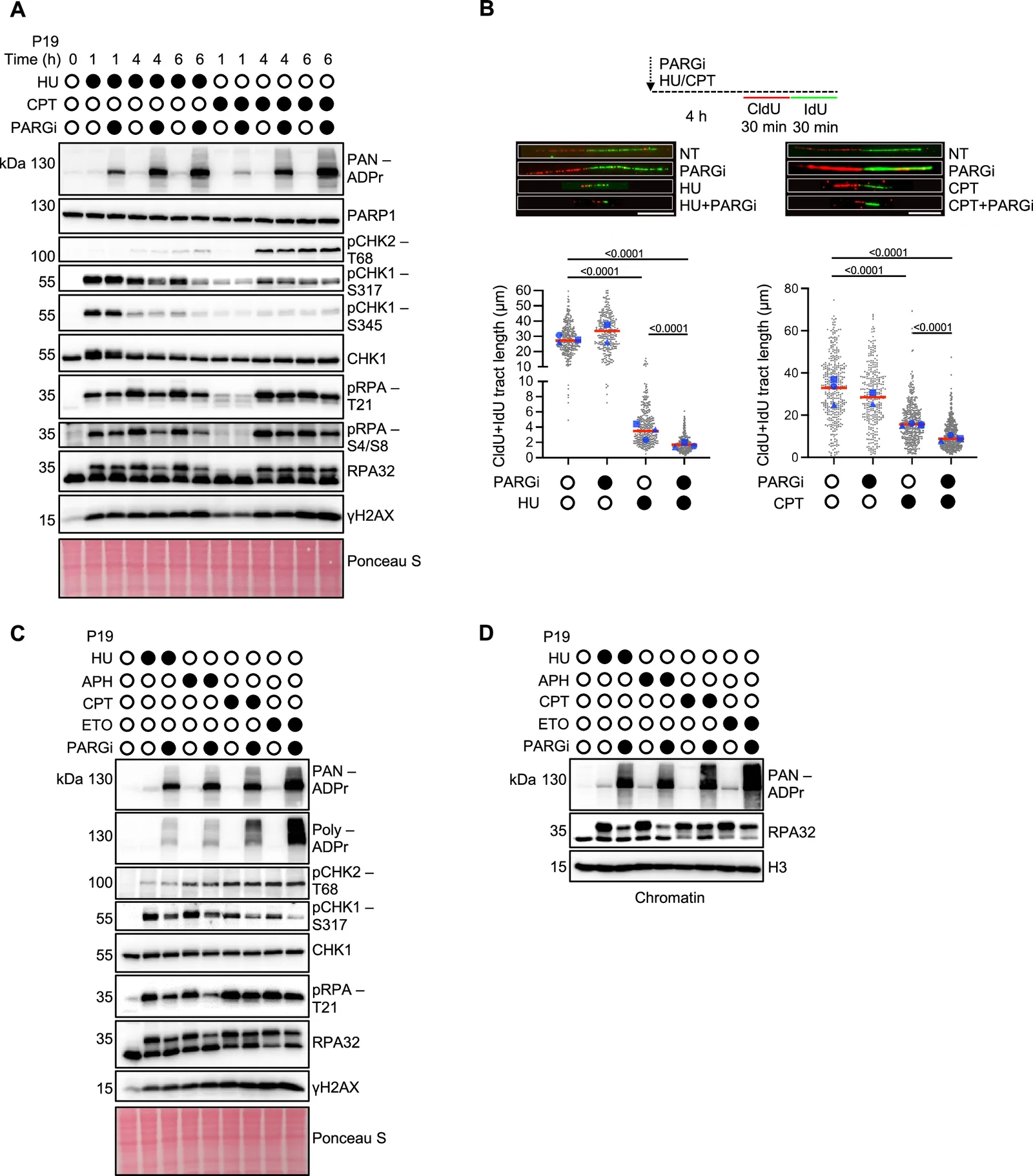
Replication stress response in the presence of persistent PARylation. (**A**) P19 cells were treated with HU (0.5 mM) or CPT (5 µM), with or without PARGi (5 µM), for the indicated time points, lysed and analyzed by western blotting with the indicated antibodies. Representative blot from three biological replicates. Samples from the same experiment were run on parallel gels. The corresponding intra-gel Ponceau S staining used as a loading control for each blot are provided in the Source Data file. (**B**) Above: Scheme of DNA fiber assay labeling of P19 cells treated with HU (0.5 mM), CPT (5 µM), and PARGi (5 µM). Representative images of DNA replication tracts are shown. Scale bar: 10 µm. Below: Super plot of DNA replication velocity in P19 cells. The scatter plot shows the lengths of individual DNA tracts (*n* > 300; small dots) and the medians of two (for PARGi treatment) and three (non-treated, HU, HU+PARGi, CPT, or CPT + PARGi treated) biological replicates, represented by different blue shapes. Black lines indicate medians of all replication tracts. The nonparametric Kruskal–Wallis test from individual DNA tracts was used to determine significance, *P* < 0.0001 (****). (**C**) P19 cells were treated with HU (0.5 mM), APH (10 µM), CPT (5 µM), and ETO (10 µM) with or without PARGi (5 µM) for 4 h. Cells were then lysed and subjected to western blotting with the indicated antibodies. Samples from the same experiment were run on parallel gels. The corresponding intra-gel Ponceau S staining used as a loading control for each blot are provided in the Source Data file. Representative blot from 3 biological replicates. (**D**) PARylation and RPA32 levels on the chromatin of P19 cells treated with HU (0.5 mM), APH (10 µM), CPT (5 µM), and ETO (10 µM) with or without PARGi (5 µM) for 4 h were assessed by western blotting. H3 staining was used as a loading control. Representative blot from three biological replicates. Source data are available online for this figure.

As expected, both HU and CPT led to an increase in PARylation over time that was readily detectable only in the presence of PARGi (Fig. 1A). However, the associated signaling responses differed markedly between the two treatments. CPT treatment elicited only a modest stress response at 1 h, with low levels of CHK1, RPA32, and H2AX phosphorylation. These signals increased over time and were accompanied by CHK2 phosphorylation and high levels of γH2AX at 6 h, consistent with ataxia-telangiectasia mutated (ATM) activation and prominent DNA damage response, possibly caused by DSBs (Zannini et al, 2014) (Fig. 1A). In contrast, HU treatment induced excessive amount of phospho-CHK1 both at S317 and S345 sites, known to be primarily phosphorylated by ATR (Zhao and Piwnica-Worms, 2001), already at 1 h of treatment, the shortest treatment time we examined, and decreased over time. The γH2AX was high throughout the time course, whereas CHK2 phosphorylation was barely detectable. Despite the decreasing pCHK1 levels, the hyperphosphorylated forms of RPA32 at T21 and S4/S8 sites—which indicate severe replication stress and DNA damage (Liu et al, 2012)— increased over time during HU treatment, unless combined with PARGi. PARGi co-treatment resulted in the marked reduction in the level of phosphorylated RPA32 forms (Fig. 1A) in the case of HU; however, it was less prominent upon CPT treatment at the examined time points. Importantly, we found that combining PARGi with HU reduced CHK1 phosphorylation, while leaving pCHK2 and γH2AX levels unaltered, suggesting that sustained PARylation interferes with replication stress signaling rather than suppressing the overall DNA damage response (Fig. 1A).

**Figure EV1:**
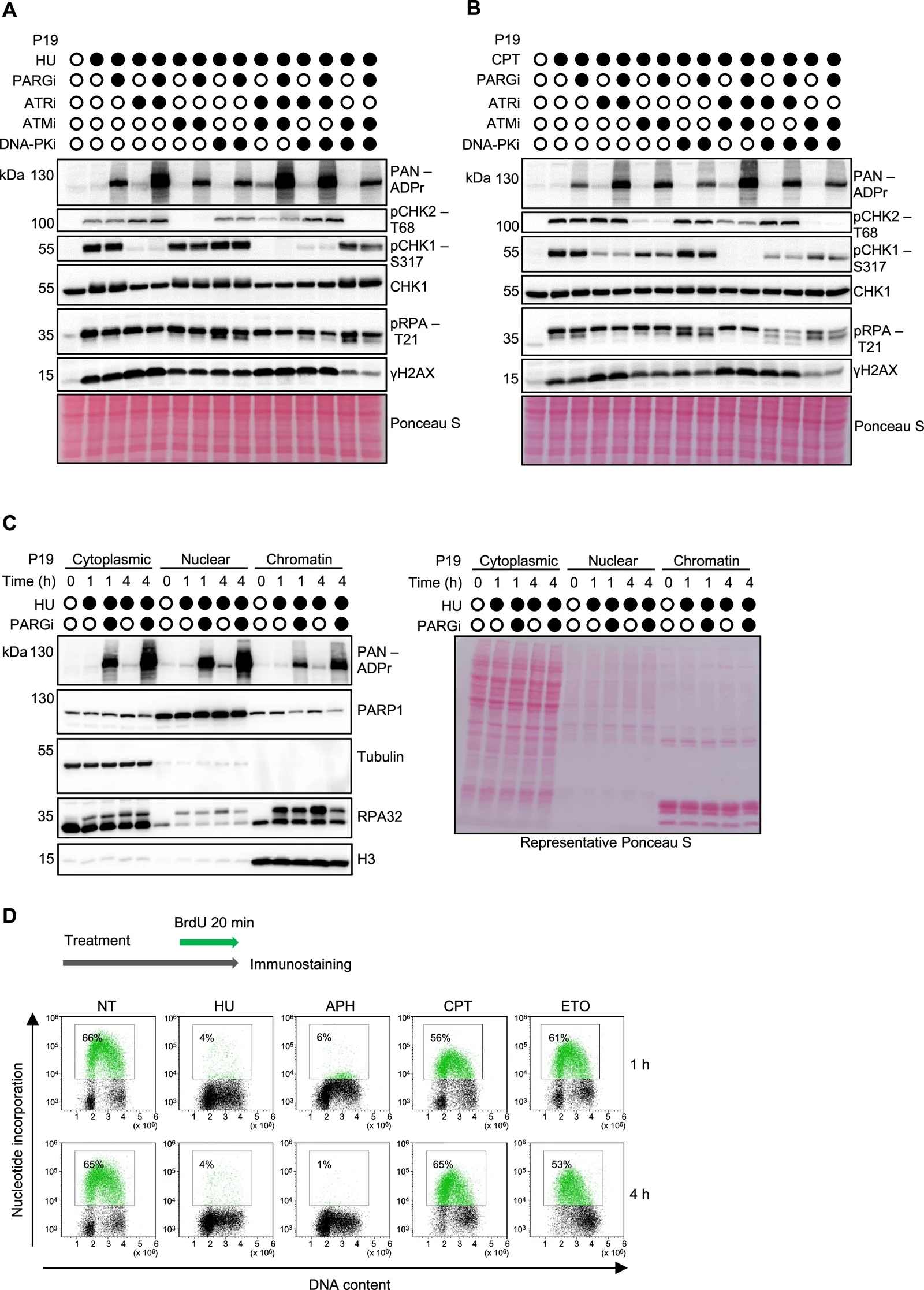
Differences in HU and CPT-induced early replication stress signaling. (**A**, **B**) P19 cells were pre-treated with ATRi (5 µM), ATMi (5 µM), and DNA-PKi (5 µM) for 30 min after which HU (0.5 mM) (**A**) or CPT (5 µM) (**B**) were added for an additional 1 h and 30 min with or without PARGi (5 µM). Cells were then lysed and subjected to western blotting with the indicated antibodies. Representative blot from three biological replicates. Samples from the same experiment were run on parallel gels. The corresponding intra-gel Ponceau S staining used as a loading control for each blot are provided in the Source Data file. (**C**) P19 cells were treated with HU (0.5 mM) with or without PARGi (5 µM) for the indicated time points and subjected to cell fractionation to assess the levels of PARylation and RPA32 in cytoplasmic, nuclear or chromatin fraction by western blotting. Ponceau S staining was used as a loading control. Representative blot from three biological replicates. (**D**) P19 cells were treated with HU (0.5 mM), APH (10 µM), CPT (5 µM), and ETO (10 µM) for 1 h. BrdU was added for the last 20 min to show the nucleotide incorporation. The cells were then fixed and stained with anti-BrdU and propidium iodide (DNA content), and then the samples were analyzed with flow cytometry. FACS images of a representative from three biological replicates are shown. Source data are available online for this figure.

To further analyze the effect of PARG inhibition on HU- and CPT-induced replication stress, we assessed the early stress response as controlled by the three main replication stress and DNA damage kinases, ATR, ATM and DNA-dependent protein kinase (DNA-PK). These kinases were inhibited for 30 min prior to the HU and CPT treatments, which were then added to the cells for an additional 1 h and 30 min together with the kinase inhibitors. At this short incubation time, both HU and CPT induced RPA32 and H2AX phosphorylation, but the reduction in pRPA in the presence of PARGi was still modest (Fig. EV1A,B). However, the difference between HU and CPT checkpoint signaling was already evident: HU primarily triggered CHK1 phosphorylation controlled by ATR and, to a lesser extent, by ATM activity, whereas CPT elicited a stronger CHK2 response consistent with ATM and DNA-PK activation at DNA breaks, in line with the literature data (Zhao and Piwnica-Worms, 2001; Pommier, 2006). The dominance of ATR control upon HU-treatment was also indicated by elevated PARylation and γH2AX levels when ATR was inhibited, presumably due to consequential fork collapse (Fig. EV1A).

While severe replication stress leads to a prominent pRPA-T21 signal, none of the tested PIKK inhibitors abolished this signal, suggesting that T21 phosphorylation is not controlled solely by any of these kinases under the tested conditions (Fig. EV1A,B). The observed PARGi-induced reduction in pRPA-T21 is unlikely to be explained simply by reduced activity of ATR, ATM, or DNA-PK, because the decrease persisted even when any two of the three kinases were inhibited. Instead, it more likely reflects reduced chromatin-associated RPA. During replication stress, pRPA largely reflects chromatin-associated, ssDNA-bound RPA at stalled or processed replication forks (Olson et al, 2006), so changes in RPA loading onto chromatin are also expected to influence the pRPA signal. Therefore, we asked whether sustained PARylation affects the amount of chromatin-bound RPA32. P19 cells were treated with HU for 1 or 4 h in the presence or absence of PARGi, and cytoplasmic, nuclear, and chromatin fractions were analyzed for RPA32 (Fig. EV1C). The faster-migrating band of RPA32, which represents the non-phosphorylated form, was found mostly in the cytoplasmic fraction under all conditions, while the slower migrating phosphorylated band was associated with the chromatin fraction in HU-treated samples, even at shorter incubation time. In the presence of PARGi, the amount of pRPA was reduced and inversely correlated with the PARylation signal (Fig. EV1C). It should be noted that the PARGi-mediated reduction in HU-induced pRPA was not accompanied by a corresponding increase in the faster-migrating non-phosphorylated form, suggesting that sustained PARylation reduces the overall amount of DNA-bound RPA32 rather than merely altering its phosphorylation state.

Together, these results suggest that sustained PARylation alters checkpoint signaling in a stress-dependent manner. The effect is more pronounced under HU treatment, where PARG inhibition strongly reduces CHK1 and RPA32 phosphorylation, suggesting disruption of the canonical response to replication fork stalling. In addition, the reduction in phosphorylated RPA32 by PARGi is unlikely to reflect direct kinase inhibition and instead points to decreased chromatin-associated RPA.

### The effect of sustained PARylation is stronger under fork-stalling conditions and weaker in topoisomerase poison-induced responses

To better understand replication dynamics, we performed a DNA fiber spreading assay in P19 cells. This approach enables single-molecule tracking of DNA replication forks by sequentially incorporating nucleoside analogs, 5-chloro-2′-deoxyuridine (CldU) and 5-iodo-2′-deoxyuridine (IdU), into newly synthesized DNA and stretching DNA on microscope slides. By measuring the CldU and IdU tract lengths, we can observe how DNA replication progresses (Quinet et al, 2017). Whereas HU almost completely suppressed CldU and IdU incorporation, indicating near-complete DNA replication arrest (Fig. 1B, left panel), CPT induced replication stress and a slowdown, as the lengths of CldU and IdU were shortened by ~50% (Fig. 1B, right panel). In the presence of HU and CPT, inhibition of PARG further shortened replication tracts, suggesting increased fork stalling and/or defective recovery from stress-induced fork remodeling. Thus, sustained PARylation not only alters checkpoint signaling but also further impairs replication fork progression under stress. Together with the signaling data, this supports a model in which persistent PAR compromises efficient fork regulation during replication stress.

**Figure EV2:**
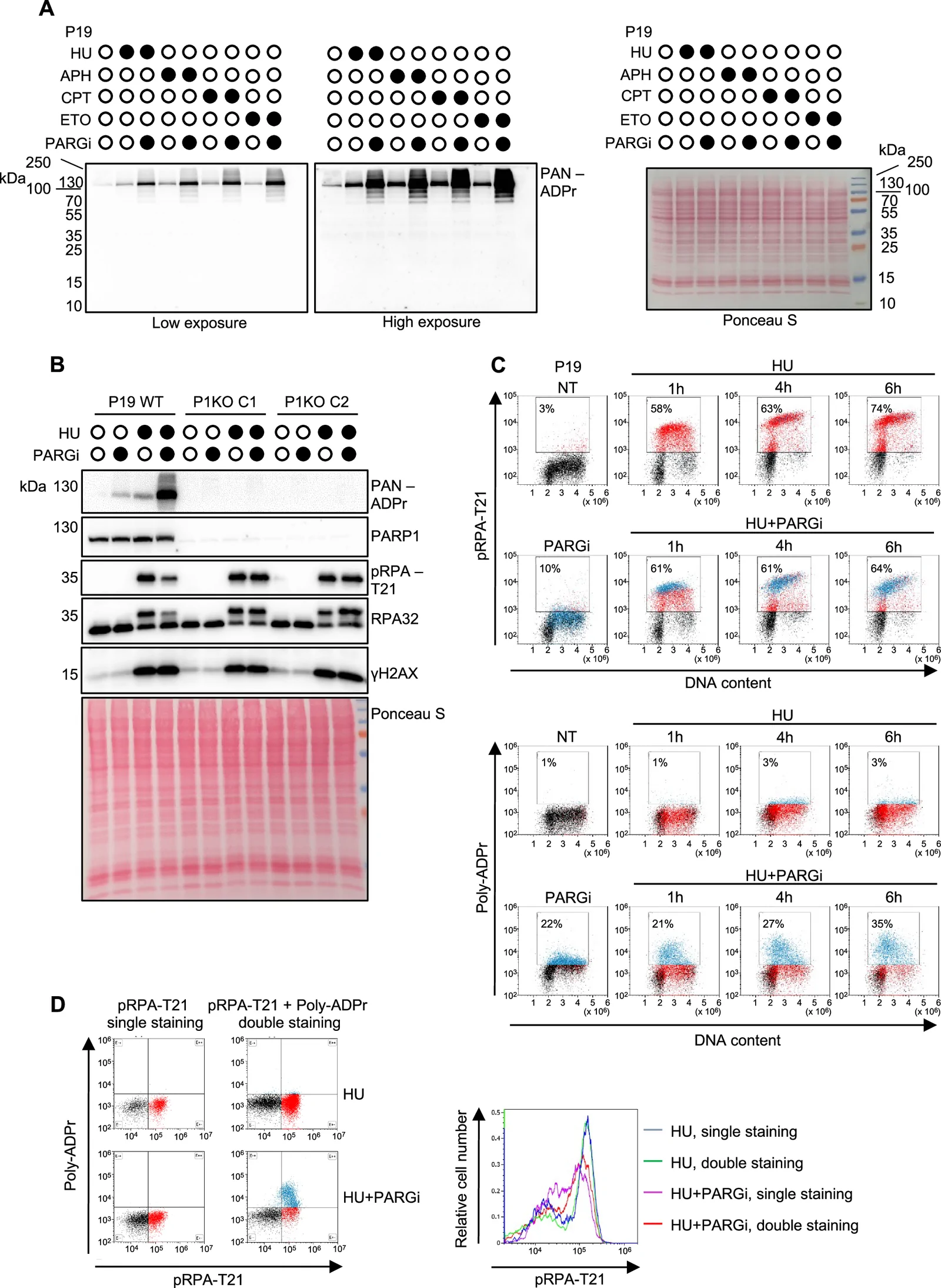
Role of PARylation in pRPA reduction. (**A**) P19 cells were treated with HU (0.5 mM), APH (10 µM), CPT (5 µM), and ETO (10 µM) with or without PARGi (5 µM) for 4 h. Cells were then lysed and subjected to western blotting with the PAN-ADPr detecting reagent (MABE1016). Ponceau S staining was used as a loading control. Representative blot from 2 biological replicates. (**B**) P19 WT and PARP1 KO cells (clones P1KO C1 and P1KO C2) were treated with PARGi (5 µM), HU (0.5 mM) and their combination for 7 h, then lysed, and samples were analyzed by western blotting with the indicated antibodies. Representative blot from three biological replicates. Samples from the same experiment were run on parallel gels. The corresponding intra-gel Ponceau S staining used as a loading control for each blot are provided in the Source Data file. (**C**) P19 cells were treated with HU (0.5 mM) with or without PARGi (5 µM) for the indicated time points, fixed and analyzed by FACS with the indicated antibodies. The pRPA-T21^+^ cells are colored in red, and PAR^+^ cells are blue throughout all of the FACS images. The images are from the same representative experiment shown in Fig. 1D, E. (**D**) P19 cells were treated with HU (0.5 mM) with or without PARGi (5 µM) for 4 h, then fixed, stained with either pRPA-T21 antibody alone or in combination with Poly-ADPr antibody, and then analyzed by FACS. Representative FACS images from 3 biological replicates are shown. Left: Dot plots of pRPA-T21 and Poly-ADPr fluorescence intensity show the inverse correlation of the signals. Right: Overlay histograms show that Poly-ADPr antibody does not interfere with the pRPA-T21 signal. Source data are available online for this figure.

To determine whether the effects of PARGi extend beyond replication stress resulting from HU-induced dNTP depletion and CPT-induced DNA lesions, we expanded the analysis to aphidicolin (APH) and etoposide (ETO). APH induces replication fork stalling by inhibiting replicative DNA polymerases (Cheng and Kuchta, 1993), whereas ETO causes topoisomerase II-dependent DNA lesions that interfere with ongoing replication, and similar to CPT can induce DSBs (Deweese and Osheroff, 2009; Liu et al, 2012). BrdU (5-bromo-2′-deoxyuridine) incorporation assays showed that HU and APH blocked replication, while CPT and ETO only partially reduced DNA synthesis (Fig. EV1D). APH induced a similar response to HU in terms of PARylation, CHK1, RPA32, and H2AX phosphorylation after 4 h of treatment (Fig. 1C), confirming that HU acts through inhibiting replication. The combination of APH with PARGi reduced the level of phosphorylated CHK1 and RPA32, but not γH2AX. In contrast, ETO and CPT induced higher pRPA and γH2AX levels but lower pCHK1 signal indicating a response dominated by DNA damage-associated signaling rather than by canonical replication checkpoint activation. In addition, although PARGi reduced CHK1 phosphorylation in both ETO- and CPT-treated cells, it did not markedly decrease the pRPA signal under these conditions (Fig. 1C). In accordance, the reduction in DNA-bound RPA32 in the presence of PARGi was more pronounced in response to fork-stalling agents, such as HU and APH, than to DNA break-inducing agents, such as CPT and ETO (Fig. 1D). Nonetheless, the presence of PARGi led to high levels of PARylation in case of all four tested drugs (Figs. 1C,D and EV2A).

Collectively, these results strengthen that the effect of sustained PARylation depends on the type of replication stress. HU and APH, which impose global fork stalling and strongly engage the canonical ssDNA/RPA/ATR pathway, show the clearest PARGi-dependent reduction in chromatin-bound RPA. In contrast, CPT and ETO generate DSBs and produce a response in which pRPA and γH2AX are more strongly associated with DNA damage signaling, sensitizing cells to the homologous recombination or non-homologous end joining DSB repair pathways, respectively (Chen et al, 2022), making the effect of PARGi on RPA phosphorylation less pronounced. The divergence in stress response suggests different sources of the pRPA signal, depending on the origin of ssDNA they cover: uncoupled forks or resected double-strand breaks.

### Role of PARP activity in the reduction of RPA32 phosphorylation upon HU treatment

**Figure 2:**
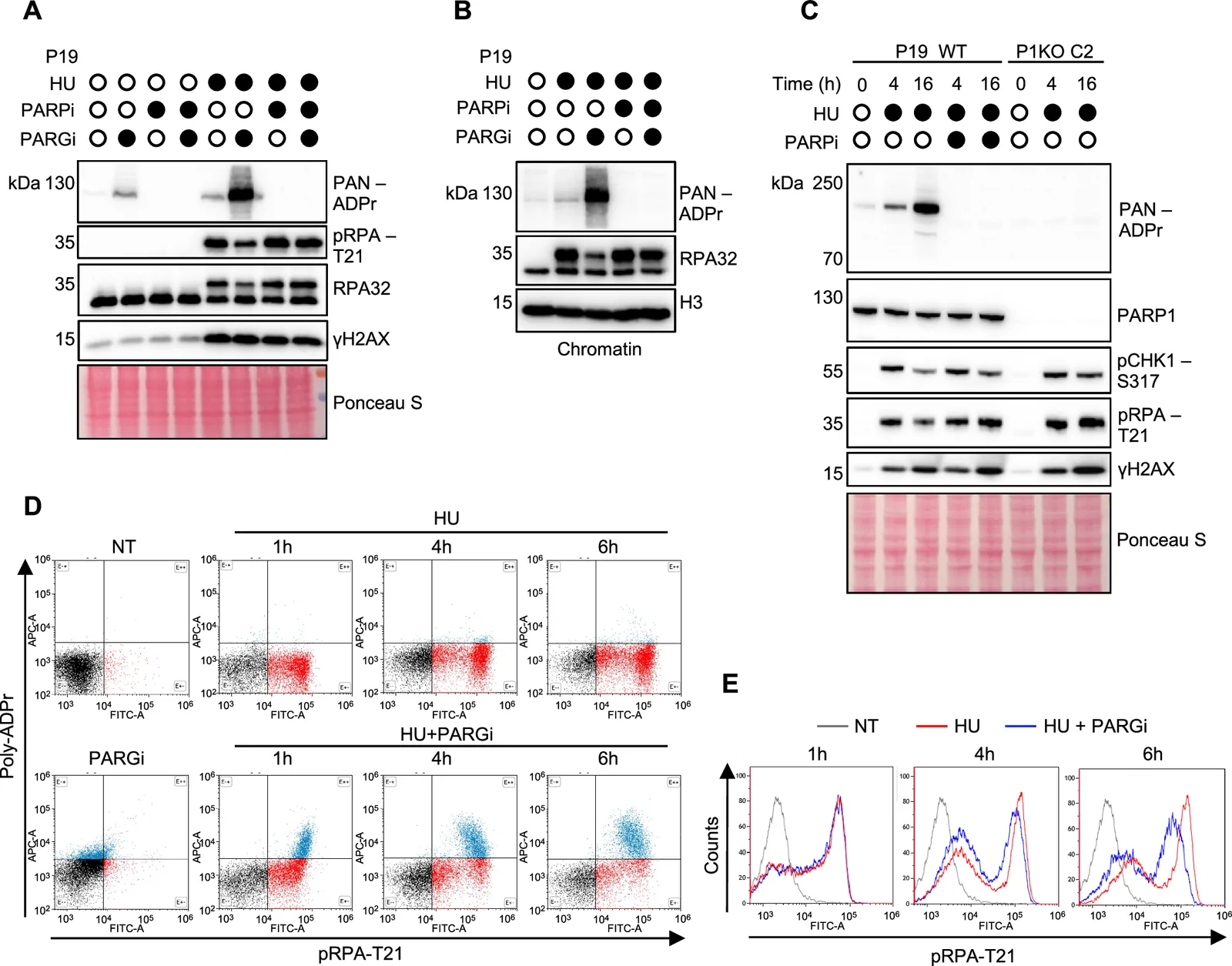
Sustained PARylation and RPA32 phosphorylation are inversely correlated. (**A**) P19 cells were treated with PARGi (5 µM), PARPi (5 µM), and HU (0.5 mM) and all combinations thereof for 4 h, then lysed and analyzed by western blotting with the indicated antibodies. Representative blot from three biological replicates. Samples from the same experiment were run on parallel gels. The corresponding intra-gel Ponceau S staining used as a loading control for each blot are provided in the Source Data file. (**B**) PARylation and RPA32 levels on the chromatin of P19 cells treated with HU (0.5 mM) with or without PARGi (5 µM), PARPi (5 µM) and their combination for 4 h were assessed by western blotting. H3 was used as a loading control. Representative blot from three biological replicates. (**C**) P19 WT and PARP1 KO cells (clone P1KO C2) were treated with HU (0.5 mM) with or without PARPi (5 µM) for the indicated time points, then lysed, and samples were analyzed by western blotting with the indicated antibodies. Representative blot from three biological replicates. Samples from the same experiment were run on parallel gels. The corresponding intra-gel Ponceau S staining used as a loading control for each blot are provided in the Source Data file. (**D**, **E**) P19 cells were treated with HU (0.5 mM) with or without PARGi (5 µM) for the indicated time points, fixed and analyzed by FACS with the indicated antibodies. (**D**) Dot plots of double-stained samples. (**E**) Overlay histograms of pRPA-T21-stained cells. Representative FACS images from three biological replicates are shown. Source data are available online for this figure.

Since the effect of PARG inhibition was strongest upon replication inhibition, we focused the subsequent analysis on HU-treated cells. To confirm the involvement of PARP activity in the cellular response to HU-induced replication stress, we co-treated the cells with the PARPi, Olaparib. PARPi completely blocked PARylation but left the RPA32 and H2AX phosphorylation unaltered during the 4 h co-incubation with HU and abrogated the PARGi-induced reduction in RPA32 phosphorylation (Fig. 2A). Notably, neither PARGi nor PARPi triggered pRPA or γH2AX without HU-treatment, indicating that perturbing PARylation during regular replication did not induce replication stress or DNA damage in P19 at the used concentrations of inhibitors. PARPi also prevented the effect of PARGi in reducing DNA-bound RPA32 (Fig. 2B). The combined treatment of HU, PARPi and PARGi revealed that the excess PAR signal was responsible for the reduced level of pRPA and allowed us to exclude that this arises from an off-target effect of the PARG inhibitor (Fig. 2A,B).

In addition, we generated PARP1 knock-out (P1KO) derivatives of P19 cells using the CRISPR-Cas9 nickase gene-editing approach. Similar to PARP inhibition, PARP1 loss did not noticeably alter the overall replication stress or DNA damage response compared with wild-type cells; however, PARGi failed to reduce RPA32 phosphorylation during HU treatment in the absence of PARP1 (Fig. EV2B). Together, these findings indicate that the effect of PARG inhibition on RPA32 chromatin binding and phosphorylation during HU treatment requires PARP1 activity.

To further test whether excessive PARylation itself influences pRPA levels independently of PARG inhibition, we extended HU treatment to later time points, when PARylation became more prominent even in the absence of PARGi. In wild-type cells, pRPA levels declined over time as PARylation increased, resembling the trend observed upon PARG inhibition (Fig. 2C). To determine whether this effect depends on PARylation, we analyzed PARPi-treated and PARP1 knockout P19 cells, in which HU-induced PARylation is blocked. Under these conditions, prolonged HU treatment led to increased rather than decreased pRPA levels, in contrast to the pattern seen in wild-type cells (Fig. 2C). However, the time-dependent changes in CHK1 and γH2AX phosphorylation were similar irrespective of PARylation status, with pCHK1 decreasing and γH2AX increasing over time. Together, these results are consistent with PARylation contributing to the decline in pRPA during prolonged HU treatment, with PARG inhibition enhancing this effect.

Next, we examined the correlation between PARylation and pRPA-T21 combined with cell cycle analysis of P19 cells by flow cytometry across multiple time points. HU alone induced RPA32 phosphorylation at T21 in S-phase cells from 1 h of incubation, with the signal increasing over time (Figs. 2D and EV2C). Upon co-treatment with PARGi, PAR accumulation became readily detectable (Fig. 2D), and was most pronounced in early S-phase cells (Fig. EV2C). Notably, the pRPA-T21 fluorescence intensity decreased in PAR-positive cells from 4 h of HU and PARGi co-treatment (Fig. 2D,E). Although the percentage of pRPA-T21 positive cells changed only modestly (Fig. EV2C), the decrease of its intensity was clearly detected in cells where PARylation was highest (Fig. 2D), the effect being most evident in early S-phase cells (Fig. EV2C). Single antibody staining of pRPA-T21 confirmed that the reduced signal detection was not due to steric competition between the antibodies (Fig. EV2D). The flow cytometry results support those of western blotting, adding the information that pRPA and ADPr signal comes from the cells striving to replicate their DNA, and that the cells with the highest ADPr have reduced pRPA under prolonged HU treatment. On the whole, we show that continuous replication stress, induced by HU treatment in P19 cells, leads to elevated ADPr in a PARP1-dependent manner, which decreases the chromatin-bound phosphorylated RPA.

### ATR–CHK1 signaling protects from PARylation-associated loss of exposed ssDNA and chromatin-bound RPA during replication stress

**Figure 3:**
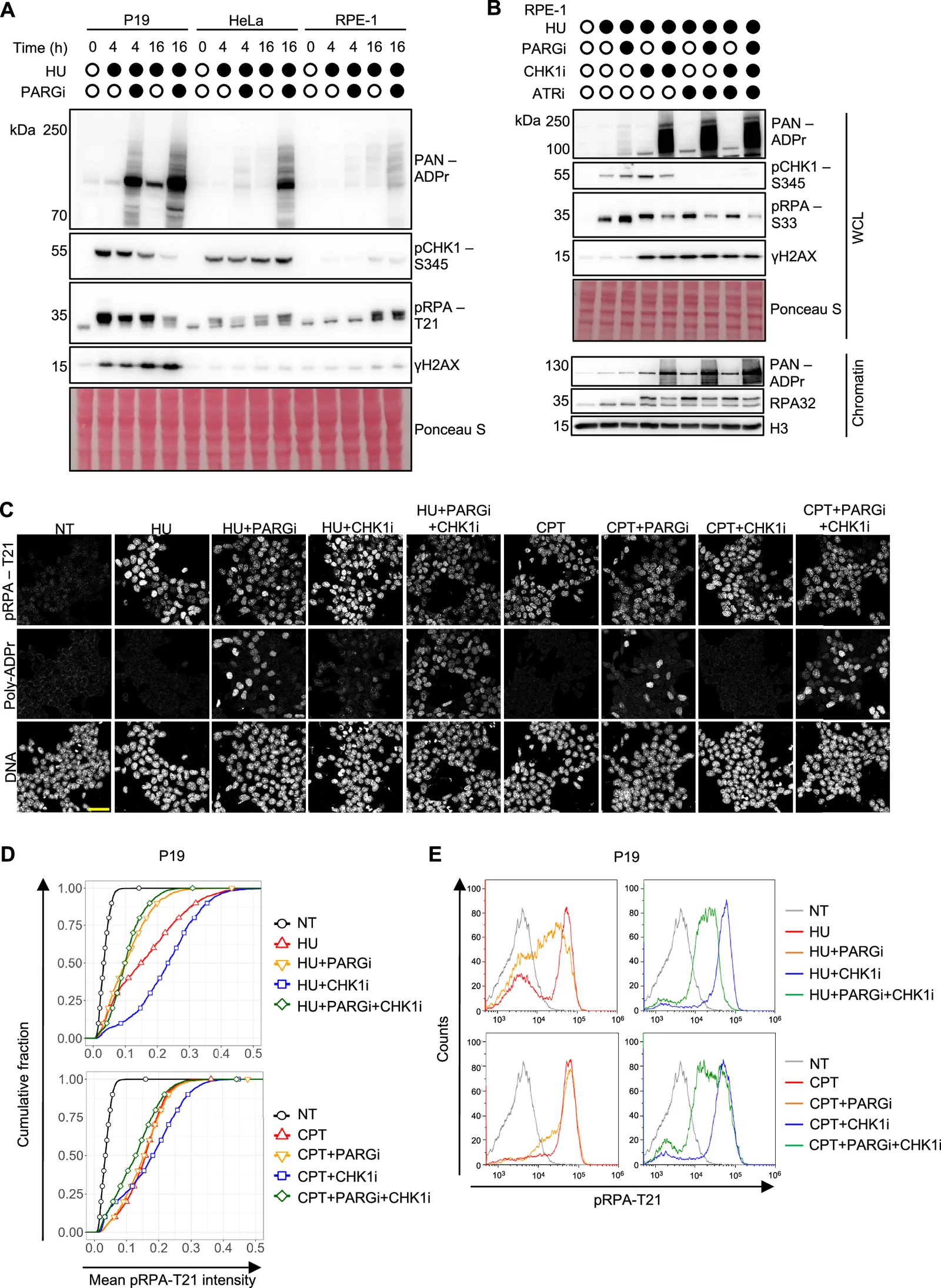
Correlation between ATR-checkpoint loss and pRPA reduction. (**A**) P19, HeLa, or RPE-1 cells were treated with HU (0.5 mM for P19 or 1 mM for HeLa and RPE-1) in the presence or absence of PARGi (5 µM) for the indicated time points, lysed and analyzed by western blotting with the indicated antibodies. Ponceau S staining was used as a loading control. Representative blot from 2 biological replicates. (**B**) RPE-1 cells were treated with HU (1 mM) in the presence or absence of PARGi (5 µM), CHKi (5 µM), and ATRi (5 µM) for 16 h. The whole cell lysates (upper panel) or chromatin fractions (lower panel) of the samples were analyzed by Western blotting with the indicated antibodies. Ponceau S and H3 staining was used as a loading control for whole cell lysates and chromatin fractions, respectively. Representative blot from 3 biological replicates. (**C**, **D**) PAR and pRPA levels of P19 cells treated with HU (0.5 mM) and CTP (5 µM) with or without PARGi (5 µM) in the presence or absence of CHK1i (5 µM) for 4 h, assessed by immunofluorescence. Micrographs of triple-stained samples (**C**, scale bar 50 µm) and the cumulative fractions of mean pRPA-T21 intensities (**D**) are shown for a representative image set from two biological replicates. The quantification of randomly selected 2000 nuclei per condition were plotted. (**E**) P19 cells treated with HU (0.5 mM) and CTP (5 µM) with or without PARGi (5 µM) in the presence or absence of CHK1i (5 µM) for 4 h, then fixed, stained and analyzed by FACS. Overlay histograms of pRPA-T21-stained cells are shown for a representative from three biological replicates. Source data are available online for this figure.

Previous work showed that prolonged HU treatment can impair RPA32 hyperphosphorylation in PARG-depleted HeLa cells (Illuzzi et al, 2014), suggesting that sustained PARylation may interfere with the replication stress response under specific conditions. Therefore, we compared the response of P19 cells to HU with that of HeLa and p53-deficient human retinal pigment epithelium (RPE-1 *TP53*^*−/−*^) cells after short- and long-term incubation (Fig. 3A). In P19 cells, both CHK1 and RPA32 phosphorylation declined between 4 and 16 h of incubation with HU. This decrease was accompanied by increasing γH2AX and PARylation signals. PARG inhibition further reduced CHK1 and RPA32 phosphorylation at both time points in P19, without altering γH2AX levels. In contrast, neither HeLa nor RPE-1 cells showed this pattern. In both HeLa and RPE-1 cells, CHK1 phosphorylation slightly increased over time (Fig. 3A). Notably, HU-induced γH2AX was observed only in P19 cells, where it was already detectable at early time points and increased further with incubation time, whereas γH2AX was not induced in either HeLa or RPE-1 cells. Robust PARylation was also not induced by HU in RPE-1 cells, while in HeLa cells PARylation became apparent only after prolonged treatment and did not coincide with reduced CHK1 or RPA phosphorylation (Fig. 3A). These data indicate that, unlike in HeLa and RPE-1 cells, checkpoint signaling is not maintained during prolonged HU treatment in P19 cells, and this is accompanied by progressive γH2AX accumulation, consistent with the accumulation of damaged or collapse-prone replication fork intermediates.

**Figure EV3:**
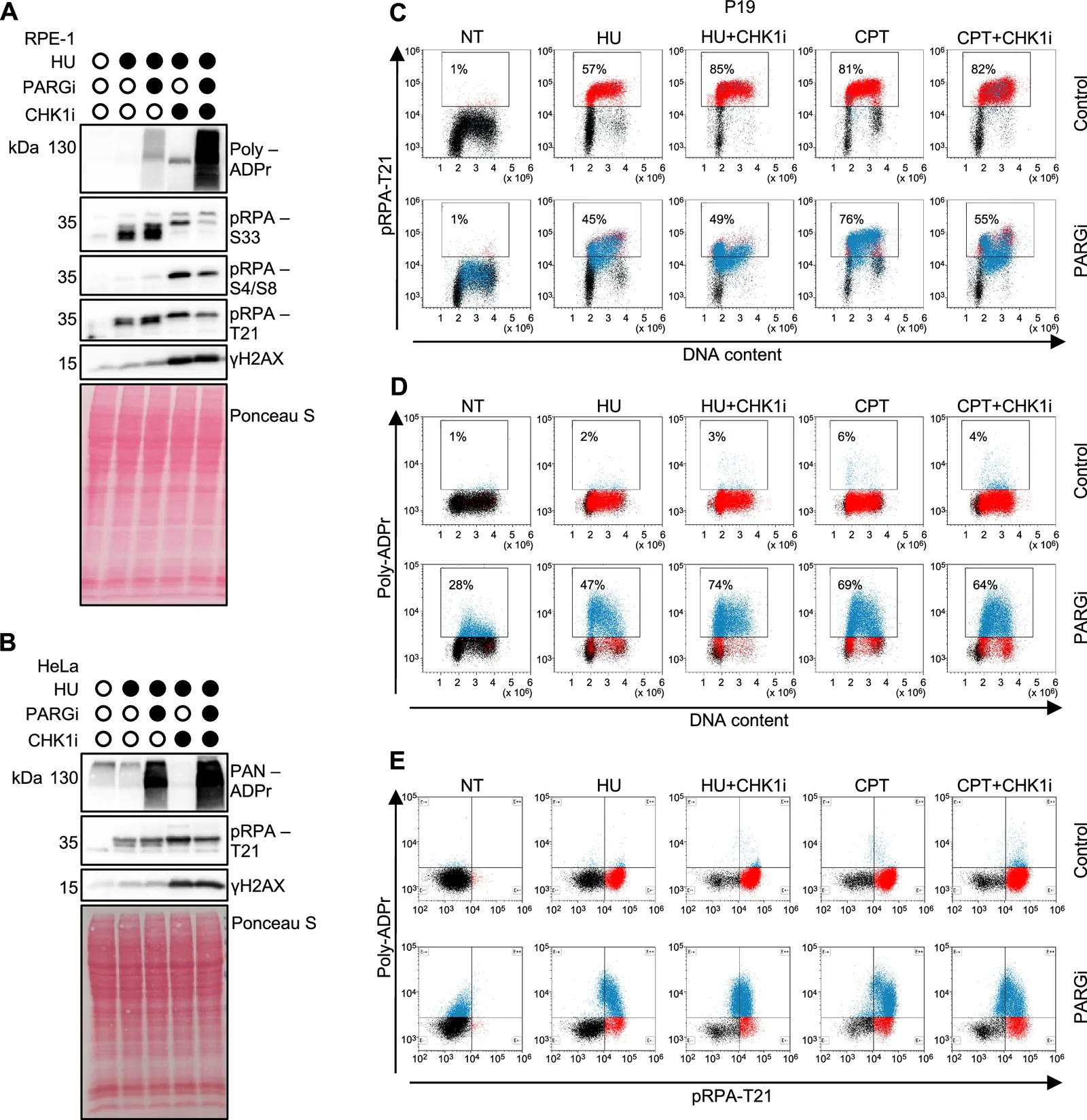
CHK1 inhibition promotes PARGi-induced pRPA reduction under HU treatment in different cell lines. (**A**) RPE-1 cells were treated with HU (1 mM) in the presence or absence of PARGi (5 µM) and CHKi (5 µM) for 24 h. Cells were lysed and samples analyzed by western blotting with the indicated antibodies. Representative blot from 3 biological replicates. Samples from the same experiment were run on parallel gels. The corresponding intra-gel Ponceau S staining used as a loading control for each blot are provided in the Source Data file. (**B**) HeLa cells were treated with HU (1 mM) in the presence or absence of PARGi (5 µM) and CHKi (5 µM) for 24 h. Cells were lysed, and the samples were subjected to western blotting with the indicated antibodies. Ponceau S staining was used as a loading control. Representative blot from 3 biological replicates. (**C**, **D**, **E**) P19 cells treated with HU (0.5 mM) and CTP (5 µM) with or without PARGi (5 µM) in the presence or absence of CHK1i (5 µM) for 4 h, then fixed, stained and analyzed by FACS. FACS images of a representative from three biological replicates are shown. Source data are available online for this figure.

Next, we addressed whether the lack of a PARGi-sensitive reduction in pRPA and chromatin-bound RPA in HU-treated RPE-1 and HeLa cells reflects sustained ATR–CHK1 signaling. In HU-treated RPE-1 cells, PARGi alone did not reduce the phosphorylated forms of RPA32 or CHK1; these signals were maintained or slightly increased (Figs. 3B and EV3A). HU alone or in combination with PARGi did not induce γH2AX; it was only elevated when either ATRi or CHK1i was added to HU treatment, but it was not altered by PARG inhibition. In addition, robust PARylation and the reduction of pRPA and pCHK1 were evident when PARGi was combined with either ATRi or CHK1i during HU treatment (Figs. 3B and EV3A). Combined ATR and CHK1 inhibition produced a response similar to ATR inhibition alone, consistent with ATR acting upstream of CHK1. Analysis of chromatin fractions showed the same pattern, i.e., the chromatin-bound RPA was reduced only when PARGi was combined with ATRi and/or CHK1i, but not with PARGi alone (Fig. 3B). We also examined the effect of CHK1 inhibition in HeLa cells during HU treatment (Fig. EV3B) and observed similar results as in RPE-1 cells regarding PAR, pRPA-T21 and γH2AX signal. Thus, acute ATR–CHK1 inhibition rendered RPE-1 and HeLa cells permissive for the PARGi-associated reduction in pRPA and chromatin-bound RPA observed in P19 cells.

In P19 cells, PARG inhibition reduced pRPA more strongly under HU than under CPT treatment (Fig. 1). This raised the possibility that checkpoint signaling remains more effective during CPT-induced replication stress and limits the emergence of the PARGi-sensitive phenotype. We, therefore, examined the effect of CHK1 inhibition in P19 cells (Figs. 3C–E and EV3C–E). The immunofluorescence and flow cytometry analyses showed that CHK1 inhibition enhanced the reduction in pRPA in both HU- and CPT-treated P19 cells. Notably, during CPT treatment, CHK1 inhibition enhanced the PARGi-dependent reduction in pRPA, extending this checkpoint-dependent sensitivity beyond HU-induced fork stalling (Figs. 3C–E and EV3C–E).

**Figure 4:**
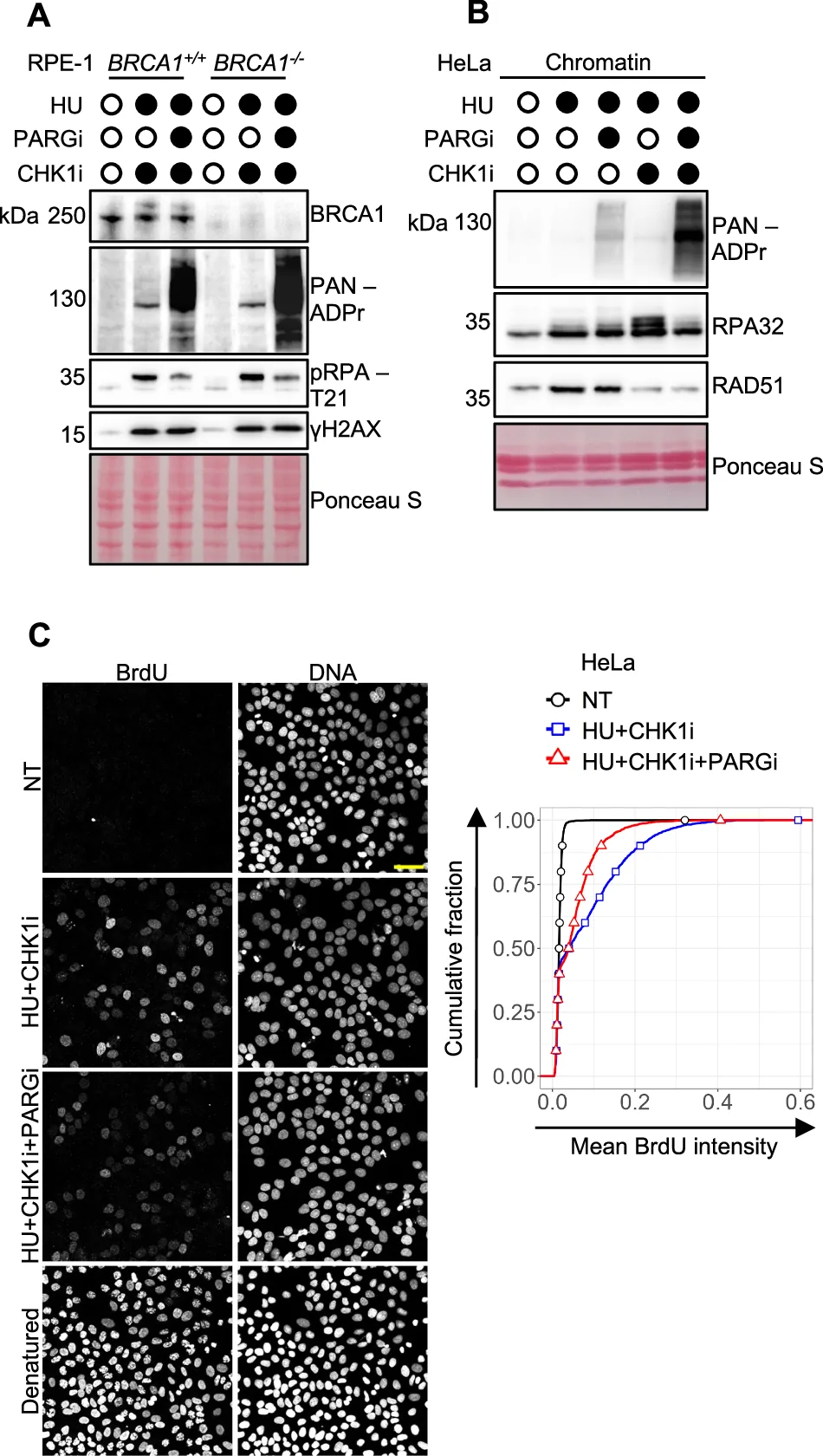
Examination of the mechanisms leading to pRPA reduction. (**A**) BRCA1-proficient (*BRCA1*^*+/+*^) and BRCA1-deficient (*BRCA1*^*−/−*^) RPE-1 cells were treated with HU (1 mM) in the presence or absence of PARGi (5 µM) and CHKi (5 µM) for 24 h. Cells were lysed and samples analyzed by western blotting with the indicated antibodies. Ponceau S staining was used as a loading control. Representative blot from 3 biological replicates. (**B**) HeLa cells were treated with HU (1 mM) in the presence or absence of PARGi (5 µM) and CHKi (5 µM) for 16 h. Chromatin fractionation and western blotting was performed to assess RAD51 and RPA32 levels, and their change in the presence of PARylation. Ponceau S staining was used as a loading control. Representative blot from 3 biological replicates. (**C**) HeLa cells were labeled with BrdU (10 µM) for 48 h and then treated with HU (1 mM) and CHK1i (5 µM) in the presence or absence of PARGi (5 µM) for 24 h. ssDNA was detected by BrdU immunofluorescence. Scale bar is 50 µm. Representative staining from three biological replicates. The quantification of randomly selected 2000 nuclei per condition were plotted. Source data are available online for this figure.

Reduced chromatin-bound RPA could reflect a BRCA1/RAD51-dependent transition of stressed forks into a protected or recombination-associated state, potentially involving replacement of RPA by RAD51. Therefore, we tested whether the PARGi-sensitive reduction in chromatin-bound RPA depends on BRCA1 or is accompanied by increased RAD51 chromatin-loading. In BRCA1-deficient RPE-1 cells, pRPA-T21 was still reduced under checkpoint-compromised, PARG-inhibited conditions (Fig. 4A), indicating that this phenotype does not require BRCA1. In addition, RAD51 was detectable on chromatin after long HU treatment of HeLa cells, and its level was not altered by PARG inhibition (Fig. 4B). CHK1 inhibition reduced chromatin-associated RAD51, consistent with the reported role of CHK1 in RAD51 recruitment (Sørensen et al, 2005), but PARGi did not further affect RAD51 levels on the chromatin, in spite of reduction of chromatin-bound RPA32 (Fig. 4B). Together, these results argue against a model in which the PARGi-dependent reduction in pRPA is driven by BRCA1-dependent fork protection and replacement by RAD51.

**Figure EV4:**
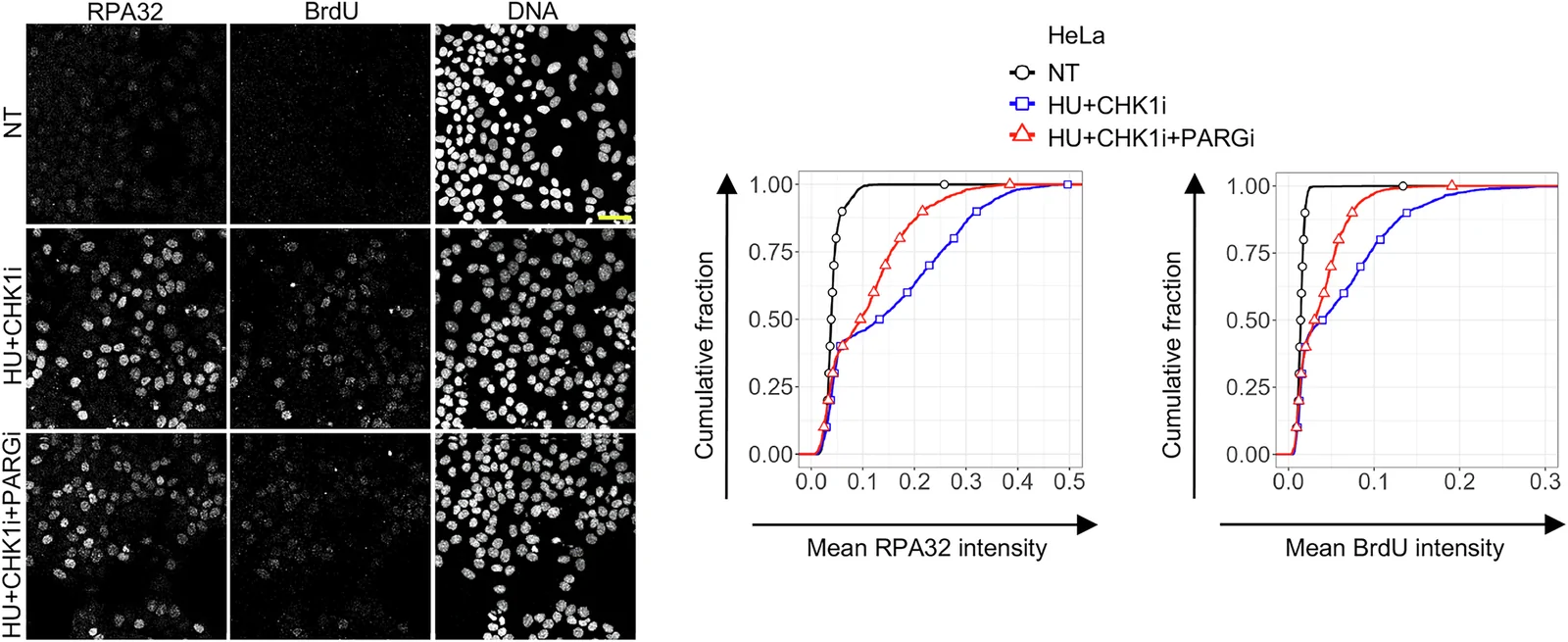
Correlation of chromatin-bound RPA32 and ssDNA. HeLa cells were labeled with BrdU (10 µM) for 48 h and then treated with HU (1 mM) and CHK1i (5 µM) in the presence or absence of PARGi (5 µM) for 24 h. ssDNA was detected by BrdU and RPA32 immunofluorescence. Scale bar is 50 µm. Representative staining from three independent experiments. The quantification of randomly selected 2000 nuclei per condition were plotted. Source data are available online for this figure.

Reduced pRPA and chromatin-bound RPA could alternatively reflect the reduced ssDNA exposure; therefore, we tested this directly by native BrdU staining in HeLa cells under conditions in which CHK1 inhibition renders the HU response sensitive to PARGi (Figs. 4C and EV4). Consistent with the changes in chromatin-associated RPA, addition of PARGi to HU and CHK1i co-treated cells reduced the native BrdU signal, indicating that sustained PARylation decreases ssDNA levels under checkpoint-compromised conditions.

Together, these data indicate that ATR–CHK1 signaling limits the emergence of a PARGi-sensitive replication stress state. Across the tested cellular and stress contexts, reduced or blocked checkpoint signaling rendered cells susceptible to sustained PARylation accompanied by reduced pRPA, reduced chromatin-bound RPA, and reduced exposed ssDNA.

### HU induces early replisome destabilization and reliance on new origin firing in P19 cells

**Figure 5:**
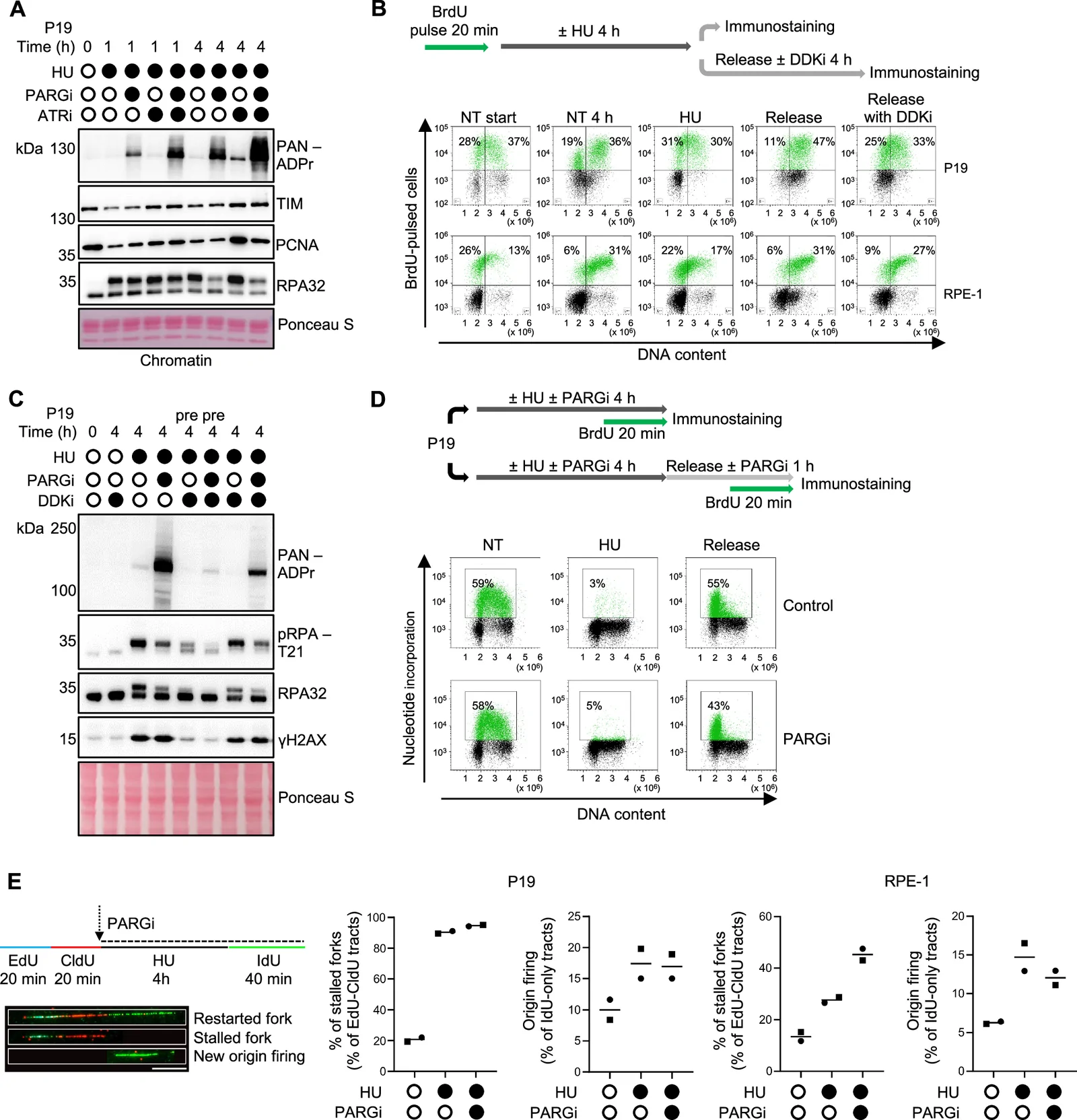
Detection of HU-induced fork collapse and new origin firing. (**A**) P19 cells were treated with HU (0.5 mM) with or without PARGi (5 µM) in the presence or absence of ATRi (5 µM) for 1 or 4 h and subjected to chromatin fractionation. PARylation, TIM, PCNA and RPA32 levels were assessed by western blotting. Representative blot from three biological replicates. Samples from the same experiment were run on parallel gels. The corresponding intra-gel Ponceau S staining used as loading control for each blot are provided in the Source Data file. (**B**) P19 and RPE-1 cells were incubated with BrdU (NT start) to pulse-label the S-phase cells and then treated with HU (0.5 mM) for 4 h in the presence or absence of DDKi (1 µM), or left untreated. The cells were then fixed or released for 4 h. The propagation of labeled cells throughout the cell cycle was followed by anti-BrdU and propidium iodide (DNA content) staining and analyzed with flow cytometry. Representative FACS analysis from 3 biological replicates are shown. (**C**) P19 cells were treated with HU (0.5 mM) with or without PARGi (5 µM) for 4 h. DDKi (1 µM) was either pre-incubated for 4 h (lanes 5 and 6) with the HU or added at the same time (lanes 7 and 8). Cells were lysed, and the samples were subjected to western blotting by the indicated antibodies. Representative blot from three biological replicates. Samples from the same experiment were run on parallel gels. The corresponding intra-gel Ponceau S staining used as a loading control for each blot are provided in the Source Data file. (**D**) P19 cells were treated with HU (0.5 mM) with or without PARGi (5 µM) for 4 h. The cells were then fixed immediately or released for 1 h. BrdU was added for the last 20 min before fixation. The samples were stained with anti-BrdU (nucleotide incorporation) and propidium iodide (DNA content), and then analyzed with flow cytometry. FACS images of a representative experiment from three biological replicates are shown. (**E**) DNA fiber assay of P19 or RPE-1 cells, treated with HU (0.5 mM for P19 or 1 mM for RPE-1) with or without PARGi (5 µM) for 4 h. Left: Scheme of DNA fiber assay labeling and representative images of DNA replication tracts. Scale bar: 10 µm. Right: percentage of the stalled replication forks (EdU-CldU tracts) or the IdU-only tracts (reflecting new origin firing) from two biological replicates shown with different symbol shapes; black lines indicate the mean value. At least 300 DNA replication tracts were quantified per replicate. Source data are available online for this figure.

**Figure EV5:**
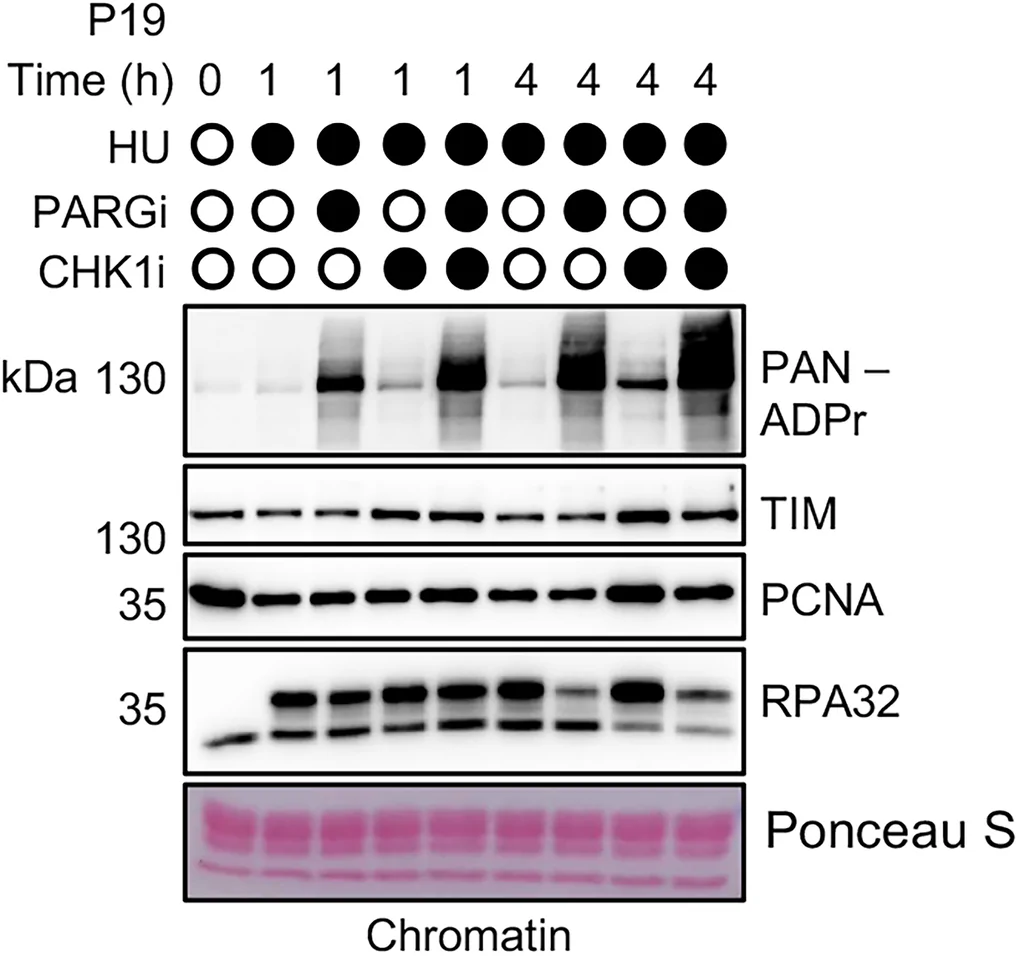
CHK1 inhibition increases the level of chromatin-bound TIM and PCNA. P19 cells were treated with HU (0.5 mM) with or without PARGi (5 µM) in the presence or absence of CHK1i (5 µM) for 1 or 4 h and subjected to chromatin fractionation. PARylation, TIM, PCNA, and RPA32 levels were assessed by western blotting. Representative blot from three biological replicates. Samples from the same experiment were run on parallel gels. The corresponding intra-gel Ponceau S staining used as a loading control for each blot are provided in the Source Data file. Source data are available online for this figure.

In most cell types, short HU treatment causes reversible replication fork stalling, whereas prolonged HU exposure leads to fork collapse and requires new origin firing for the recovery of DNA replication (Petermann et al, 2010). In our earlier experiments, PARGi-mediated reduction of pRPA in RPE-1 cells was observed only under checkpoint-compromised conditions and after longer HU treatment, suggesting that this phenotype emerges at later stages of stalled-fork failure rather than during the initial response. By contrast, in P19 cells, the chromatin-associated levels of Timeless (TIM) and proliferating cell nuclear antigen (PCNA), two components of the active replisome, were already reduced after 1 h of HU treatment (Fig. 5A), indicating disintegration of the replisome. Inhibition of ATR (Fig. 5A) or CHK1 (Fig. EV5) increased the amount of chromatin-bound TIM and PCNA in HU-treated P19 cells, consistent with increased assembly of replication machinery at newly fired origins. ATRi and CHK1i also increased chromatin-bound RPA32, but did not prevent its PARGi-mediated reduction, which remained most evident after 4 h of HU treatment (Figs. 5A and EV5).

These observations suggested that HU treatment drives unusually rapid fork failure in P19 cells and that loss of sustained ATR–CHK1 signaling may permit compensatory origin firing. To test this, we pulse-labeled S-phase cells with BrdU and followed their cell cycle progression using propidium iodide staining and flow cytometry. Untreated BrdU-labeled P19 cells reached late S/G2 within 4 h, and a fraction had already completed mitosis and entered the next G1 phase (Fig. 5B). In contrast, HU-treated cells failed to increase their DNA content unless released from the block. Because activation of new replication origins requires Cdc7/Dbf4-dependent kinase (DDK), we tested whether DNA synthesis after HU release depends on DDK activity. Indeed, DDK inhibition abolished DNA replication after release from HU in P19 cells (Fig. 5B), indicating that recovery requires new origin firing. This was not the case in RPE-1 cells, whose progression after release from a 4 h HU block was not prevented by DDK inhibition (Fig. 5B), proving the contribution of fork restart in this cell line.

We next asked whether the replication stress signals observed in P19 cells arise from ongoing forks, newly fired origins, or both. Preventing cells from entering S-phase by DDKi pre-incubation rendered P19 cells largely insensitive to HU, with only minimal PARylation, pRPA-T21, and γH2AX signals (Fig. 5C). By contrast, simultaneous addition of DDKi with HU, which allows only pre-existing forks to experience stress, produced an intermediate response. These results indicate that the HU-induced signals in P19 cells arise from both stalled replication forks and newly fired origins. Consistent with this, when cells were released after 4 h HU treatment, and BrdU was added during the final 20 min of a 1 h recovery period, nucleotide incorporation was reduced by PARGi (Fig. 5D), suggesting that sustained PARylation limits DNA synthesis after release, either by impairing new origin firing or by slowing replication from newly established forks.

We verified these conclusions by DNA fiber analysis using a three-label scheme of EdU, CldU, 4 h HU, and post-release IdU. This labeling strategy allows us to clearly distinguish HU-stalled replication forks (EdU-CldU) from forks that restart (EdU-CldU-IdU). In P19 cells, 4 h exposure to HU led to massive replication collapse, as we detected that approximately 90% of forks stalled, thus only 10% of replication forks could resume after HU washout (Fig. 5E). In contrast, more than 70% of forks in RPE-1 cells resumed replication after HU washout (Fig. 5E), indicating that replication fork protection in RPE-1 cells is more robust. The percentage of stalled replication forks elevated in the presence of PARGi in RPE-1 cells, suggesting increased replication fork collapse due to excessive PARylation. This method also enables us to detect novel origin firing by quantifying the frequency of IdU-only tracts, which represent origins that fired after HU washout. We observed PARGi-independent increased origin firing after release from HU in both RPE-1 and P19 cells. Together, the fiber data indicate that RPE-1 cells recover from HU using both restart of stalled forks and new origin firing, whereas P19 cells rely predominantly on new origin firing because most stalled forks fail to resume replication.

Altogether, these experiments indicate that P19 cells are remarkably sensitive to HU-induced replication stress. Early loss of CHK1 signaling is accompanied by replisome destabilization, γH2AX accumulation, and poor fork restart, consistent with rapid fork collapse. Under these conditions, DNA synthesis after HU release becomes strongly dependent on DDK activity, indicating reliance on new origin firing as a salvage pathway. Together with the reduced ssDNA and chromatin-bound RPA observed under PARGi-sensitive conditions, these findings support a model in which persistent PARylation promotes fork destabilization and loss of restart competence rather than merely altering checkpoint signaling. In contrast, RPE-1 cells largely preserve stalled forks during 4 h HU treatment and retain a much greater capacity for fork restart after release.

## Discussion

We investigated how sustained poly(ADP-ribosyl)ation shapes cellular responses to replication stress. We found that its consequences depend strongly on the nature of the stress and the cellular context. Endogenous S-phase PAR has been linked to incompletely processed Okazaki fragments, and PARP inhibition has been shown to impair maturation of nascent DNA strands and promote postreplicative nicks or gaps, particularly when canonical Okazaki-fragment processing is perturbed (Hanzlikova et al, 2018; Vaitsiankova et al, 2022). On the other hand, our study revealed that PARylation reduces ssDNA levels and, through lowering ssDNA-bound RPA it supresses RPA-mediated signaling during replication fork collapse.

Different replication stressors and DNA damage-inducing agents reveal that PARylation levels do not always correlate with the amount of RPA reduction on the chromatin, as the effect of PARG inhibition was strongly stress-type dependent. Although all four agents tested led to detectable PARylation in the presence of PARGi, the most pronounced reduction in chromatin-bound RPA and CHK1 phosphorylation occurred with HU and APH, which rapidly inhibit DNA synthesis and strongly engage the canonical ssDNA/RPA/ATR pathway. By contrast, camptothecin and etoposide impose DNA breaks and elicit a response in which γH2AX and pRPA are more strongly shaped by DNA damage signaling. Thus, sustained PARylation does not affect all replication stress responses equally but is most clearly linked to DNA-bound RPA reduction under fork-stalling conditions that progress into a damaged, recovery-defective fork collapse.

Since PARG inhibition showed the most significant effect under HU treatment, we focused much of our mechanistic analysis on this condition. PARPi completely blocked PARylation but did not suppress HU-induced γH2AX or pRPA on its own, while abrogating the PARGi-induced decrease in RPA phosphorylation and chromatin binding. Similarly, PARP1 loss prevented the effect of PARGi on RPA phosphorylation. Moreover, when HU treatment was prolonged in the absence of PARGi, pRPA declined over time in wild-type cells as PARylation increased, whereas blocking PARylation genetically or pharmacologically shifted the response in the opposite direction. Together, these findings indicate that excessive PARylation contributes to the decline in chromatin-bound RPA during prolonged HU treatment and that PARG inhibition amplifies this effect, arguing that the phenotype reflects deregulated PAR turnover rather than a mere pharmacological artifact of inhibitor co-treatment.

We found that checkpoint status dictated whether fork stalling transitioned into the PARGi-sensitive phenotype. In P19 cells, HU alone rapidly triggered fork collapse, as reflected by the early loss of TIM and PCNA from chromatin, progressive γH2AX accumulation, poor fork restart, and reliance on new origin firing after release. In contrast, HeLa and RPE-1 cells maintained stalled forks for longer during the HU response, and PARGi alone was not sufficient to reduce pRPA or chromatin-bound RPA under these conditions. However, once ATR or CHK1 was inhibited in RPE-1 or HeLa cells, robust PARylation together with reduced pRPA and lower chromatin-bound RPA became evident, recapitulating the phenotype observed in P19 cells. These findings suggest that the effect of PARGi is not determined simply by the amount of PAR formed, but by whether forks remain protected or instead collapse.

This interpretation also helps reconcile our findings with previous work showing that PAR can promote CHK1 retention and efficient checkpoint activation at stalled replication forks (Min et al, 2013). In that setting, PAR supports the checkpoint response to newly stalled forks. Our data address a different regime, in which replication stress persists, and checkpoint maintenance fails, and the system transitions into a PARylation-associated state with reduced pRPA, reduced chromatin-bound RPA, and reduced ssDNA. Thus, PAR may facilitate early checkpoint signaling at stalled forks while having different consequences once replication stress persists and damaged-fork states accumulate. Distinguishing between early checkpoint-supportive PAR signaling and later PARylation-associated fork dysfunction is likely to be important for understanding the context-specific outcomes of PARG inhibition.

Similar to the findings of Illuzzi et al (Illuzzi et al, 2014), we find that excessive PAR accumulation under prolonged HU-related stress reduces RPA loading on the DNA. However, in our system, under conditions in which checkpoint suppression renders HU-treated HeLa and RPE-1 cells sensitive to PARGi, the reduction in chromatin-bound RPA is accompanied by a reduction in ssDNA, as revealed by the low native BrdU signal. This argues for a model in which the reduction in chromatin-associated RPA is due to a reduced amount of exposed ssDNA. In addition, the phenotype does not require BRCA1 and is not accompanied by increased RAD51 loading, opposing straightforward replacement of RPA by RAD51 at stressed forks. Thus, whereas the conditions investigated by Illuzzi et al caused impaired RPA accumulation on ssDNA-containing structures and persistent CHK1 signaling in the high-PAR population, we identified that upon fork collapse, PARG inhibition is associated with reduced ssDNA exposure, lower RPA occupancy, and impaired restart competence. These differences may reflect that excessive PAR accumulation can accompany more than one damaged-fork state, depending on whether ssDNA-containing structures and checkpoint signaling are still maintained.

PARP1 activity and PARylation have been linked to fork remodeling and slowed restart through PAR-dependent inhibition of RECQ1, which normally promotes the restart of reversed forks (Berti et al, 2013). Although fork reversal is catalyzed primarily by RAD51 and fork-remodeling translocases such as HLTF, SMARCAL1, and ZRANB3, PARylation appears to influence this response in part through RECQ1-dependent fork restart (Zellweger et al, 2015). We did not directly measure fork reversal in the present study and therefore cannot assign the PARGi-sensitive phenotype to a defined remodeling intermediate. Nevertheless, the reduced native BrdU signal, the decrease in chromatin-bound RPA, and the reduced BrdU incorporation during recovery from HU are all compatible with the possibility that sustained PARylation promotes fork reversal with increased strand reannealing, reduced parental ssDNA exposure, and consequently diminished RPA loading. Sustained PARylation may also reduce ssDNA levels by limiting nuclease-dependent processing of stalled or reversed forks. Several nucleases, including CtIP–MRE11, EXO1 and DNA2, have been implicated in the processing of replication stress-induced fork intermediates, and such processing can generate ssDNA substrates for RPA binding (Pasero and Vindigni, 2017; Lemaçon et al, 2017; Thangavel et al, 2015). Thus, the reduced chromatin-bound RPA observed after PARG inhibition could arise from at least two complementary mechanisms: increased strand reannealing during fork reversal and reduced nuclease-mediated ssDNA generation at stalled or reversed forks.

Recent work indicates that complete loss of PARG activity is lethal in proliferating cells with ongoing PARP1 activity (Nie et al, 2024). Acute pharmacological PARG inhibition does not necessarily phenocopy the complete loss of PARG activity, which may contribute to the context dependence observed here. It is possible that at the PARG inhibitor concentrations used here, residual PARG activity is sufficient during unperturbed replication but becomes limiting once HU-treated forks progress into a high-PAR, recovery-defective state. This framework helps explain why PARG inhibitor responses vary across model systems and suggests that clinically relevant PARGi toxicity may emerge preferentially in settings where replication stress is coupled to defective checkpoint maintenance or unstable fork recovery (Pillay et al, 2019).

## Methods

**Table:** Reagents and tools table

Reagent/resource	Reference or source	Identifier or catalog number
**Experimental models**		
P19	Previous study, 10.1007/BF01233072	NA
P19 PARP1 KO C1	This study	NA
P19 PARP1 KO C2	This study	NA
HeLa	ATCC	CCL-2
RPE-1 TP53^-/-^	Previous study, 10.1016/j.molcel.2018.10.045	NA
RPE-1 TP53^−/−^ BRCA1^−/−^	Previous study, 10.1016/j.molcel.2018.10.045	NA
**Recombinant DNA**		
pSpCas9n(BB)-2A-Puro (PX462) V2.0	Addgene	Cat# 62987
pUC-H1-gRNA	Addgene	Cat# 61089
**Antibodies**		
Rabbit anti-H3	Proteintech	17168-1-AP
Mouse anti-PCNA	Santa Cruz	sc-56
Rabbit anti-Timeless	Bethyl	A300-961A
Rabbit anti-RPA32	Bethyl	A300-244A
Rabbit anti-Rad51	Abcam	ab133534
Mouse anti-Chk1	Cell Signaling	2360
Rabbit anti-PARP1	Abcam	ab32138
Rabbit anti-BRCA1	Proteintech	22362-1-AP
Rabbit anti-pRPA-T21	Abcam	ab109394
Rabbit anti-pRPA S33	Bethyl	A300-246A
Rabbit anti-pRPA S4/S8	Bethyl	A300-245A
Rabbit anti-pChk1 S345	Cell Signaling	12268
Rabbit anti-pChk1 S317	Cell Signaling	2344
Rabbit anti-pChk2 Th68	Proteintech	29012-1-AP
Rabbit anti-pan(ADP-ribose)	Milipore	MABE1016
Mouse anti-Poly(ADP-ribose)	Enzo Life Sciences	ALX-804-220-R100 (10H)
Rabbit anti-γH2A.X	Abcam	ab81299
Mouse anti-γH2A.X	Invitrogen	MA1-2022
Mouse anti-BrdU	Santa Cruz	sc-32323
Mouse anti-BrdU	BD Biosciences	347580
Rat anti-BrdU	Abcam	ab6326
Rabbit anti-biotin	Abcam	ab6326
Swine anti-rabbit Ig-HRP	Dako	P0399
Goat anti-mouse IgG-HRP	Invitrogen	A16066
Goat anti-rabbit IgG Alexa Fluor 555	Invitrogen	A21428
Goat anti-rabbit IgG Alexa Fluor 488	Invitrogen	A11008
Goat anti-mouse IgG Alexa Fluor 488	Invitrogen	A11001
Goat anti-mouse IgG Alexa Fluor 555	Invitrogen	A21422
Goat anti-rat IgG Alexa 555	Invitrogen	A21434
Goat anti-mouse IgG Alexa Fluor 488	Invitrogen	A11001
Donkey anti-rabbit Alexa Fluor 647	Invitrogen	A31573
**Oligonucleotides and other sequence-based reagents**		
gRNA1	Eurofins Genomics	5’ –GGTGCCGTCAGGAGAGTCAG– 3’
gRNA2	Eurofins Genomics	5’ –CAGCTCCTTCAGGTCGTTGG– 3’
gRNA3	Eurofins Genomics	5’ –CGACGCTTATTACTGTACTG– 3’
gRNA4	Eurofins Genomics	5’ –TGGCCTGAACACTCCTTGCA– 3’
**Chemicals, enzymes and other reagents**		
Hydroxyurea	Sigma-Aldrich	H8627
Aphidicolin	Thermo Scientific	J60236.MCR
Camptothecin	Sigma-Aldrich	C9911
Etoposide	Sigma-Aldrich	E1383
PDD 00017273 (PARGi)	MedChem Express	HY-108360
AZD2281 (Olaparib, PARPi)	Selleck Chemicals	S1060
MK-8776 (Chk1i)	MedChem Express	HY-15532
VE-821(ATRi)	Selleck Chemicals	S8007
KU-55933 (ATMi)	Selleck Chemicals	S1092
NU7441 (DNA-PKi)	Selleck Chemicals	S2638
TAK-931(CDC7i, DDKi)	MedChem Express	HY-100888
DharmaFECT 1	Horizon	T-2001-02
Pierce™ Universal Nuclease for Cell Lysis	Thermo Scientific	88701
Subcellular Protein Fractionation Kit	Thermo Scientific	78840
cOmplete, Mini, EDTA-free Protease Inhibitor Tablets	Roche	11836170001
TrypLe Select Enzyme	Gibco	12563011
PureLink RNase A	Invitrogen	12091-021
Propidium iodide	Sigma-Aldrich	P4170
Fluoromount-G	Invitrogen	00-4958-02
EdU, 5-ethynyl-2’-deoxyuridine	Invitrogen	A10044
CldU, 5-chloro-2’-deoxyuridine	Sigma-Aldrich	C6891
IdU, 5-iodo-2’-deoxyuridine	Sigma-Aldrich	I7125
Biotin Azide	Click Chemistry Tool	CCT-1265-5
WesternBright ECL HRP substrate	Advansta	DV-K-12045-D50
SuperSignal™ West Pico PLUS Chemiluminescent Substrate	Thermo Scientific	34580
**Software**		
Alliance Q9 Advanced	Uvitec Cambridge	Alliance Q9 Advanced 18.07 - SN
ZEN (Blue edition)	Carl Zeiss Microscopy	Zeiss Zen 2.3 (blue edition)
Leica Application Suite X	Leica Microsystems	Version: 3.10.1.29575
Fiji (ImageJ distribution)	(Schindelin et al, 2012)	1.54p; RRID:SCR_002285
R	R Core Team, Vienna, Austria	https://www.R-project.org
RStudio	Posit Software, PBC, Boston, MA	http://www.posit.co
GraphPad Prism		Version: 10.6.1
Kaluza Flow Cytometry Analysis Software	Beckman Coulter Life Sciences	Version: 2.1.1
CytExpert Software 2.4	Beckman Coulter Life Sciences	Version: 2.4.0.28
**Others**		
NanoDrop Spectrophotometer	NanoDrop Technologies	ND-1000
Zeiss LSM800 confocal microscope	Carl Zeiss Microscopy	
Leica DM6000 fluorescent microscope	Leica Microsystems	
Alliance Q9 Advanced Chemidoc imaging platform	Uvitec Cambridge	
CytoFLEX S flow cytometer	Beckman Coulter Life Sciences	Model: B75442

### Cell lines and culture conditions

The P19 embryonic cell line was a kind gift from Professor István Raskó (Rasko et al, 1993). PARP1 knockout (KO) P19 cells were generated in-house using CRISPR-Cas9 nickase-mediated genome editing. RPE-1 *TP53*^*−/*^^−^ and *TP53*^*−/−*^ *BRCA1*^*−/−*^ cells were generously provided by Alan D. D’Andrea (Lim et al, 2018). The HeLa cell line was authenticated through STR profiling (Eurofins Genomics), showing a 100% match with HeLa (amelogenin + 12 loci) according to the Cellosaurus database (Bairoch, 2018).

RPE-1 cells were cultured in DMEM/F-12, while P19 and HeLa cells were cultured in high-glucose DMEM. Culture media were supplemented with 10% FBS, 100 U/ml penicillin and 100 µg/ml streptomycin. For HeLa cells, the medium was additionally supplemented with non-essential amino acids. Cells were maintained in a humidified incubator at 37 °C with 5% CO_2_.

### CRISPR-Cas9 nickase-mediated knockout generation

We generated PARP1 KOs in the P19 cell line by means of CRISPR-mediated genetic editing. We used a modified version of the CRISPR-Cas9 called D10A or nickase, pSpCas9n(BB)-2A-Puro (PX462) V2.0, which was a kind gift from Feng Zhang's lab (Addgene, plasmid #62987) (Ran et al, 2013). The 4 guide RNAs (gRNA) were cloned into pUC-H1-gRNA, a kind gift from Shenglin Huang's lab (Addgene, plasmid #61089) (Zheng et al, 2014). DharmaFECT 1 was used for the plasmid transfection according to the manufacturer’s instructions. The KOs were validated by PCR and Western blotting. The gRNAs listed in the Reagents and tools table were used to generate a 1 kb deletion spanning over exons 6 and 7 of the mouse PARP1 gene.

### Western blotting

Cells were treated for the indicated time points in fresh culture medium with the drugs indicated in the Figure legends. At the end of the treatment the cells were washed in PBS and lysed with SDS denaturing lysis buffer supplemented with Benzonase (Pierce™ Universal Nuclease for Cell Lysis) (Lysis buffer: 4% SDS, 50 mM Tris-HCl, pH 7.4, 100 mM NaCl, 4 mM MgCl_2_, 5 U Benzonase/3 million cells). Cells were lysed directly on the petri dish after carefully removing the PBS, and the lysates were collected with the help of a cell scraper. Total protein was determined by NanoDrop (A280 setting), after which the samples were equalized and boiled in Laemmli buffer. Lysates were separated on SDS-PAGE and transferred onto a nitrocellulose membrane. After blocking with 5% non-fat dry milk in TBS with 0.1% Tween 20, the membranes were incubated with primary antibodies, followed by the appropriate HRP-coupled secondary antibodies, listed in the Reagents and Tools Table. A potential limitation of the used phospho-specific antibodies is that we cannot exclude the possibility that additional modifications near the phospho-epitope alter the recognition of their phospho-specific target epitopes. The signal was visualized with commercial ECL HRP substrate, according to the manufacturer’s instructions, and imaged with a Uvitec Alliance Q9 Advanced system.

### Chromatin fractionation

The chromatin fraction was isolated using the Thermo Scientific Subcellular Protein Fractionation Kit. Briefly, cells were treated for the indicated time points, washed in PBS and then the ice-cold cytoplasmic extraction buffer supplemented with protease inhibitor cocktail (PIC, cOmplete Mini, EDTA-free Protease Inhibitor), PARGi and Olaparib at a final concentration of 1 µM was added. Cells were scraped, collected and incubated on ice for 10 min with gentle tapping. Cells were centrifuged at 500×*g* for 5 min at 4 °C. Cytoplasmic fraction was collected in prechilled tubes, and the pellet was dissolved and shortly vortexed in ice-cold membrane extraction buffer supplemented with PIC, PARGi and Olaparib. Tubes were incubated on ice for 10 min with gentle tapping and centrifuged at 4000×*g* for 5 min at 4 °C. The pellet was dissolved in ice-cold nuclear extraction buffer supplemented with inhibitors, thoroughly vortexed and then incubated for 30 min on ice with gentle tapping. Centrifugation was done at 5000×*g* for 6 min at 4 °C. Nuclear fraction was collected in prechilled tubes, and the pellet was thoroughly vortexed in nuclear extraction buffer supplemented with MgCl_2_, micrococcal nuclease, PARGi and Olaparib and incubated for 15 min at RT. All fractions were boiled in Laemmli buffer. In addition to the kit-based method, chromatin fractionation was performed by adapting a published protocol (Gillotin, 2018). The final pellet was resuspended in SDS denaturing buffer supplemented with 10 U Benzonase/3 million cells, vortexed and incubated at RT until the viscosity was reduced.

### Fluorescent microscopy

After treatment, cells were washed with PBS and fixed in an ice-cold 7:3 methanol: acetone mixture for 15 min at –20 °C. The fixed cells were washed with PBS and permeabilized with 0.2% Triton X-100 in PBS for 10 min and then blocked with 5% FBS in PBS for 1 h at RT. The samples were incubated with the indicated primary antibodies followed by fluorescent secondary antibodies diluted in blocking solution, and finally DAPI was added (1 μg/ml in PBS). Images were acquired using a Zeiss LSM800 confocal microscope equipped with a Plan-Apochromat 20×/0.8 M27 objective and controlled by ZEN 2.3 software. Fluorescence excitation was performed using diode lasers at 405, 488, and 561 nm.

### Native BrdU immunostaining

Cells were incubated with 10 µM BrdU for 48 h. Before treatment, cells were washed twice with PBS. After treatment with HU, PARGi and CHK1i, the cells were washed with PBS and pre-extracted in 0.2% Triton X-100 in PBS containing PIC, 1 µM Olaparib, and 1 µM PARG inhibitor for 5 min at 4 °C, then immediately fixed in 4% paraformaldehyde with the same supplements for 15 min at 4 °C. Cells were washed three times with PBS, permeabilized in 0.2% Triton X-100 in PBS for 10 min at room temperature, blocked in 5% FBS in PBS for 1 h at room temperature, and incubated overnight at 4 °C with mouse anti-BrdU (Santa Cruz, sc-32323) and anti-RPA32 antibodies diluted in blocking solution. The next day, cells were stained with the appropriate secondary antibody and counterstained with DAPI. To confirm BrdU incorporation, one sample was denatured with 2 N HCl, neutralized with citrate-phosphate buffer (pH 7.4), and then subjected to analysis with anti-BrdU antibody. Images were acquired using a Zeiss LSM800 confocal microscope equipped with a Plan-Apochromat 20×/0.8 M27 objective and controlled by ZEN 2.3 software. Fluorescence excitation was performed using diode lasers at 405, 488, and 561 nm. Images were analyzed using a custom CellProfiler pipeline (Stirling et al, 2021). In CellProfiler, the DAPI-stained DNA was used to segment the nuclei and measure the mean intensities of BrdU and RPA therein. The quantification of randomly selected 2000 nuclei per condition were plotted using R and RStudio.

### Flow cytometry

After the indicated treatments, the cells were dissociated with TrypLe Select, washed with PBS and fixed with ice-cold ethanol. For labeling the intracellular markers, the cells were permeabilized and blocked with 0.5% Triton X-100 and 5% FBS in PBS, and then incubated with primary antibodies, followed by fluorescently tagged secondary antibodies. For detecting BrdU incorporation, the samples were denatured with 2 N HCl and neutralized with citrate-phosphate buffer pH 7.4 prior to adding the anti-BrdU primary antibody. Finally, the DNA staining solution was added (10 μg/ml propidium iodide and 10 μg/ml RNase in PBS). The samples were analyzed with a CytoFLEX S flow cytometer, and the measurement was evaluated with Kaluza Analysis Software.

### DNA fiber assay (replication velocity)

P19 cells were treated with 500 μM hydroxyurea (HU) or 5 μM camphotecin (CPT) with additional treatment (PARGi, CHK1i and their combination; for more details, see experimental scheme in Figure legends) respectively for 180 min, then washed with PBS (prewarmed to 37 °C). Treated cells were pulse-labeled with 60 μM 5-chloro-2’-deoxyuridine (CldU) for 30 min and washed with PBS (prewarmed to 37 °C), followed by pulse-labeling with 338 μM 5-iodo-2’-deoxyuridine (IdU) with PARGi, HU/CPT, and their combination for 30 min. Labeled cells were washed with PBS, harvested by trypsinization, and resuspended in PBS to a concentration of 2.5 × 10^5^ cells/ml. 3 μl of this cell suspension was mixed with 8 μl of lysis buffer [200 mM Tris-HCl (pH 7.5), 50 mM EDTA, 0.5% (w/v) SDS] directly on a slide and incubated for 4.5 min, followed by tilting the slides to 30°, causing the drops to run down by gravity. The spreads were then air-dried and fixed with methanol/acetic acid (3:1) at RT for 20 min. DNA was denatured in 2.5 M HCl for 1 h at RT and later washed three times with PBS. Slides were blocked in blocking solution [2% (w/v) BSA, 0.1% (v/v) Tween 20 in 1xPBS] for 20 min and incubated with primary antibodies, rat monoclonal anti-BrdU (ab6326, Abcam; for detection of CldU) and mouse monoclonal anti-BrdU (347580, BD Biosciences, for detection of IdU) in blocking solution for 2 h at RT in a humid chamber. Afterward, the samples were washed three times in PBST [0.2% (v/v) Tween 20 in PBS] and incubated with secondary antibodies, anti-rat IgG Alexa 555 and anti-mouse IgG Alexa Fluor 488 in blocking solution for 1 h at RT in a humid chamber. The slides were washed in PBST and PBS, then dipped in water and mounted in Fluoromount-G. Images were acquired with a Leica DM6000 upright fluorescent microscope (63×/1.40 oil immersion). CldU and IdU tract lengths were measured using ImageJ and expressed as total fiber length in μm. At least 100 DNA fiber tracts were measured per each of three independent replicates. Statistical analysis was performed using the nonparametric Kruskal–Wallis test in GraphPad Prism 10.

### DNA fiber assay (restart and origin firing)

Cells were pulse-labeled with 20 μM thymidine analog 5-ethynyl-2’-deoxyuridine (EdU) for 20 min, then washed with PBS (prewarmed to 37 °C), followed by pulse-labeling with 84 μM 5-chloro-2’-deoxyuridine (CldU) for 20 min and washed with PBS (prewarmed to 37 °C). Pre-labeled cells were treated with HU with or without PARGi for 240 min (see Figure legends for details). Treated cells were washed with PBS (prewarmed to 37 °C), and pulse-labeled with 338 μM 5-iodo-2’-deoxyuridine (IdU) with or without additional treatment for 40 min. Labeled cells were washed with PBS, harvested by trypsinization, and resuspended in PBS to a concentration of 2.5 × 10^5^ cells/ml. About 3 μl of this cell suspension was mixed with 8 μl of lysis buffer [200 mM Tris-HCl (pH 7.5), 50 mM EDTA, 0.5% (w/v) SDS] directly on a slide and incubated for 4.5 min, followed by tilting the slides to 30°, causing the drops to run down by gravity. The spreads were then air-dried and fixed with methanol/acetic acid (3:1) at RT for 20 min. DNA was denatured in 2.5 M HCl for 1 h at RT and later washed three times with PBS. Incorporated EdU was labeled via click-chemistry with biotin-azide solution (100 mM Tris-HCl, pH 8.5, 2 mM CuSO_4_, 100 mM ascorbate, and 100 μM biotin-azide for 30 min, then washed three times in 1xPBS. Slides were blocked in blocking solution [2% (w/v) BSA, 0.1% (v/v) Tween 20 in 1xPBS] for 20 min and incubated with primary antibodies, rat monoclonal anti-BrdU (ab6326, Abcam, for detection of CldU), mouse monoclonal anti-BrdU (347580, BD Biosciences, for detection of IdU) and rabbit polyclonal anti-biotin (ab53494, Abcam, for detection of biotin-EdU), in blocking solution for 2 h at RT in a humid chamber. Afterward, the samples were washed three times in PBST [0.2% (v/v) Tween 20 in PBS] and incubated with secondary antibodies, goat anti-rat IgG Alexa 555, goat anti-mouse IgG Alexa Fluor 488, and donkey anti-rabbit IgG Alexa Fluor 647 in blocking solution for 1 h at RT in a humid chamber. The slides were washed in PBST and PBS, then dipped in water and mounted in Fluoromount-G. Images were acquired with a Leica DM6000 upright fluorescent microscope (63×/1.40 oil immersion). Replication restart was measured as the percentage of EdU-CldU tracts (stalled) relative to all EdU-CldU and EdU-CldU-IdU tracts (ongoing), using ImageJ. Origin firing was quantified as the percentage of IdU tracts relative to all EdU-CldU and EdU-CldU-IdU replication tracts. In both cases, at least 300 DNA replication tracts were quantified per independent replicate.

## Supplementary information

Peer Review File

Source data Fig. 1

Source data Fig. 2

Source data Fig. 3

Source data Fig. 4

Source data Fig. 5

Figure EV1 Source Data

Figure EV2 Source Data

Figure EV3 Source Data

Figure EV4 Source Data

Figure EV5 Source Data

Expanded View Figures

## Data Availability

This study includes no data deposited in external repositories. The source data of this paper are collected in the following database record: biostudies:S-SCDT-10_1038-S44319-026-00848-8.
