# Supplementary material for: PARG inhibition reduces ssDNA levels and limits RPA loading upon replication fork collapse
Source: EMBO Rep. 2026 Jul 4;27(16):4855–78. doi: 10.1038/s44319-026-00848-8 (PMC13503914; doi:10.1038/s44319-026-00848-8)
Supplement: Supplementary file 1 [file 44319_2026_848_MOESM12_ESM.pdf]

Expanded View Figures

Figure EV1. Differences in HU and CPT-induced early replication stress signaling.

(**A**, **B**) P19 cells were pre-treated with ATRi (5 μM), ATMi (5 μM), and DNA-PKi (5 μM) for 30 min after which HU (0.5 mM) (**A**) or CPT (5 μM) (**B**) were added for an additional 1 h and 30 min with or without PARGi (5 μM). Cells were then lysed and subjected to western blotting with the indicated antibodies. Representative blot from three biological replicates. Samples from the same experiment were run on parallel gels. The corresponding intra-gel Ponceau S staining used as a loading control for each blot are provided in the Source Data file. (**C**) P19 cells were treated with HU (0.5 mM) with or without PARGi (5 μM) for the indicated time points and subjected to cell fractionation to assess the levels of PARylation and RPA32 in cytoplasmic, nuclear or chromatin fraction by western blotting. Ponceau S staining was used as a loading control. Representative blot from three biological replicates. (**D**) P19 cells were treated with HU (0.5 mM), APH (10 μM), CPT (5 μM), and ETO (10 μM) for 1 h. BrdU was added for the last 20 min to show the nucleotide incorporation. The cells were then fixed and stained with anti-BrdU and propidium iodide (DNA content), and then the samples were analyzed with flow cytometry. FACS images of a representative from three biological replicates are shown. Source data are available online for this figure.

 © The Author(s)

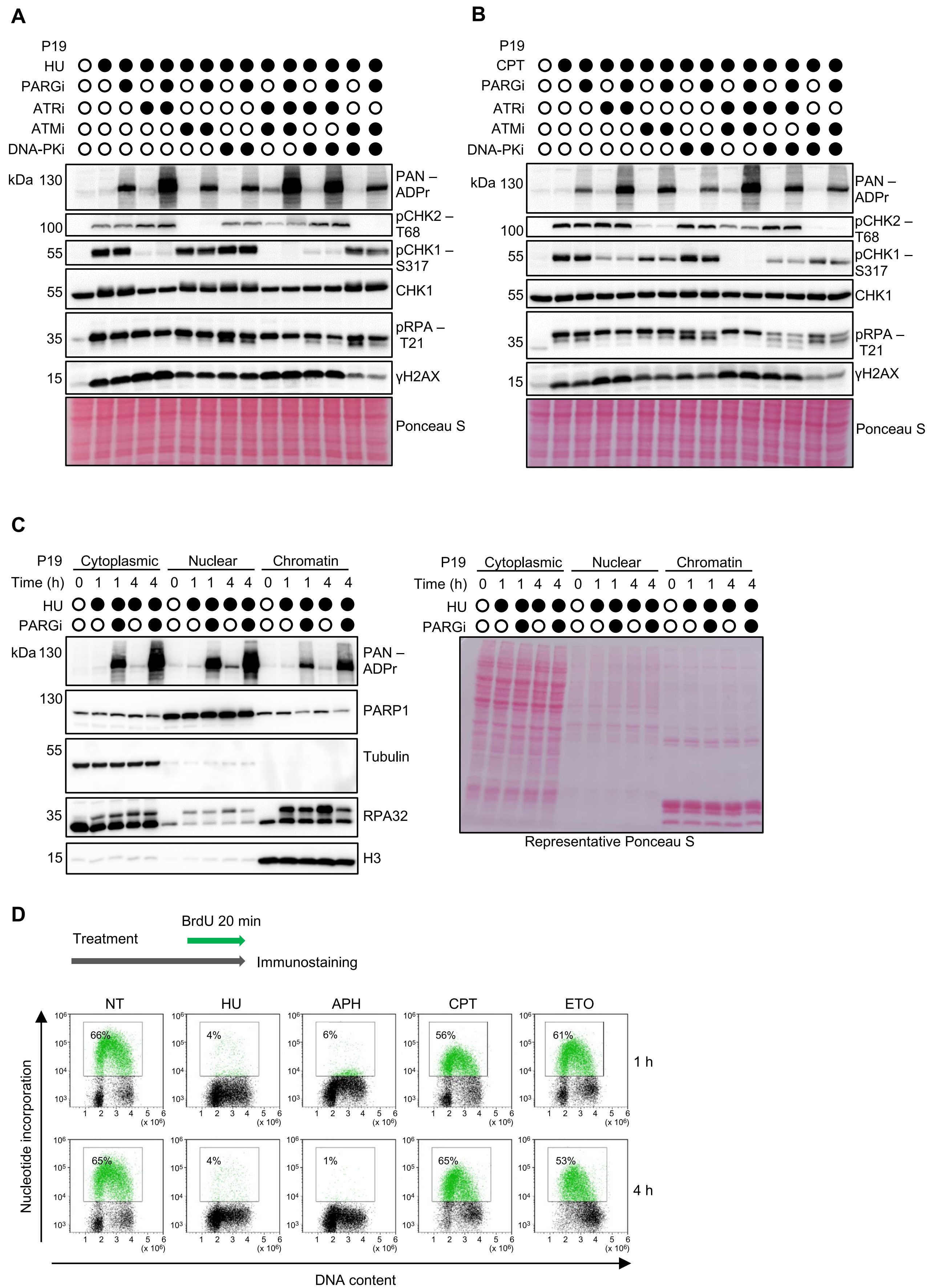
A
P19
HU
PARGi
ATRi
ATMi
DNA-PKi
kDa 130
PAN – ADPr
100
pCHK2 – T68
55
pCHK1 – S317
55
CHK1
35
pRPA – T21
15
γH2AX
Ponceau S
B
P19
CPT
PARGi
ATRi
ATMi
DNA-PKi
kDa 130
PAN – ADPr
100
pCHK2 – T68
55
pCHK1 – S317
55
CHK1
35
pRPA – T21
15
γH2AX
Ponceau S
C
P19
Cytoplasmic
Nuclear
Chromatin
Time (h) 0 1 1 4 4 0 1 1 4 4 0 1 1 4 4
HU
PARGi
kDa 130
PAN – ADPr
130
PARP1
55
Tubulin
35
RPA32
15
H3
Representative Ponceau S
D
Treatment
BrdU 20 min
Immunostaining
NT
HU
APH
CPT
ETO
66%
4%
6%
56%
61%
1 h
65%
4%
1%
65%
53%
4 h
Nucleotide incorporation
DNA content

© The Author(s)

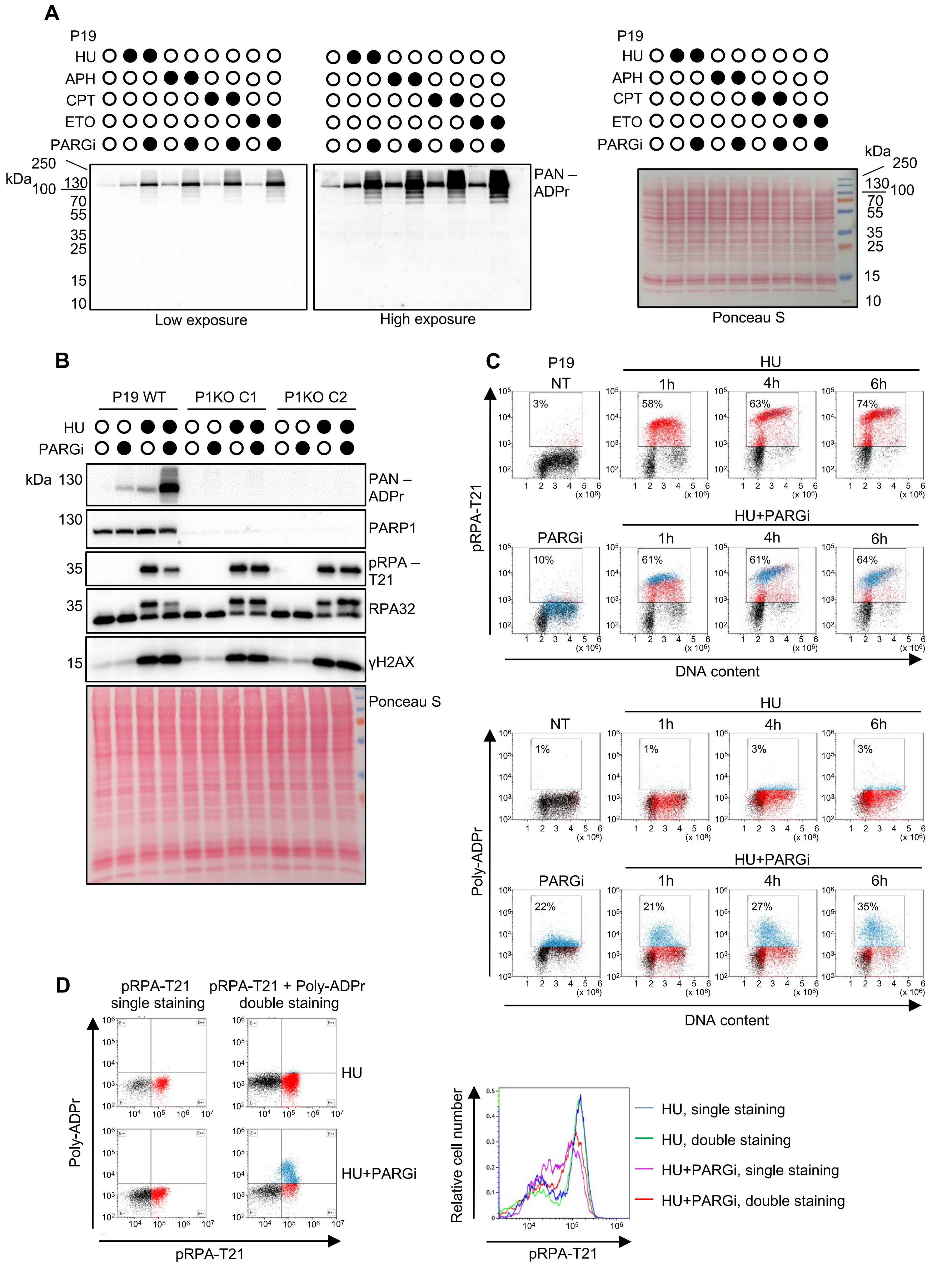
A
P19
HU
APH
CPT
ETO
PARGi
kDa
250
130
100
70
55
35
25
15
10
PAN – ADPr
Low exposure
High exposure
Ponceau S
B
P19 WT
P1KO C1
P1KO C2
PARP1
pRPA – T21
RPA32
γH2AX
C
NT
1h
4h
6h
HU+PARGi
pRPA-T21
Poly-ADPr
DNA content
D
pRPA-T21 single staining
pRPA-T21 + Poly-ADPr double staining
Relative cell number
HU, single staining
HU, double staining
HU+PARGi, single staining
HU+PARGi, double staining

 © The Author(s)

Figure EV2. Role of PARylation in pRPA reduction.

(**A**) P19 cells were treated with HU (0.5 mM), APH (10 μM), CPT (5 μM), and ETO (10 μM) with or without PARGi (5 μM) for 4 h. Cells were then lysed and subjected to western blotting with the PAN-ADPr detecting reagent (MABE1016). Ponceau S staining was used as a loading control. Representative blot from 2 biological replicates. (**B**) P19 WT and PARP1 KO cells (clones P1KO C1 and P1KO C2) were treated with PARGi (5 μM), HU (0.5 mM) and their combination for 7 h, then lysed, and samples were analyzed by western blotting with the indicated antibodies. Representative blot from three biological replicates. Samples from the same experiment were run on parallel gels. The corresponding intra-gel Ponceau S staining used as a loading control for each blot are provided in the Source Data file. (**C**) P19 cells were treated with HU (0.5 mM) with or without PARGi (5 μM) for the indicated time points, fixed and analyzed by FACS with the indicated antibodies. The pRPA-T21^{+} cells are colored in red, and PAR^{+} cells are blue throughout all of the FACS images. The images are from the same representative experiment shown in Fig. 1D, E. (**D**) P19 cells were treated with HU (0.5 mM) with or without PARGi (5 μM) for 4 h, then fixed, stained with either pRPA-T21 antibody alone or in combination with Poly-ADPr antibody, and then analyzed by FACS. Representative FACS images from 3 biological replicates are shown. Left: Dot plots of pRPA-T21 and Poly-ADPr fluorescence intensity show the inverse correlation of the signals. Right: Overlay histograms show that Poly-ADPr antibody does not interfere with the pRPA-T21 signal. Source data are available online for this figure.

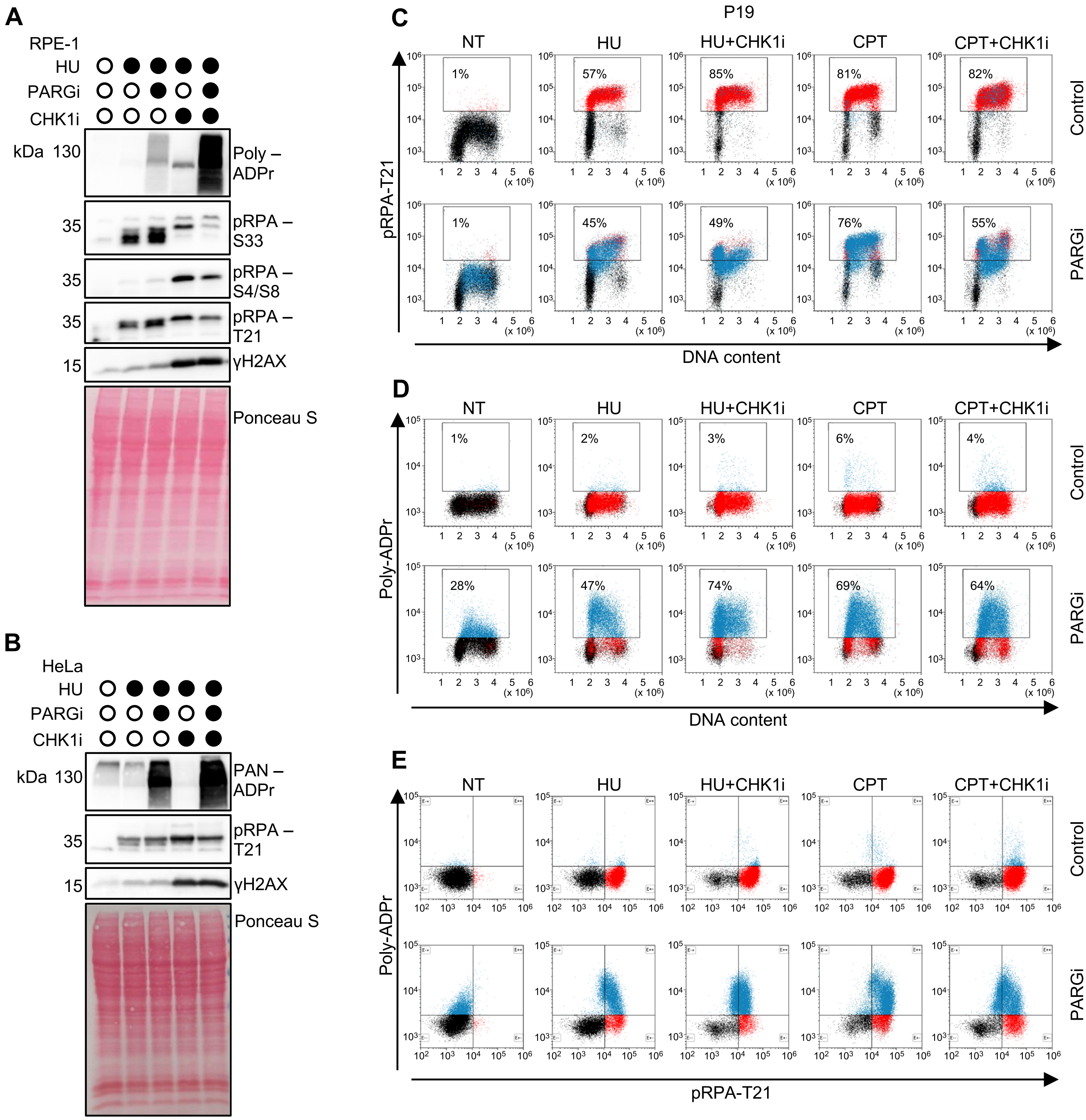

Figure EV3. CHK1 inhibition promotes PARGi-induced pRPA reduction under HU treatment in different cell lines.

(**A**) RPE-1 cells were treated with HU (1 mM) in the presence or absence of PARGi (5 μM) and CHKi (5 μM) for 24 h. Cells were lysed and samples analyzed by western blotting with the indicated antibodies. Representative blot from 3 biological replicates. Samples from the same experiment were run on parallel gels. The corresponding intra-gel Ponceau S staining used as a loading control for each blot are provided in the Source Data file. (**B**) HeLa cells were treated with HU (1 mM) in the presence or absence of PARGi (5 μM) and CHKi (5 μM) for 24 h. Cells were lysed, and the samples were subjected to western blotting with the indicated antibodies. Ponceau S staining was used as a loading control. Representative blot from 3 biological replicates. (**C**, **D**, **E**) P19 cells treated with HU (0.5 mM) and CTP (5 μM) with or without PARGi (5 μM) in the presence or absence of CHK1i (5 μM) for 4 h, then fixed, stained and analyzed by FACS. FACS images of a representative from three biological replicates are shown. Source data are available online for this figure.

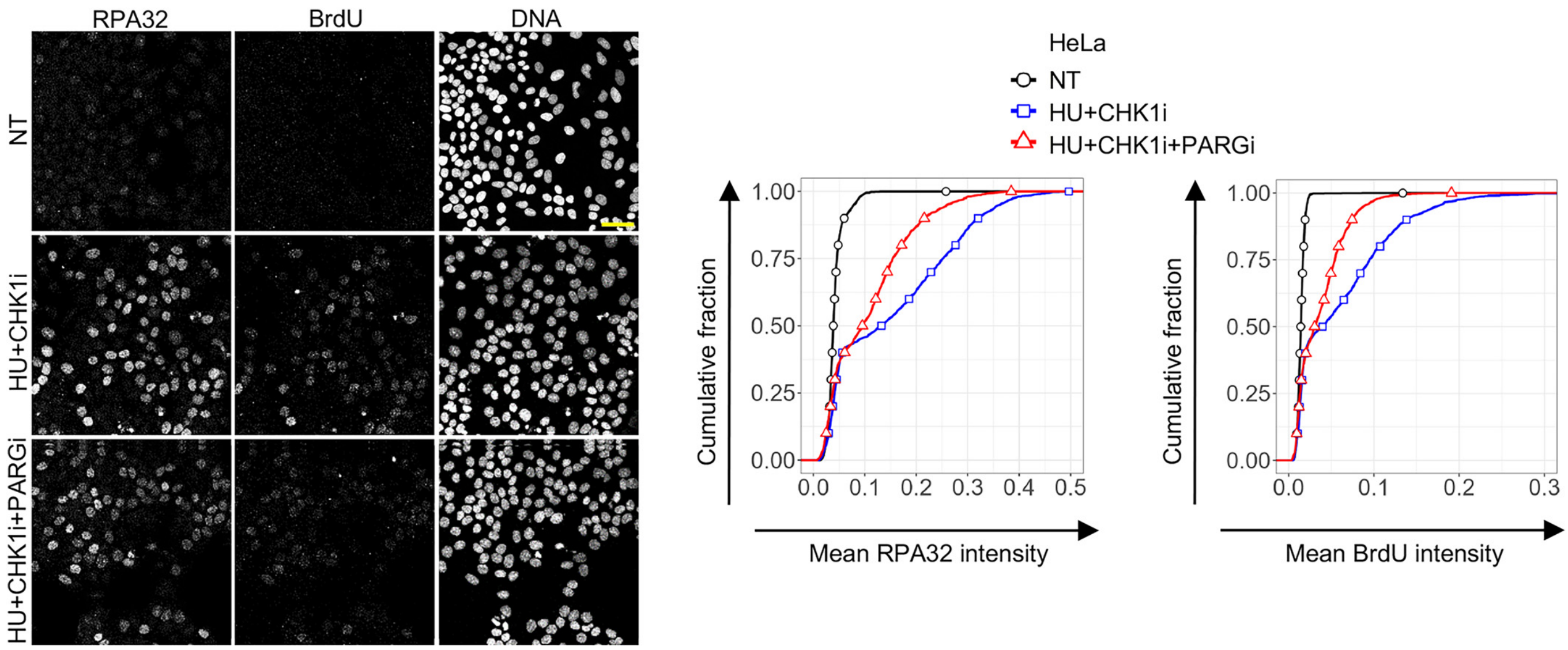

Figure EV4. Correlation of chromatin-bound RPA32 and ssDNA.

HeLa cells were labeled with BrdU (10 µM) for 48 h and then treated with HU (1 mM) and CHK1i (5 µM) in the presence or absence of PARGi (5 µM) for 24 h. ssDNA was detected by BrdU and RPA32 immunofluorescence. Scale bar is 50 µm. Representative staining from three independent experiments. The quantification of randomly selected 2000 nuclei per condition were plotted. Source data are available online for this figure.

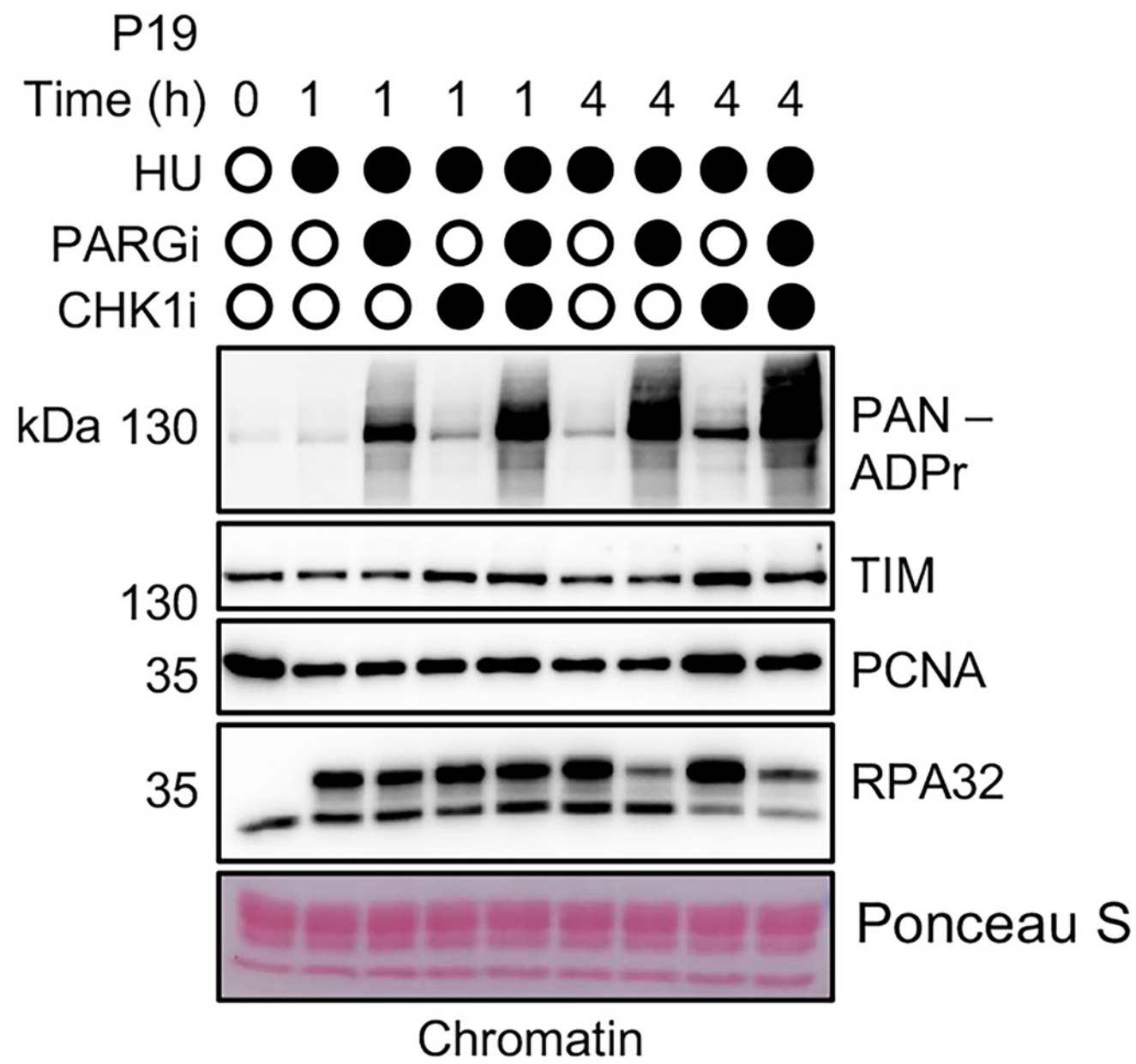

Figure EV5. CHK1 inhibition increases the level of chromatin-bound TIM and PCNA.

P19 cells were treated with HU (0.5 mM) with or without PARGi (5 μM) in the presence or absence of CHK1i (5 μM) for 1 or 4 h and subjected to chromatin fractionation. PARylation, TIM, PCNA, and RPA32 levels were assessed by western blotting. Representative blot from three biological replicates. Samples from the same experiment were run on parallel gels. The corresponding intra-gel Ponceau S staining used as a loading control for each blot are provided in the Source Data file. Source data are available online for this figure.

 © The Author(s)
