# Supplementary material for: PARG inhibition reduces ssDNA levels and limits RPA loading upon replication fork collapse
Source: EMBO Rep. 2026 Jul 4;27(16):4855–78. doi: 10.1038/s44319-026-00848-8 (PMC13503914; doi:10.1038/s44319-026-00848-8)
Supplement: Supplementary file 2 [file 44319_2026_848_MOESM1_ESM.pdf]

EMBO reports

PARG inhibition reduces ssDNA levels and limits RPA loading upon replication fork collapse

Alexandra Mihuţ, Debanjan Ghosh, Adrián Kószó, Dávid Szüts, Sarka Salajkova, Martin Andrs, Jana Dobrovolna, Roberta Fajka-Boja, and Gyula Timinszky

Corresponding author(s): Gyula Timinszky (timinszky.gyula@brc.hu) , Roberta Fajka-Boja (fajka_boja.roberta@brc.hu)

Review Timeline:

Submission Date:	21st Oct 25
Editorial Decision:	27th Nov 25
Revision Received:	1st May 26
Editorial Decision:	21st May 26
Revision Received:	5th Jun 26
Accepted:	12th Jun 26

Editor: Achim Breiling

Transaction Report:

(Note: With the exception of the correction of typographical or spelling errors that could be a source of ambiguity, letters and reports are not edited. Depending on transfer agreements, referee reports obtained elsewhere may or may not be included in this compilation. Referee reports are anonymous unless the Referee chooses to sign their reports.)

1st Editorial Decision **27th Nov 2025**

Dear Dr. Timinszky,

Thank you for the submission of your manuscript to EMBO reports. I have now received reports from the three referees that were asked to evaluate your study, which can be found at the end of this email.

As you will see, the referees think that these findings are of interest. However, they have several comments, concerns, and suggestions, indicating that a major revision of the manuscript is necessary to allow publication of the study in EMBO reports. As the reports are below, and all the referee concerns need to be addressed, I will not detail them here.

Given the constructive referee comments, I would thus like to invite you to revise your manuscript with the understanding that the concerns of the referees must be addressed in the revised manuscript and/or in a detailed point-by-point response. Acceptance of your manuscript will depend on a positive outcome of a second round of review. It is EMBO reports policy to allow a single round of revision only and acceptance of the manuscript will therefore depend on the completeness of your responses included in the next, final version of the manuscript.

Revised manuscripts should be submitted within three months of a request for revision. Please contact me to discuss the revision (also by video chat) if you have questions or comments regarding the revision, or should you need additional time.

When submitting your revised manuscript, please also carefully review the instructions that follow below.

PLEASE NOTE THAT upon resubmission revised manuscripts are subjected to an initial quality control prior to exposition to re-review. Upon failure in the initial quality control, the manuscripts are sent back to the authors, which may lead to delays. Frequent reasons for such a failure are the lack of the data availability section (please see below) and the presence of statistics based on n=2 (the authors are then asked to present scatter plots or provide more data points).

When submitting your revised manuscript, we will require:

1) a .docx formatted version of the final manuscript text (including legends for main figures, EV figures and tables), but without the figures included. Figure legends should be compiled at the end of the manuscript text.

2) individual production quality figure files as .eps, .tif, .jpg (one file per figure), of main figures and EV figures. Please upload these as separate, individual files upon re-submission.

The Expanded View format, which will be displayed in the main HTML of the paper in a collapsible format, has replaced the Supplementary information. You can submit up to 6 images as Expanded View. Please follow the nomenclature Figure EV1, Figure EV2 etc. The figure legend for these should be included in the main manuscript document file in a section called Expanded View Figure Legends after the main Figure Legends section. Additional Supplementary material should be supplied as a single pdf file labeled Appendix. The Appendix should have page numbers and needs to include a table of content on the first page (with page numbers) and legends for all content. Please follow the nomenclature Appendix Figure Sx, Appendix Table Sx etc. throughout the text, and also label the figures and tables according to this nomenclature.

For more details, please refer to our guide to authors:
http://www.embopress.org/page/journal/14693178/authorguide#manuscriptpreparation

Please consult our guide for figure preparation:
http://wol-prod-cdn.literatumonline.com/pb-assets/embo-site/EMBOPress_Figure_Guidelines_061115-1561436025777.pdf

See also the guidelines for figure legend preparation:
https://www.embopress.org/page/journal/14693178/authorguide#figureformat

3) a .docx formatted letter INCLUDING the reviewers' reports and your detailed point-by-point responses to their comments. As part of the EMBO Press transparent editorial process, the point-by-point response is part of the Review Process File (RPF), which will be published alongside your paper.

4) a complete author checklist, which you can download from our author guidelines (https://www.embopress.org/page/journal/14693178/authorguide). Please insert page numbers in the checklist to indicate where the requested information can be found in the manuscript. The completed author checklist will also be part of the RPF.

Please also follow our guidelines for the use of living organisms, and the respective reporting guidelines:
http://www.embopress.org/page/journal/14693178/authorguide#livingorganisms

5) that primary datasets produced in this study (e.g. RNA-seq, ChIP-seq, structural and array data) are deposited in an

appropriate public database. If no primary datasets have been deposited, please also state this in a dedicated section (e.g. 'No primary datasets have been generated and deposited'), see below.

See also: http://embor.embopress.org/authorguide#datadeposition

Please remember to provide a reviewer password if the datasets are not yet public.

The accession numbers and database should be listed in a formal "Data Availability" section that follows the model below. This is now mandatory (like the COI statement). Please note that the Data Availability Section is restricted to new primary data that are part of this study. This section is mandatory. As indicated above, if no primary datasets have been deposited, please state this in this section

Data availability

The datasets produced in this study are available in the following databases:

- RNA-Seq data: Gene Expression Omnibus GSE46843 (https://www.ncbi.nlm.nih.gov/geo/query/acc.cgi?acc=GSE46843)
- [data type]: [name of the resource] [accession number/identifier/doi] ([URL or identifiers.org/DATABASE:ACCESSION])

*** Note - All links should resolve to a page where the data can be accessed. ***

Moreover, I have these editorial requests:

6) We now request the publication of original source data with the aim of making primary data more accessible and transparent to the reader. You will receive a separate email with instructions for providing source data with your revised manuscript, including information how to upload and organize the files.

7) Our journal encourages inclusion of *data citations in the reference list* to directly cite datasets that were re-used and obtained from public databases. Data citations in the article text are distinct from normal bibliographical citations and should directly link to the database records from which the data can be accessed. In the main text, data citations are formatted as follows: "Data ref: Smith et al, 2001" or "Data ref: NCBI Sequence Read Archive PRJNA342805, 2017". In the Reference list, data citations must be labeled with "[DATASET]". A data reference must provide the database name, accession number/identifiers and a resolvable link to the landing page from which the data can be accessed at the end of the reference. Further instructions are available at: http://www.embopress.org/page/journal/14693178/authorguide#referencesformat

8) Regarding data quantification and statistics, please make sure that the number "n" for how many independent experiments were performed, their nature (biological versus technical replicates), the bars and error bars (e.g. SEM, SD) and the test used to calculate p-values is indicated in the respective figure legends (also for EV and Appendix figures). Please also check that all the p-values are explained in the legend, and that these fit to those shown in the figure. Please provide statistical testing where applicable. Please avoid the phrase 'independent experiment' but clearly state if these were biological or technical replicates. Please also indicate (e.g. with n.s.) if testing was performed, but the differences are not significant. In case n=2, please show the data as separate datapoints without error bars and statistics. See also: http://www.embopress.org/page/journal/14693178/authorguide#statisticalanalysis

If n<5, please show single datapoints for diagrams.

9) Please confirm that for all Western blot panels (main and EV figures) the loading control was run on the same gel as the other proteins detected. Please note that we discourage comparisons between samples on different gels/blots, even if the samples derive from one experiment, as confounding factors reduce comparability. If unavoidable, the figure legend must state that the samples derive from the same experiment and that gels/blots were processed in parallel. If a 'representative' loading control is shown for multiple gels/blots, the intra-gel controls should be shown in the source data files, and the figure legends should describe the data displayed accurately. See our author guidelines:

https://www.embopress.org/page/journal/14693178/authorguide#datapresentationformat (section 'Electrophoretic gels and blots').

and

https://www.embopress.org/image-integrity

10) Please also note our reference format: http://www.embopress.org/page/journal/14693178/authorguide#referencesformat

11) We updated our journal's competing interests policy in January 2022 and request authors to consider both actual and

perceived competing interests. Please review the policy https://www.embopress.org/competing-interests and update your competing interests if necessary. Please name this section 'Disclosure and Competing Interests Statement' and put it after the Acknowledgements section.

12) We now use CRediT to specify the contributions of each author in the journal submission system. CRediT replaces the author contribution section. Please use the free text box to provide more detailed descriptions and do NOT provide your final manuscript text file with an author contributions section. See also our guide to authors:
https://www.embopress.org/page/journal/14693178/authorguide#authorshipguidelines

13) All Materials and Methods need to be described in the main text using our 'Structured Methods' format, which is required for all research articles. According to this format, the Methods section should include a Reagents and Tools Table (listing key reagents, experimental models, software, and relevant equipment and including their sources and relevant identifiers), uploaded as separate file, and a Methods section in which we encourage the authors to describe their methods using a step-by-step protocol format with bullet points, to facilitate the adoption of the methodologies across labs. More information on how to adhere to this format as well as downloadable templates (.doc) for the Reagents and Tools Table can be found in our author guidelines (section 'Structured Methods'):

https://www.embopress.org/page/journal/14693178/authorguide#manuscriptpreparation

14) Please order the manuscript sections like this, using only these names:
Title page - Abstract (max. 175 words) - Keywords - Introduction - Results - Discussion - Methods - Data availability section - Acknowledgements (please include here all the funding information) - Disclosure and Competing Interests Statement - References - Figure legends - Expanded View Figure legends

Please also provide a title with not more than 100 characters (including spaces).

15) Please make sure that all the funding information is also entered into the online submission system and that it is complete and similar to the one in the acknowledgement section of the manuscript text file.

We would also welcome the submission of cover suggestions, or motifs to be used by our Graphics Illustrator in designing a cover.

As part of the EMBO publication's Transparent Editorial Process, EMBO reports publishes online a Review Process File (RPF) to accompany accepted manuscripts. This File will be published in conjunction with your paper and will include the referee reports, your point-by-point response and all pertinent correspondence relating to the manuscript.

You are able to opt out of this by letting the editorial office know (emboreports@embo.org). If you do opt out, the Review Process File link will point to the following statement: "No Review Process File is available with this article, as the authors have chosen not to make the review process public in this case."

I look forward to seeing a revised form of your manuscript when it is ready.

Yours sincerely,

Achim Breiling
Senior Editor
EMBO Reports

Referee #1:

Poly-ADP-ribosylation (PAR) is a biologically significant posttranslational modification, given its relevance in clinical contexts. Inhibitors of its writer, PARPs, have already been used in the treatment of homologous-recombination-deficient cancers, and targeting its eraser, PARG, has also been proposed as a potential cancer therapy. Sustained PARylation resulting from PARG inhibition exacerbates replication defects, which are likely responsible for its cytotoxic effects. The authors investigate the mechanisms by which this toxic state is induced in cellular models, employing various assays with different replication stress-inducing agents. They focus particularly on the regulation of RPA at stressed replication forks, induced by hydroxyurea or camptothecin (CPT), during PARG inhibition. Their findings demonstrate that stabilized PAR chains become toxic during replication by reducing chromatin-bound RPA, which leads to diminished ATR signaling and subsequent fork collapse. The primary model used is the embryonic P19 cell line, which the authors extensively characterize as highly sensitive to hydroxyurea treatment.

I find this manuscript suitable for publication in EMBO Reports with minor revisions. It presents a clear and focused key message: that sustained PARylation attenuates ATR-CHK1 signaling upon replication fork collapse by limiting RPA loading. Although the connection between reduced RPA loading upon PARG inhibition has been previously reported-albeit with some controversy in the recent literature-the authors' demonstration that this modulation impacts the ATR-CHK1 pathway represents a novel contribution to the field.

The experimental data are robust and support the central conclusion. Minor additional experiments or points of clarification would further strengthen the manuscript.

General points:

- The authors need to discuss much more strongly and extensively what is the advance of their findings compared to Illuzzi et al 2014, which described that excessive PARylation in PARG-depleted cells impairs RPA2 association with the replication fork. Clearly there have been discrepancies in the literature on the effect of PARG inhibition or depletion on RPA functions, therefore, it is valuable to address the issue again, also in an embryonic cell line, however, it should be more extensively discussed what is the overlap of the findings between the previous article and what novelty this present one brings (for example the feedback loop between PARGi and ATR signaling). This would significantly strengthen the manuscript.

- The authors use the pan-ADPr antibody throughout the manuscript but focus on PARylation at the height of PARP1. It would be interesting to see how for example histone PARylation changes in the combination of HU+PARGi. Including the full blots (at least in the supplementary) and commenting on the different, clearly-recognizable PARylated species on it would give even more biological insight into PARylation at the replication forks. Including a blot with a PAR-specific antibody at least for the first figure would also strengthen the point that the observed effects are through PAR and not mono-ADPr.

- The figure legends do not clearly specify the number of independent experiments (n). Please indicate the number of replicates for each experiment.

Minor points:

- In Figure 1, the levels of total CHK1 appear to increase after 1 hour of HU treatment. This complicates the interpretation of the abundance of the phosphorylated species. The observed increase could potentially result from poor separation of the higher migrating p-CHK1 species; however, this is not apparent at other time points (4 hours), where pS317 levels remain relatively high. The authors should address this by either providing a better-resolved image or by normalizing the phosphorylated signal to total CHK1 levels across three independent experiments, with quantification included in the supplementary data.

- Quantification of the nuclear intensity of the pRPA signal between the different conditions in the immunofluorescence experiments- maybe even correlating them with PAR intensity- would be an important addition next to the current visual display. Here as well, the number of nuclei quantified should be clearly stated.

- Figure 3 A is lacking cytoplasmic/nuclear controls. While the chromatin fractionation was clearly successful, it is puzzling to see more (phospho and total) RPA32 and ADP-ribosylated PARP1 in the cytoplasm than in the nuclear fraction.

- The first paragraph of the introduction contains long and complicated sentences, please rephrase for better readability.

Referee #2:

Mihut et al. present PARGi-dependent changes of RPA phosphorylation and its chromatin binding upon induced replication stress. Specifically, they show in P19 mouse teratocarcinoma cells that hydroxyurea-induced phosphorylation of RPA32 at T21 and S4/S8 is reduced after PARGi and propose a feedback loop between declining ATR activity after replication fork collapse, enhanced PARylation, RPA release from DNA, and a further reduction in ATR signaling. Although potentially interesting, the proposed feedback loop is not sufficiently substantiated, the relevance of the conditions in which it may occur is unclear, and the observations seem cell line-specific, putting the generalizability into question.

Main points:

1) The PARGi-induced reduction in pRPA T21 and pS4/S8 and its correlation with reduced ATR signaling (pCHK1) is only observed in a single carcinoma cell line. The relevance and generalizability of this observation thus remain unclear.
2) The reduction in pRPA T21 and pS4/S8 and its correlation with reduced ATR signaling (pCHK1) is only observed upon PAR stabilization through PARGi, in presence of HU or APH. Whether physiological PARylation upon fork stalling and collapse would ever affect RPA without PARGi remains unclear. The combination of HU/APH with PARGi seems quite artificial and it is difficult to imagine conditions where cells would be faced with such a challenge.
3) In some of the assays the effect seems quite moderate. For instance, the differences in pRPA T21 plus/minus PARGi in

Figure 1E are small. In Figure 1G, please show the HU-only conditions for comparison. In Figure 2C, second and third lane, hardly any difference in pRPA can be seen. For the IF and FACS experiments, in which pRPA T21 and poly-ADPr were detected simultaneously, how can the authors be sure that there is no steric competition between the antibodies, which could explain reduced pRPA T21 signals in cells with high poly-ADPr signals?
4) Many of the results are not particularly novel or unexpected, e.g. that HU (fork stalling and uncoupling) and CPT at the dose used (mainly DSBs) reflect different outcomes and induce different signaling (ATR-CHK1 at stalled forks, ATM-CHK2 at DSBs), and that replication stress combined with checkpoint inhibition promotes fork collapse and PARP1 activation.
5) On the other hand, the mechanism how persistent PAR chains under conditions of replication stress plus PARGi limit RPA loading on chromatin remains unclear, and indirect effects (e.g. loss of ssDNA due to massive DNA damage, NAD+/ATP depletion due to stabilized PAR chains) are possible.
6) For a more complete picture of replication dynamics in the conditions studied, DNA fiber or DNA combing assays to look at replication fork speed, fork remodeling, ssDNA gaps, and origin firing would be needed.
7) Information on replicate experiments and reproducibility seems missing.
8) Overall, the manuscript is rather observational, descriptive and the text difficult to follow. Some of the sections read as long lists of observations and the coherence could be improved.

Minor points:

1) Introduction, line 4, is the proofreading function of the polymerase relevant for helicase-polymerase uncoupling?
2) Introduction, line 13, DNA replication seems unlikely to resume after replication catastrophe, and the sentence ("In such cases, ...") is misleading.
3) PARylation was previously linked to CHK1 retention at replication forks and its activation (PMID: 24356582). How do the authors reconcile this with their own data? This should be discussed.
4) Studies from Caldecott and Hanzlikova, using PARGi to reveal PARylation in the context of replication (PMIDs: 29983321, 35332322) should also be discussed.

Referee #3:

This is a very interesting manuscript examining the inter-relationship between PARylation and ATR activation. In a way, both PARylation and ATR activation can be considered as alarm systems (checkpoints) for various forms of DNA damage and DNA replication stress. In this regard, cells may have feedback systems, such as when one pathway is activated, the other is suppressed. This manuscript describes such a feedback mechanism. Overall, I find that this manuscript is timely and should be considered for publication, once the following points below are addressed.

Specific Points:

1. Some statements in the Introduction seem self-conflicting. For example, the sentences: "ATR checkpoint failure during replication stress leads to fork collapse. Concomitantly, RPA exhaustion due to uncontrolled origin firing, deregulated fork remodeling, fork component disintegration, to name a few, will generate unrepaired DNA breaks at the fork and collapse it", are followed by: "In such cases, replication can resume through activation of a nearby dormant origin or via fork restart mechanisms that stabilize and reactivate the stalled fork". How did a collapsed fork (first two sentences), become a stalled fork (third sentence)? A stalled fork is usually thought to be a fork that has not yet collapsed.

2. Fig. 1A shows that a PARG inhibitor (PARGi) combined with HU treatment, decreased the levels of CHK1 and RPA phosphorylation (pCHK1 ad pRPA) induced by HU alone. This effect was seen only at later time points. Fig. 1B and 1C confirm this observation and further show that, in the absence of PARP1 or when PARP1 is inhibited, the PARGi does not decrease the levels of pRPA in HU-treated cells. Hence, high levels of PARylation are important for the decrease in pRPA (and pCHK1) levels driven by PARGi in HU-treated cells.

3. Fig. 2 extends the observations of Fig. 1. Treatment of cells with aphidicolin (Aph) and PARGi showed similar effects as treatment with HU and PARGi, indicating that the agent that induces stalling of DNA replication is not important. The easiest interpretation of these findings is that PARGi prevents processing of stalled/collapsed forks, such that there is less DNA end resection, less single-stranded DNA (ssDNA) and less activation of ATR. The results of Fig. 3 support this interpretation, as chromatin bound RPA levels decreased by PARGi in cells treated with HU.

4. Fig. 4 provides data indicating that inhibiting ATR in HU-treated cells leads to firing of new origins, which is consistent with previous results.

5. In the Discussion, the authors provide various models why PARGi reduce the levels of chromatin-bound RPA in HU-treated cells, but do not consider the model described in point 3 above. There are methods to monitor the presence of ssDNA, for example treating the cells with BrdU and then monitoring the presence of ssDNA by immunofluorescence under non-denaturing conditions. Perhaps, the authors could try such an experiment, as this could help distinguish between the model in point 3 above and the models cited by the authors in the Discussion.

Point by point response to the comments of the referees for the manuscript "*Sustained poly(ADP-ribosyl)ation limits RPA loading and attenuates ATR–CHK1 signaling upon replication fork collapse*" by Mihut et al.

We thank the reviewers for their careful reading of our manuscript and their most useful comments to improve our study. We carried out additional experiments to address the raised questions. Below is our point-by-point response (in blue) to the reviewers' comments (shown in black).

Point-by-point response

Referee #1:

Poly-ADP-ribosylation (PAR) is a biologically significant posttranslational modification, given its relevance in clinical contexts. Inhibitors of its writer, PARPs, have already been used in the treatment of homologous-recombination-deficient cancers, and targeting its eraser, PARG, has also been proposed as a potential cancer therapy. Sustained PARylation resulting from PARG inhibition exacerbates replication defects, which are likely responsible for its cytotoxic effects. The authors investigate the mechanisms by which this toxic state is induced in cellular models, employing various assays with different replication stress-inducing agents. They focus particularly on the regulation of RPA at stressed replication forks, induced by hydroxyurea or camptothecin (CPT), during PARG inhibition. Their findings demonstrate that stabilized PAR chains become toxic during replication by reducing chromatin-bound RPA, which leads to diminished ATR signaling and subsequent fork collapse. The primary model used is the embryonic P19 cell line, which the authors extensively characterize as highly sensitive to hydroxyurea treatment.

I find this manuscript suitable for publication in EMBO Reports with minor revisions. It presents a clear and focused key message: that sustained PARylation attenuates ATR-CHK1 signaling upon replication fork collapse by limiting RPA loading. Although the connection between reduced RPA loading upon PARG inhibition has been previously reported-albeit with some controversy in the recent literature-the authors' demonstration that this modulation impacts the ATR-CHK1 pathway represents a novel contribution to the field.

The experimental data are robust and support the central conclusion. Minor additional experiments or points of clarification would further strengthen the manuscript.

General points:

- The authors need to discuss much more strongly and extensively what is the advance of their findings compared to Illuzzi et al 2014, which described that excessive PARylation in PARG-depleted cells impairs RPA2 association with the replication fork. Clearly there have been discrepancies in the literature on the effect of PARG inhibition or depletion on RPA functions, therefore, it is valuable to address the issue again, also in an embryonic cell line, however, it should be more extensively discussed what is the overlap of the findings between the previous article and what novelty this present one brings (for example the feedback loop between PARGi and ATR signaling). This would significantly strengthen the manuscript.

We thank the reviewer for this insightful comment and agree that the relationship of our findings to Illuzzi et al. (2014) should be more clearly articulated. Accordingly, we revised the Introduction and Discussion to more explicitly acknowledge the important overlap between the

studies: both support the conclusion that defective PAR turnover during prolonged replication stress can lead to excessive PAR accumulation associated with reduced RPA loading.

We also clarify several advances of the present study. First, our data link reduced chromatin-bound RPA with attenuation of ATR–CHK1 signaling under PARGi-sensitive conditions. Second, by comparing multiple stressors, we show that the phenotype is strongly context dependent, being most pronounced under HU- and aphidicolin-induced fork stalling. Third, comparison of P19 and RPE-1 cells indicates that fork protection and restart capacity strongly influence susceptibility to this state. In addition, native BrdU analyses support reduced ssDNA exposure under conditions where RPA is diminished.

We further note that Illuzzi et al. primarily used PARG depletion, whereas our study focuses on acute pharmacological PARG inhibition, providing a complementary approach to examine PAR turnover during replication stress.

- The authors use the pan-ADPr antibody throughout the manuscript but focus on PARylation at the height of PARP1. It would be interesting to see how for example histone PARylation changes in the combination of HU+PARGi. Including the full blots (at least in the supplementary) and commenting on the different, clearly-recognizable PARylated species on it would give even more biological insight into PARylation at the replication forks. Including a blot with a PAR-specific antibody at least for the first figure would also strengthen the point that the observed effects are through PAR and not mono-ADPr.

We thank the reviewer for this suggestion. To distinguish PARylation from mono-ADP-ribosylation, we have used the 10H antibody primarily recongizing branched ADP-ribose chains (e.g. **Fig. 1C**). In addition, we now provide the corresponding full pan-ADPr immunoblots (**Fig. EV2A**) to improve transparency and to allow assessment of the major ADP-ribosylated species. These blots show prominent high-molecular-weight signals consistent with PARP1/PARP2-associated species under replication stress plus PARG inhibition. By contrast, we did not detect an increase in lower-molecular-weight signals that would suggest substantial histone ADP-ribosylation under these conditions.

- The figure legends do not clearly specify the number of independent experiments (n). Please indicate the number of replicates for each experiment.

We thank the reviewer for highlighting this important point. We have revised the figure legends throughout the manuscript to explicitly indicate the number of independent biological replicates for each experiment. Where applicable, the legends also now specify the quantification approach and statistical analyses used.

Minor points:
- In Figure 1, the levels of total CHK1 appear to increase after 1 hour of HU treatment. This complicates the interpretation of the abundance of the phosphorylated species. The observed increase could potentially result from poor separation of the higher migrating p-CHK1 species; however, this is not apparent at other time points (4 hours), where pS317 levels remain relatively high. The authors should address this by either providing a better-resolved image or by normalizing the phosphorylated signal to total CHK1 levels across three independent experiments, with quantification included in the supplementary data.

We agree that phosphorylation-dependent mobility shifts of CHK1 can complicate interpretation of the total CHK1 band pattern after HU treatment. To directly examine this possibility, we treated lysates with alkaline phosphatase prior to immunoblotting. Phosphatase treatment collapsed the slower-migrating CHK1 species and restored comparable migration between HU-treated and untreated samples, indicating that the apparent increase/broadening of the total CHK1 signal primarily reflects phosphorylation-dependent mobility changes rather

than a substantial increase in total CHK1 abundance. As these control experiments do not alter the main conclusions of the study, we present them here in the rebuttal rather than adding an additional supplementary figure (**Rebuttal Fig. R1**). Thus, the total CHK1 band itself is affected by phosphorylation-dependent migration and band broadening, making it an unreliable denominator for quantitative normalization. Such normalization could therefore be misleading rather than corrective. The PARGi induced changes in CHK1 phosphorylation were reproducible across independent experiments and the interpretation of the phospho-CHK1 response in the manuscript does not rely on an apparent increase in total CHK1 abundance, but on the treatment-dependent changes in the phosphorylated CHK1 species together with the accompanying RPA, γH2AX, and PARylation readouts. Ponceau S staining was used for loading control.

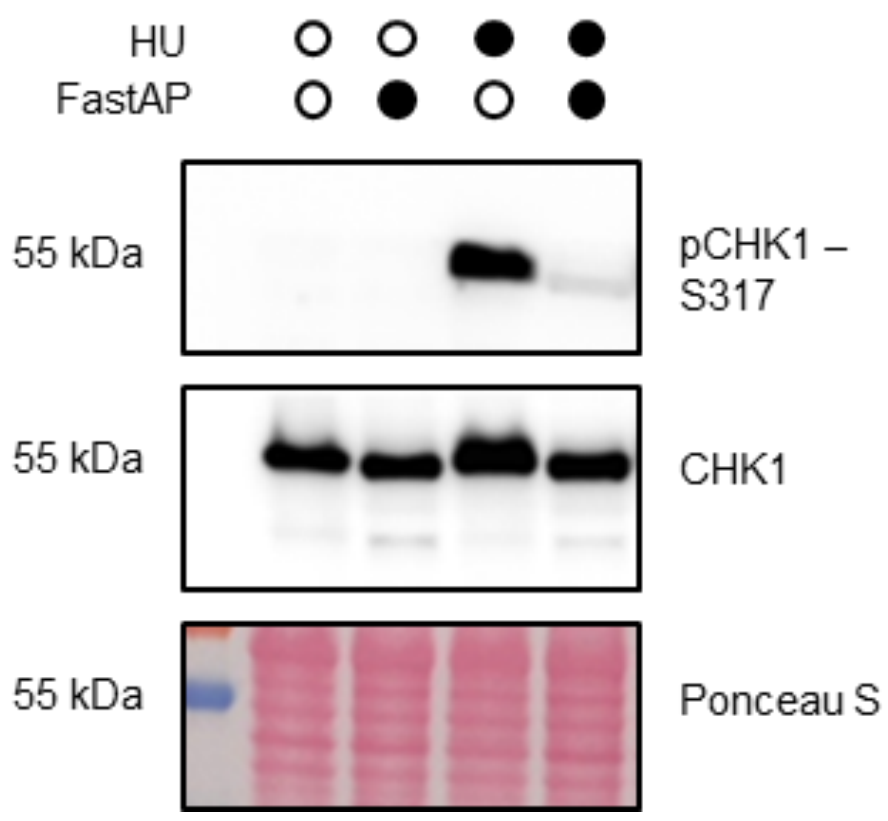

Figure R1: Testing the mobility of CHK1 and its phosphorylated form. P19 cells were treated with HU (0.5 mM) for 30 min to induce CHK1 phosphorylation or left untreated. Cells were then lysed and 1 U FastAP (alkaline phosphatase) was added to the indicated samples for 10 min at 37°C. The samples were then subjected to western blotting with the indicated antibodies.

- Quantification of the nuclear intensity of the pRPA signal between the different conditions in the immunofluorescence experiments- maybe even correlating them with PAR intensity- would be an important addition next to the current visual display. Here as well, the number of nuclei quantified should be clearly stated.

We expanded the analysis of the immunofluorescence data by quantifying mean nuclear pRPA signal intensities across the indicated conditions rather than relying solely on representative images. Mean nuclear pRPA intensities from 2000 nuclei per condition were plotted as cumulative fractions to show the differences between treatments (**Fig. 3D**). These analyses confirm that PARG inhibition reproducibly reduces the nuclear pRPA signal under the indicated replication stress conditions. The corresponding methodology and figure legends have been revised to clearly indicate the number of nuclei quantified and the number of independent experiments.

Regarding the suggested correlation with PAR intensity, the inverse relationship between PARylation and pRPA is addressed using flow cytometry, which provides a robust single-cell quantitative readout across large cell populations. These analyses show that pRPA-T21 intensity is reduced in PAR-positive/high-PAR cells during HU and PARGi co-treatment, supporting an inverse relationship between sustained PARylation and pRPA signal. In contrast, the immunofluorescence experiments were used primarily to visualize and quantify treatment-dependent changes in nuclear pRPA intensity. Although they reproducibly show reduced pRPA in the PARGi-treated samples, the microscopy data were not sufficiently robust for PAR–pRPA correlation analysis, likely because of the limited dynamic range of the PAR signal in the microscopy images. Therefore, we did not include a microscopy-based correlation plot, to avoid misinterpreting these data.

- Figure 3 A is lacking cytoplasmic/nuclear controls. While the chromatin fractionation was clearly successful, it is puzzling to see more (phospho and total) RPA32 and ADP-ribosylated PARP1 in the cytoplasm than in the nuclear fraction.

To improve the assessment of the fractionation, we added tubulin as a cytoplasmic marker together with H3 as a marker for the nuclear/chromatin-containing fractions in the revised figure set (now in **Fig EV1C**, together with Ponceau S staining). A likely explanation for the relatively strong RPA32 and ADP-ribosylated PARP1 signals in the soluble fraction is that the first extraction step of the sequential fractionation protocol releases not only cytoplasmic proteins but also a substantial fraction of soluble nucleoplasmic proteins. Therefore, these signals cannot be interpreted as evidence for cytoplasmic localization of RPA32 or PARP1.

Importantly, the conclusions drawn from this experiment rely on the chromatin-bound fraction, where HU-induced RPA32 accumulation and its reduction by PARG inhibition are clearly observed. We have revised the figure presentation accordingly to include the relevant fractionation/loading controls.

- The first paragraph of the introduction contains long and complicated sentences, please rephrase for better readability.

We thank the reviewer for this helpful suggestion. The first paragraph of the Introduction has been substantially revised to improve readability and flow. Several long and complex sentences were rewritten into shorter statements, and the sequence of concepts was reorganized to provide a clearer introduction to replication stress, ATR–CHK1 signaling, and fork collapse.

Referee #2:

Mihut et al. present PARGi-dependent changes of RPA phosphorylation and its chromatin binding upon induced replication stress. Specifically, they show in P19 mouse teratocarcinoma cells that hydroxyurea-induced phosphorylation of RPA32 at T21 and S4/S8 is reduced after PARGi and propose a feedback loop between declining ATR activity after replication fork collapse, enhanced PARylation, RPA release from DNA, and a further reduction in ATR signaling. Although potentially interesting, the proposed feedback loop is not sufficiently substantiated, the relevance of the conditions in which it may occur is unclear, and the observations seem cell line-specific, putting the generalizability into question.

Main points:

1) The PARGi-induced reduction in pRPA T21 and pS4/S8 and its correlation with reduced ATR signaling (pCHK1) is only observed in a single carcinoma cell line. The relevance and generalizability of this observation thus remain unclear.

We thank the reviewer for raising this point. We would like to clarify that the original submission already included analyses in additional cell lines beyond P19, including HeLa and RPE-1, although these data may not have been sufficiently emphasized in the previous version. These cell lines required increased incubation time (>16h) and inhibition of ATR/CHK1 to detect the PARGi-induced reduction in phosphorylated RPA forms or chromatin-bound RPA.

Our results show that the PARGi-induced reduction in pRPA and associated signaling phenotype is not exclusive to P19 cells. Rather, in HeLa and RPE-1 cells the phenotype becomes evident under conditions where prolonged replication stress is combined with ATR or CHK1 inhibition, i.e. when checkpoint maintenance is compromised. Under these conditions, PARGi promotes PAR accumulation together with reduced phosphorylated RPA

and decreased chromatin-bound RPA, recapitulating the key features observed in P19 cells. These findings suggest that the phenomenon is **context-dependent rather than cell-line specific**, with checkpoint competence and fork stability being the major determinants. To make this clearer, we reorganized and expanded these data in the revised manuscript and strengthened the corresponding discussion.

Relevant data are shown in **Fig. 3B, Fig. EV3A-B, and Fig. 4B**.

2) The reduction in pRPA T21 and pS4/S8 and its correlation with reduced ATR signaling (pCHK1) is only observed upon PAR stabilization through PARGi, in presence of HU or APH. Whether physiological PARylation upon fork stalling and collapse would ever affect RPA without PARGi remains unclear. The combination of HU/APH with PARGi seems quite artificial and it is difficult to imagine conditions where cells would be faced with such a challenge.

We thank the reviewer for this important point regarding physiological relevance. We agree that acute co-treatment with HU/APH and PARGi represents an experimental perturbation designed to stabilize PAR chains and thereby reveal the consequences of defective PAR turnover under replication stress.

Our revised data indicate that the key phenotype is not restricted to pharmacological PARG inhibition**.** During prolonged HU treatment of P19 cells, PARylation became readily detectable even in the absence of PARGi, and this was accompanied by declining pRPA and pCHK1 levels over time. In contrast, when PARylation was suppressed genetically (PARP1 knockout) or pharmacologically (PARPi), prolonged HU treatment did not lead to the marked decline in pRPA observed in cells with intact PARP1 activity. These findings suggest that sustained replication stress can generate an endogenous PARylation-dependent state that influences RPA signaling, whereas PARG inhibition primarily enhances or unmasks this process by preventing PAR turnover.

These data are shown in **Fig. 2C**.

3) In some of the assays the effect seems quite moderate. For instance, the differences in pRPA T21 plus/minus PARGi in Figure 1E are small. In Figure 1G, please show the HU-only conditions for comparison. In Figure 2C, second and third lane, hardly any difference in pRPA can be seen. For the IF and FACS experiments, in which pRPA T21 and poly-ADPr were detected simultaneously, how can the authors be sure that there is no steric competition between the antibodies, which could explain reduced pRPA T21 signals in cells with high poly-ADPr signals?

We agree that in FACS analyses the change in the **percentage of pRPA-T21-positive cells** is modest. However, the principal effect is not a large loss of positive cells, but a **reduction in pRPA signal intensity within the affected population**, particularly at later time points and in cells with the highest PAR accumulation. To better illustrate this, we now present overlay histograms / intensity distributions showing the shift in pRPA-T21 signal after PARG inhibition (**Fig. 2E**), while the original dot plots were moved to **Fig. EV2C**.

As requested, the corresponding **HU-only conditions** have now been included alongside the HU+PARGi samples in the revised figure set (**now Fig. 2D**), allowing direct comparison.

Regarding the early time-point experiment (former Fig. 2C; now **Fig. EV1A-B**), we agree that the pRPA reduction is limited at 1.5 h. This experiment was intended primarily to compare the early kinase signaling responses (ATR/CHK1, ATM/CHK2, DNA-PK) rather than to demonstrate the maximal PARGi effect on pRPA, which becomes more evident after longer treatment.

Finaly, to address the possibility of steric competition between the anti-pRPA and anti-poly(ADP-ribose) antibodies in IF/FACS experiments, we performed single-staining controls

(**Rebuttal Figs. R2 and R3**). These confirmed that detection of each signal is retained independently and that the reduced pRPA signal in PAR-high cells is not due to antibody interference. Importantly, the same reduction in pRPA is also detected by western blotting and supported by chromatin fractionation, providing independent validation of the phenomenon.

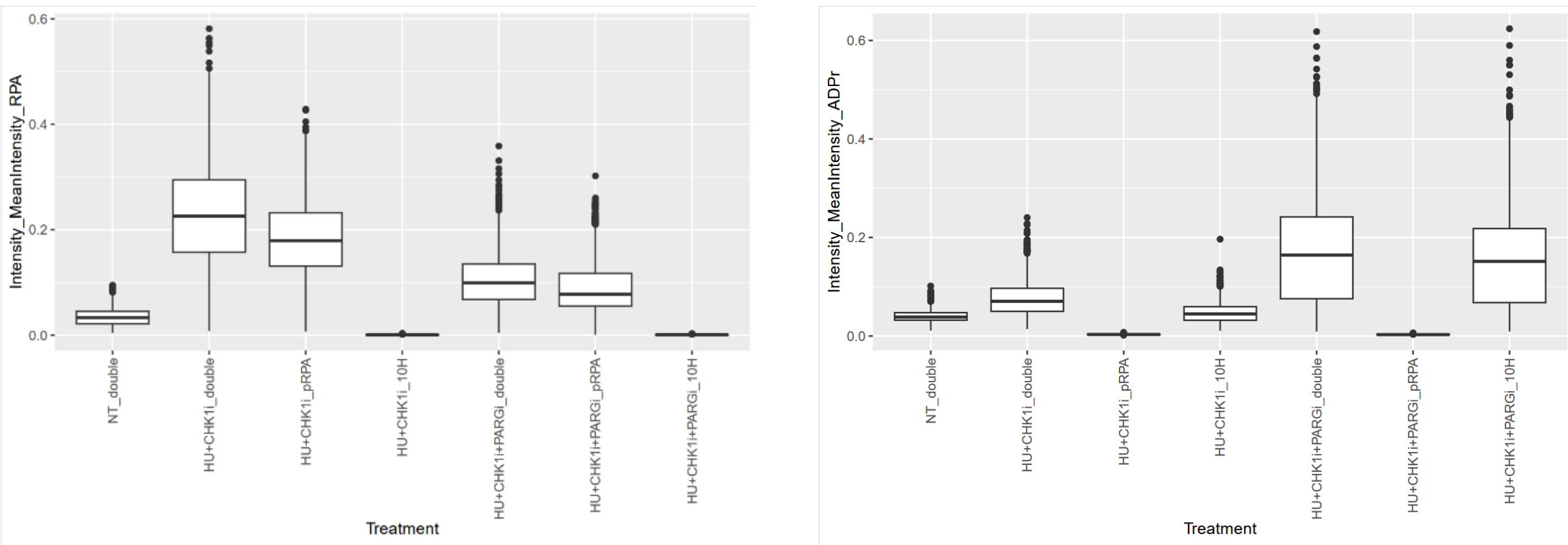

Fig. R2: Quantification of pRPA-T21 and ADPr mean fluorescence intensities. P19 cells were treated with HU (0.5 mM) and CHK1i (5 μM) with or without PARGi (5 μM) for 4 hours assessed by immunofluorescence. Micrographs of single antibody-stained samples (stained with either pRPA-T21 or anti-PAR 10H antibodies) and doubly stained samples (double) were analyzed using CellProfiler, where the DAPI-stained DNA was used to segment the nuclei and measure the mean intensities of pRPA-T21 or poly-ADPr therein. The NT sample was left untreated and was doubly stained. The quantification of randomly selected 2000 nuclei per condition were plotted using R in RStudio.

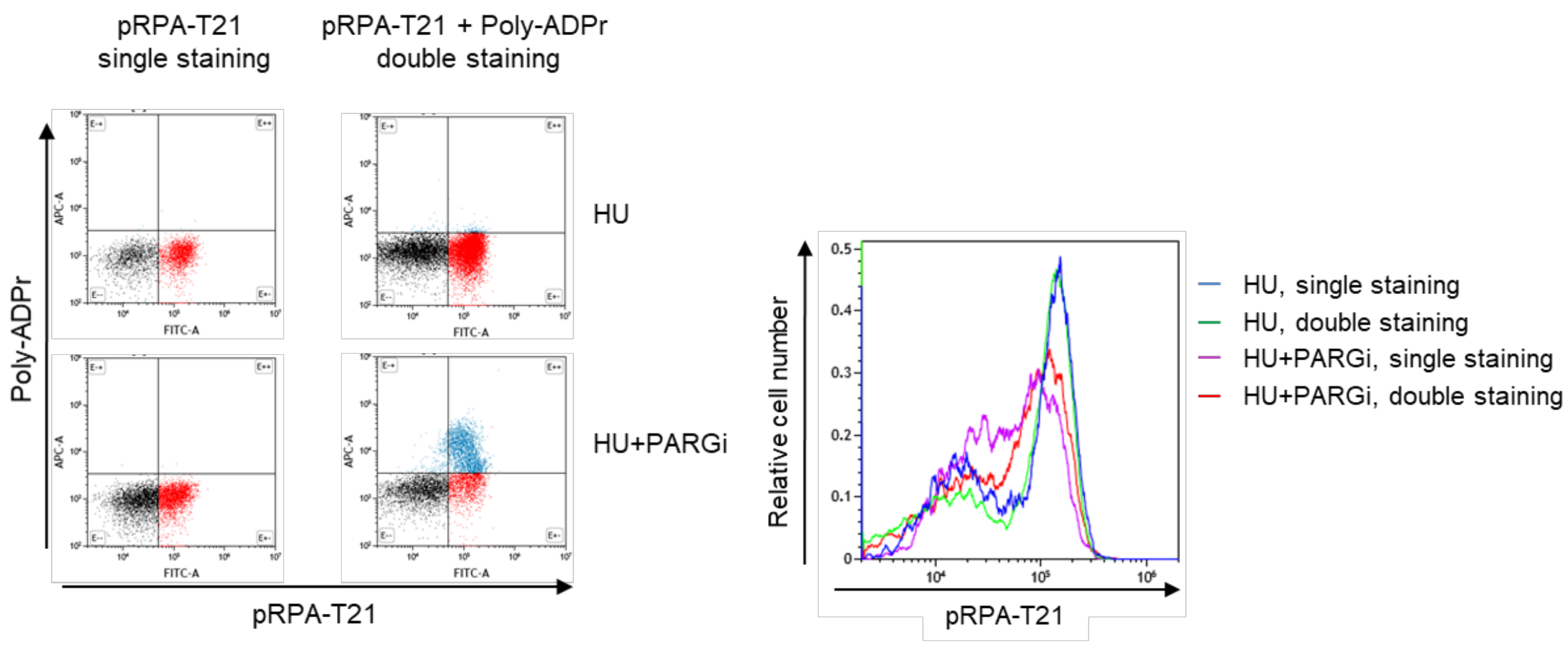

Fig. R3: Comparison of pRPA-T21 fluorescence intensities in single vs. double antibody-stained samples. P19 cells were treated with HU (0.5 mM) in the presence or absence of PARGi (5 μM) for 4 hours, then fixed, stained with either pRPA-T21 antibody alone or in combination with Poly-ADPr antibody, and then analyzed by flow-cytometry. Left: Dot plots of pRPA-T21 and poly-ADPr fluorescence intensity show the inverse correlation of the signals. Right: Overlay histograms of the pRPA-T21 signal intensities show that poly-ADPr antibody does not interfere with the pRPA-T21 signal.

4) Many of the results are not particularly novel or unexpected, e.g. that HU (fork stalling and uncoupling) and CPT at the dose used (mainly DSBs) reflect different outcomes and induce different signaling (ATR-CHK1 at stalled forks, ATM-CHK2 at DSBs), and that replication stress combined with checkpoint inhibition promotes fork collapse and PARP1 activation.

We agree that several individual signaling relationships cited in the comment are well established, including the differential responses typically induced by HU versus CPT and the role of checkpoint inhibition in promoting fork collapse (we included the references: Zhao & Piwnica-Worms, 2001; Pommier, 2006). Our intention in these experiments was not to claim

these baseline responses as novel, but rather to establish distinct replication stress contexts in which the consequences of sustained PARylation could be compared in the P19 cell line. The key findings of our study emerge from this comparative framework.

Specifically, we show that:

1. **The effect of PARG inhibition is highly stress-context dependent**, being most pronounced under fork-stalling conditions (HU, APH) and less evident in topoisomerase poison-induced responses (CPT, ETO).
2. **Checkpoint competence is a major determinant of the phenotype**: when ATR–CHK1 signaling is maintained, cells are relatively resistant to the PARGi-associated loss of RPA, whereas checkpoint suppression unmasks this state in HeLa and RPE-1 cells.
3. Sustained PARylation is associated with **reduced ssDNA exposure, reduced chromatin-bound RPA, and attenuated ATR signaling**, linking PAR turnover to the regulation of damaged-fork states.
4. Prolonged HU treatment without PARGi can also generate aspects of this phenotype, indicating that the mechanism is not solely a pharmacological artifact.

5) On the other hand, the mechanism how persistent PAR chains under conditions of replication stress plus PARGi limit RPA loading on chromatin remains unclear, and indirect effects (e.g. loss of ssDNA due to massive DNA damage, NAD+/ATP depletion due to stabilized PAR chains) are possible.

We thank the reviewer for this important mechanistic point. We agree that the precise molecular pathway by which sustained PARylation reduces chromatin-bound RPA remains to be fully resolved, and we have revised the Discussion to more clearly distinguish supported conclusions from speculative models.

At the same time, we performed additional experiments to address the indirect explanations raised by the reviewer. First, to test whether metabolic NAD^+ depletion could account for the phenotype, we depleted cellular NAD^+ using the NAMPT inhibitor FK866 prior to HU ± PARGi treatment. Under these conditions, PAR formation was strongly suppressed, yet HU-induced RPA phosphorylation was still retained (**Rebuttal Fig R4**). Moreover, PARGi no longer reduced pRPA in NAD^+-depleted cells. These results argue against simple NAD^+/ATP depletion as the cause of reduced RPA loading and instead support a requirement for stabilized PAR chains themselves. As these experiments were performed primarily as a control to address the Reviewer's specific question and do not alter the main conclusions, we report them here in the rebuttal.

Second, to examine whether altered DNA structure contributes to the phenotype, we performed native BrdU staining under checkpoint-compromised conditions in which PARGi reduces chromatin-bound RPA. In these experiments, PARGi also reduced the native BrdU signal, indicating decreased ssDNA exposure accompanying the loss of RPA. Together, these data suggest that the phenotype is more consistent with a PAR-dependent transition to a fork state with reduced ssDNA/RPA rather than a nonspecific metabolic consequence of PAR accumulation. Relevant data are included in **Fig. 4C** and **Fig. EV4A** and discussed in the revised Discussion.

Figure for referee with unpublished data and its description has been removed upon request by the authors.

6) For a more complete picture of replication dynamics in the conditions studied, DNA fiber or DNA combing assays to look at replication fork speed, fork remodeling, ssDNA gaps, and origin firing would be needed.

We thank the reviewer for this constructive suggestion. We expanded the manuscript with multiple DNA fiber assays to directly examine replication dynamics under the studied conditions.

First, we performed conventional fiber spreading assays in P19 cells during HU or CPT treatment to assess fork progression. These experiments showed that HU caused near-complete suppression of track elongation, whereas CPT induced partial slowing of fork progression. In both settings, PARG inhibition further reduced track lengths, consistent with impaired fork progression and/or recovery (**Fig. 1B**).

Second, we implemented a three-label fiber assay to distinguish stalled forks, restarted forks, and newly fired origins after HU release. These experiments revealed marked differences between cell lines. In P19 cells, only a small fraction of forks resumed replication after 4 h HU treatment, indicating extensive fork collapse and reliance on new origin firing after release. In contrast, RPE-1 cells retained a substantially greater capacity for fork restart, while PARG inhibition reduced this recovery (**Fig. 5E**). These results correlate well with the flow-cytometric analysis of BrdU incorporation (**Fig. EV1D**).

While informative, direct visualization of fork-remodeling intermediates (such as reversed forks) or ssDNA gaps would require specialized methodologies, including electron microscopy or dedicated DNA combing approaches, which we felt were beyond the scope of the present study.

7) Information on replicate experiments and reproducibility seems missing.

We thank the reviewer for highlighting this important point. To improve transparency and reproducibility, we have revised the figure legends throughout the manuscript to explicitly indicate the number of independent biological replicates, the quantification approach and the statistical analysis where applicable.

8) Overall, the manuscript is rather observational, descriptive and the text difficult to follow. Some of the sections read as long lists of observations and the coherence could be improved.

We thank the reviewer for this assessment. In response, we substantially revised the manuscript to improve clarity, coherence, and overall narrative flow.

Specifically, the Results section has been reorganized to follow a clearer mechanistic progression, moving from the stress-type dependence of the phenotype, to checkpoint-context dependence, to replication dynamics and fork recovery. We aimed to reduce redundant parts and rewrote several sections to improve clarity. We also revised the Introduction and Discussion to better frame the central question, highlight the main advances of the study, and more explicitly acknowledge mechanistic limitations where appropriate. We hope that these revisions have made the manuscript substantially easier to follow.

Minor points:

1) Introduction, line 4, is the proofreading function of the polymerase relevant for helicase-polymerase uncoupling?

We thank the reviewer for noting this imprecise wording. We did not intend to imply a role for the proofreading activity of the polymerase in helicase–polymerase uncoupling. The sentence has been revised for clarity and accuracy in the Introduction.

2) Introduction, line 13, DNA replication seems unlikely to resume after replication catastrophe, and the sentence ("In such cases, ...") is misleading.

Thank you for pointing this out. The Introduction has been revised to clarify this distinction and to avoid the misleading implication that replication resumes after replication catastrophe.

3) PARylation was previously linked to CHK1 retention at replication forks and its activation (PMID: 24356582). How do the authors reconcile this with their own data? This should be discussed.

We thank the reviewer for raising this important point. We do not view these findings as contradictory, but rather as reflecting different replication stress contexts.

The study by Min et al. (2013) showed that PAR can promote CHK1 retention and efficient checkpoint activation at newly stalled replication forks. In contrast, our study primarily examines prolonged replication stress conditions in which checkpoint maintenance declines and forks transition toward damaged or collapse-associated states. Under these later conditions, sustained PARylation is accompanied by reduced ssDNA exposure, reduced chromatin-bound RPA, and attenuated CHK1 phosphorylation. We therefore interpret PAR signaling as potentially having distinct consequences at different stages of the replication stress response: supporting early checkpoint activation at stalled forks, while being associated with impaired ATR–CHK1 signaling once stress persists and fork integrity is lost.

We have incorporated this discussion into the revised manuscript.

4) Studies from Caldecott and Hanzlikova, using PARGi to reveal PARylation in the context of replication (PMIDs: 29983321, 35332322) should also be discussed.

The cited studies have been incorporated into the revised manuscript. These seminal works used PARG inhibition to reveal endogenous PARylation during DNA replication and linked PARP activity to the processing and integrity of nascent DNA strands, particularly in the context of Okazaki-fragment maturation and post-replicative strand discontinuities. We cite and discuss these findings to better position our study, which focuses on a distinct but potentially related setting: replication stress induced by prolonged HU treatment, where sustained PARylation is associated with damaged-fork states, reduced RPA loading, and altered checkpoint signaling.

Referee #3:

This is a very interesting manuscript examining the inter-relationship between PARylation and ATR activation. In a way, both PARylation and ATR activation can be considered as alarm systems (checkpoints) for various forms of DNA damage and DNA replication stress. In this regard, cells may have feedback systems, such as when one pathway is activated, the other is suppressed. This manuscript describes such a feedback mechanism. Overall, I find that this manuscript is timely and should be considered for publication, once the following points below are addressed.

Specific Points:

1. Some statements in the Introduction seem self-conflicting. For example, the sentences: "ATR checkpoint failure during replication stress leads to fork collapse. Concomitantly, RPA exhaustion due to uncontrolled origin firing, deregulated fork remodeling, fork component disintegration, to name a few, will generate unrepaired DNA breaks at the fork and collapse it", are followed by: "In such cases, replication can resume through activation of a nearby dormant origin or via fork restart mechanisms that stabilize and reactivate the stalled fork". How did a collapsed fork (first two sentences), become a stalled fork (third sentence)? A stalled fork is usually thought to be a fork that has not yet collapsed.

We agree that the original wording did not distinguish stalled forks from irreversibly collapsed forks and therefore created ambiguity in the sequence of events. The Introduction has been revised to clarify that ATR–CHK1 signaling functions to stabilize stressed forks, whereas failure of this checkpoint promotes fork collapse and marks the transition from replication stress to DNA damage. We also removed wording that could imply direct reactivation of collapsed forks.

2. Fig. 1A shows that a PARG inhibitor (PARGi) combined with HU treatment, decreased the levels of CHK1 and RPA phosphorylation (pCHK1 ad pRPA) induced by HU alone. This effect was seen only at later time points. Fig. 1B and 1C confirm this observation and further show that, in the absence of PARP1 or when PARP1 is inhibited, the PARGi does not decrease the levels of pRPA in HU-treated cells. Hence, high levels of PARylation are important for the decrease in pRPA (and pCHK1) levels driven by PARGi in HU-treated cells.

We thank the reviewer for this accurate summary of our findings and agree with this interpretation. Our data indicate that elevated and persistent PARylation is required for the reduction in pRPA and pCHK1 observed during HU treatment in the presence of PARG inhibition. Consistent with this, the effect is lost in PARP1-deficient cells or upon PARP inhibition, indicating dependence on PAR synthesis. In addition, in the revised manuscript we show that prolonged HU treatment alone can also lead to detectable PAR accumulation accompanied by declining pRPA and pCHK1 levels, supporting the idea that this relationship is not restricted to acute pharmacological PARG inhibition (**Fig. 2C**). Together, these observations support our model that sustained PARylation contributes to attenuation of the ssDNA/RPA/ATR signaling axis during prolonged replication stress.

3. Fig. 2 extends the observations of Fig. 1. Treatment of cells with aphidicolin (Aph) and PARGi showed similar effects as treatment with HU and PARGi, indicating that the agent that induces stalling of DNA replication is not important. The easiest interpretation of these findings is that PARGi prevents processing of stalled/collapsed forks, such that there is less DNA end resection, less single-stranded DNA (ssDNA) and less activation of ATR. The results of Fig. 3

support this interpretation, as chromatin bound RPA levels decreased by PARGi in cells treated with HU.

We thank the reviewer for this thoughtful interpretation. We hypothesize that the signal of chromatin bound RPA can originate from various sources: the uncoupling of DNA polymerases and helicases upon HU or APH treatment, fork reversal or the DNA end resection upon dsDNA breaks.

We could not detect the increase of Rad51 along with the reduction of pRPA during HU+PARGi+CHKi treatment nor the requirement of BRCA1 in this process (**Fig. 4A, B**), hence ruling out replacement of pRPA by Rad51.

We agree that reduced generation ssDNA provides a plausible explanation for the decreased RPA loading and attenuated ATR signaling observed under PARGi-sensitive conditions. To directly address this possibility, we performed native BrdU staining in conditions where PARGi reduces chromatin-bound RPA. These experiments showed that PARGi was accompanied by a reduction in the native BrdU signal, consistent with decreased ssDNA exposure (**Fig. 4C**).

At present, our data do not allow us to distinguish whether this reflects reduced DNA end resection, altered fork uncoupling, enhanced fork remodeling, or another damaged-fork transition. We have therefore revised the Discussion to more explicitly discuss reduced ssDNA availability as a possible contributor to the loss of RPA and attenuated ATR signaling under PARGi-sensitive conditions.

4. Fig. 4 provides data indicating that inhibiting ATR in HU-treated cells leads to firing of new origins, which is consistent with previous results.

We agree that loss of checkpoint restraint is expected to favor compensatory origin firing during replication stress, consistent with prior studies. In the revised manuscript, we further strengthened this section with three-label DNA fiber assays to distinguish ongoing forks, restarted forks, and newly fired origins after HU treatment and release (**Fig. 5E**). These analyses show that, particularly in P19 cells, recovery after HU stress relies strongly on new origin firing because relatively few stalled forks resume replication, whereas RPE-1 cells retain substantially greater fork restart capacity.

5. In the Discussion, the authors provide various models why PARGi reduce the levels of chromatin-bound RPA in HU-treated cells, but do not consider the model described in point 3 above. There are methods to monitor the presence of ssDNA, for example treating the cells with BrdU and then monitoring the presence of ssDNA by immunofluorescence under non-denaturing conditions. Perhaps, the authors could try such an experiment, as this could help distinguish between the model in point 3 above and the models cited by the authors in the Discussion.

We thank the reviewer for this suggestion. We performed native BrdU staining under non-denaturing conditions to directly monitor ssDNA in the settings where PARGi reduces chromatin-bound RPA. These experiments showed that PARGi was accompanied by a reduction in the native BrdU signal under conditions where pRPA and chromatin-bound RPA were also decreased, indicating reduced ssDNA exposure (**Fig. 4C and Fig. EV4A**). While this assay does not by itself distinguish whether the reduced ssDNA reflects decreased DNA end resection, altered fork uncoupling, enhanced fork remodeling, or other damaged-fork transitions, it strongly supports the reviewer’s central suggestion that reduced ssDNA availability contributes to the loss of RPA and attenuated ATR signaling. We have revised the Discussion accordingly to more explicitly consider this model.

Dear Dr. Timinszky,

Thank you for the submission of your revised manuscript to our editorial offices. I have now received the reports from the three referees that were asked to re-evaluate the study, you will find below. As you will see, the referees now support the publication of the study in EMBO reports. Referee #2 has remaining minor concerns and suggestions to improve the manuscript I ask you to address in a final revised version. Please also provide a final p-b-p-response to these points and the editorial requests below.

Editorial requests:

- Please provide a final title with not more than 100 characters (including spaces).

- There is a name discrepancy. It is Montero-Hernández on the manuscript title page and Montero-Hermández in the submission system. Please check and make sure that in both places the same name is used.

- Please provide the abstract written in present tense throughout.

- Please order the manuscript sections like this, using only these names:
Title page - Abstract - Keywords - Introduction - Results - Discussion - Methods - Data availability section - Acknowledgements (please include here all the funding information) - Disclosure and Competing Interests Statement - References - Figure legends - Expanded View Figure legends

- We now use CRediT to specify the contributions of each author in the journal submission system. CRediT replaces the author contribution section. Please use the free text box to provide more detailed descriptions and do NOT provide your final manuscript text file with an author contributions section. See also our guide to authors (section 'Author contributions'):
https://link.springer.com/journal/44319/submission-guidelines#cms-Revised-submissions

- Please check again that the number "n" for how many independent experiments were performed, their nature (biological versus technical replicates), the bars and error bars (e.g. SEM, SD) and the test used to calculate p-values is indicated in the respective figure legends (main and EV figures). Please also check that all the p-values are explained in the legend, and that these fit to those shown in the figure. Please provide statistical testing where applicable. Please avoid the phrase 'independent experiment' but clearly state if these were biological or technical replicates. Please also indicate (e.g. with n.s.) if testing was performed, but the differences are not significant. In case n=2, please show the data as separate datapoints without error bars and statistics. See also:

https://link.springer.com/journal/44319/submission-guidelines#cms-Figure-and-data-presentation

If n<5, please show single datapoints for diagrams. Moreover:
- Please note that the exact p values are not provided in the legend of figure 1B
- Please show the 2 datasets (n=2) in figure 5E separately and remove the error bars.

- Please confirm that for all Western blot panels shown the loading control was run on the same gel as the other proteins detected. Please note that we discourage comparisons between samples on different gels/blots, even if the samples derive from one experiment, as confounding factors reduce comparability. If unavoidable, the figure legend must state that the samples derive from the same experiment and that gels/blots were processed in parallel. If a 'representative' loading control is shown for multiple gels/blots, the intra-gel controls should be shown in the source data files, and the figure legends should describe the data displayed accurately. See our author guidelines:

https://link.springer.com/journal/44319/submission-guidelines#cms-Figure-and-data-presentation (section 'Electrophoretic gels and blots').

In addition, I would need from you uploaded separately:
- a short, two-sentence summary of the manuscript (not more than 35 words).
- two to four short (!) bullet points highlighting the key findings of your study (two lines each).
- a schematic summary figure as separate file that provides a sketch of the major findings (not a data image) in jpeg or tiff format (with the exact width of 550 pixels and the exact height of 300 pixels) that can be used as a visual synopsis on our website.

I look forward to seeing the further revised version of your manuscript when it is ready. Please let me know if you have questions regarding the revision.

Best,

Achim Breiling
Senior Editor
EMBO Reports

Referee #1:

The authors have carefully addressed the concerns raised in the initial review. In particular, the revised manuscript now more clearly positions the work relative to Illuzzi et al. and better highlights the conceptual advance regarding the relationship between sustained PARylation, reduced RPA loading, and attenuation of ATR-CHK1 signaling.

The additional analyses, revised quantifications, expanded discussion, and new experiments substantially strengthen the manuscript and improve clarity throughout. I also appreciate the authors' efforts to better define the context dependence of the phenotype and to address possible alternative explanations.
Overall, I find that my major concerns have been satisfactorily addressed and that the manuscript is now suitable for publication in EMBO Reports.

Referee #2:

The substantially revised manuscript is significantly improved. Several of the original interpretations are now better substantiated and the readability (e.g. of the introduction) has improved. Pending relatively minor corrections and clarifications, this reviewer is supportive of publication.

1) The main claim that "sustained PARylation ... attenuates ATR-CHK1 signaling", as reflected in the title, was primarily demonstrated in P19 cells. To reveal the role of PARylation in HeLa and RPE-1 cells, inhibition of ATR or CHK1 was required, which, in this reviewer's opinion, precludes any conclusion about the effect of PARylation on ATR-CHK1 signaling. Unless attenuated ATR-CHK1 signaling can be demonstrated in ATR-CHK1 signaling-proficient HeLa and RPE-1 cells, the generalizability of this claim is not sufficiently supported, and title, abstract (last sentence), and parts of the discussion should be revised accordingly.
2) On several occasions, the manuscript talks about loss of RPA or decline in RPA or reduction in RPA without specifying whether this relates to the total pool of RPA or to chromatin-/ssDNA-bound RPA. Please clarify.
3) For the discussion on page 14, third paragraph, could not both models in principle be true - reduced ssDNA and reduced RPA retention? The second half of this paragraph could be phrased in a less dichotomic and more reconciliatory manner.
4) In the Western blot shown in Figure EV3A, when looking at the pRPA-S33 band pattern, it seems as if RPA is hyper-phosphorylated/modified upon sustained PARylation. If true, an alternative interpretation of reduced pRPA signals could be that phospho-epitope-specific antibodies (e.g. the one used against pRPA-T21 throughout the manuscript) may not detect the hyper-modified form. Can the authors please comment and clarify? Unless this possibility can be ruled out, it should be mentioned in the manuscript as a potential limitation.
5) If space permits, Rebuttal Fig. R3 could be included in the manuscript as EV or Supplementary figure.
6) In the discussion, where the authors discuss that "sustained PARylation promotes fork reversal with increased strand reannealing, reduced parental ssDNA exposure, and consequently diminished RPA loading", it would make sense to also discuss the possibility that sustained PARylation limits access of nucleases to act on stalled/reversed forks. Several nucleases have been described to process replication forks and thus affect ssDNA and RPA binding.

Referee #3:

The authors have addressed my comments.

I consider the manuscript suitable for publication.

I only wonder, if the authors can draw a cartoon to indicate what they think happens at the replication fork.

Point by point response (Nr 2) to the editorial and reviewer requests for the manuscript "*Sustained poly(ADP-ribosyl)ation limits RPA loading and attenuates ATR–CHK1 signaling upon replication fork collapse*" by Mihuț et al.

Below is our point-by-point response (in blue) to the reviewers' comments (shown in black).

Point-by-point response

Editorial requests:

- Please provide a final title with not more than 100 characters (including spaces).

PARG inhibition reduces ssDNA levels and limits RPA loading upon replication fork collapse

- There is a name discrepancy. It is Montero-Hernández on the manuscript title page and Montero-Hermández in the submission system. Please check and make sure that in both places the same name is used.

We are sorry but we cannot identify the indicated name discrepancy. We only found one difference: **Dávid** Szüts was **David** Szüts in the submission system. It should be **Dávid**, but I cannot change it in the submission system.

- Please provide the abstract written in present tense throughout.

The abstract has been rewritten in present tense.

- Please order the manuscript sections like this, using only these names:
Title page - Abstract - Keywords - Introduction - Results - Discussion - Methods - Data availability section - Acknowledgements (please include here all the funding information) - Disclosure and Competing Interests Statement - References - Figure legends - Expanded View Figure legends

Done.

- We now use CRediT to specify the contributions of each author in the journal submission system. CRediT replaces the author contribution section. Please use the free text box to provide more detailed descriptions and do NOT provide your final manuscript text file with an author contributions section. See also our guide to authors (section 'Author contributions'): https://link.springer.com/journal/44319/submission-guidelines#cms-Revised-submissions

Done.

- Please check again that the number "n" for how many independent experiments were

performed, their nature (biological versus technical replicates), the bars and error bars (e.g. SEM, SD) and the test used to calculate p-values is indicated in the respective figure legends (main and EV figures). Please also check that all the p-values are explained in the legend, and that these fit to those shown in the figure. Please provide statistical testing where applicable. Please avoid the phrase 'independent experiment' but clearly state if these were biological or technical replicates. Please also indicate (e.g. with n.s.) if testing was performed, but the differences are not significant. In case n=2, please show the data as separate datapoints without error bars and statistics. See also:

https://link.springer.com/journal/44319/submission-guidelines#cms-Figure-and-data-presentation

If n<5, please show single datapoints for diagrams. Moreover:
- Please note that the exact p values are not provided in the legend of figure 1B
- Please show the 2 datasets (n=2) in figure 5E separately and remove the error bars.

We modified the figure legends to clearly state which types of replicates were performed. P values in Figure 1B are shown in the figure above the comparison. All P values were calculated by GraphPad Prism, which does not show an exact number, when the P value is too low, e.g., p<0.0001, which corresponds to **** significance. Star designation was added to the figure legend. In Figure 5E, we removed error bars and changed the shapes of symbols to clearly distinguish individual biological replicates.

- Please confirm that for all Western blot panels shown the loading control was run on the same gel as the other proteins detected. Please note that we discourage comparisons between samples on different gels/blots, even if the samples derive from one experiment, as confounding factors reduce comparability. If unavoidable, the figure legend must state that the samples derive from the same experiment and that gels/blots were processed in parallel. If a 'representative' loading control is shown for multiple gels/blots, the intra-gel controls should be shown in the source data files, and the figure legends should describe the data displayed accurately. See our author guidelines:

https://link.springer.com/journal/44319/submission-guidelines#cms-Figure-and-data-presentation (section 'Electrophoretic gels and blots').

We could not run all WB panels on the same gel. This is now clearly stated in the figure legends and all original data including in-gel controls are shown in the source data.

In addition, I would need from you uploaded separately:
- a short, two-sentence summary of the manuscript (not more than 35 words).

Done.
- two to four short (!) bullet points highlighting the key findings of your study (two lines each).

Done.
- a schematic summary figure as separate file that provides a sketch of the major findings (not a data image) in jpeg or tiff format (with the exact width of 550 pixels and the exact height of 300 pixels) that can be used as a visual synopsis on our website.

Done.

Referee #1:

The authors have carefully addressed the concerns raised in the initial review. In particular, the revised manuscript now more clearly positions the work relative to Illuzzi et al. and better highlights the conceptual advance regarding the relationship between sustained PARylation, reduced RPA loading, and attenuation of ATR-CHK1 signaling.

The additional analyses, revised quantifications, expanded discussion, and new experiments substantially strengthen the manuscript and improve clarity throughout. I also appreciate the authors' efforts to better define the context dependence of the phenotype and to address possible alternative explanations.
Overall, I find that my major concerns have been satisfactorily addressed and that the manuscript is now suitable for publication in EMBO Reports.

We thank the reviewer for their support.

Referee #2:

The substantially revised manuscript is significantly improved. Several of the original interpretations are now better substantiated and the readability (e.g. of the introduction) has improved. Pending relatively minor corrections and clarifications, this reviewer is supportive of publication.

We would like to thank the reviewer for their careful reading of our manuscript and their most constructive suggestions to further improve it.

1) The main claim that "sustained PARylation ... attenuates ATR-CHK1 signaling", as reflected in the title, was primarily demonstrated in P19 cells. To reveal the role of PARylation in HeLa and RPE-1 cells, inhibition of ATR or CHK1 was required, which, in this reviewer's opinion, precludes any conclusion about the effect of PARylation on ATR-CHK1 signaling. Unless attenuated ATR-CHK1 signaling can be demonstrated in ATR-CHK1 signaling-proficient HeLa and RPE-1 cells, the generalizability of this claim is not sufficiently

supported, and title, abstract (last sentence), and parts of the discussion should be revised accordingly.

This is a point our team discussed and debated many times. We agree that the attenuation of ATR–CHK1 signaling by sustained PARylation was demonstrated most directly in P19 cells. It should be noted that in the P19 cell line ATR–CHK1 signaling gets attenuated over time, which might be a prerequisite for PARGi to have the observed effect.

We also agree with the reviewer that the HeLa and RPE-1 experiments performed in the presence of ATR or CHK1 inhibitors do not establish that sustained PARylation attenuates the intact ATR–CHK1 checkpoint in signaling-proficient cells. However, we would like to point out that in RPE-1 cells PARG and CHK1 co-inhibition reduced CHK1 phosphorylation (that was abolished by ATR inhibition, indicating that this pCHK1 signal reflects ATR-dependent phosphorylation) in Figure 3B. The reduction of this ATR-dependent pCHK1 signal by PARG inhibition under a CHK1 signaling compromised condition supports the conclusion that sustained PARylation can attenuate ATR activity (most likely though reducing ssDNA levels leading to reduced ssDNA-bound RPA, which would lead to less ATR activation). Thus, PARGi might lead to a vicious circle in cells with compromised checkpoint signaling speeding up the loss of ATR signaling and pushing cells towards replication collapse.

We agree that this claim cannot be generalized and have therefore revised the indicated sections accordingly.

2) On several occasions, the manuscript talks about loss of RPA or decline in RPA or reduction in RPA without specifying whether this relates to the total pool of RPA or to chromatin-/ssDNA-bound RPA. Please clarify.

We have revised the text as requested and specified that whether we refer to total or chromatin-bound RPA.

3) For the discussion on page 14, third paragraph, could not both models in principle be true - reduced ssDNA and reduced RPA retention? The second half of this paragraph could be phrased in a less dichotomic and more reconciliatory manner.

Thank you for pointing this out. Absolutely, both models could be true. We rewrote the text such that it upholds the validity of both models. We hope that this opinion is now better reflected in the discussion.

4) In the Western blot shown in Figure EV3A, when looking at the pRPA-S33 band pattern, it seems as if RPA is hyper-phosphorylated/modified upon sustained PARylation. If true, an alternative interpretation of reduced pRPA signals could be that phospho-epitope-specific antibodies (e.g. the one used against pRPA-T21 throughout the manuscript) may not detect the hyper-modified form. Can the authors please comment and clarify? Unless this possibility can be ruled out, it should be mentioned in the manuscript as a potential limitation.

Phospho-specific antibodies recognize short phospho-epitopes, and we cannot exclude the possibility that additional modifications near the phospho-epitope alter recognition by phospho-specific antibodies. Therefore, we mention this potential limitation in the Methods section describing the western blots. Nevertheless, we think this is not the case.

We conducted the study using more than one phospho-RPA antibody and total RPA32, which also detects the phosphorylated form as a slower migrating band in whole-cell lysates or chromatin fractions, and all show a reduction upon PARGi, in addition to IF and FACS (pRPA for P19 and RPA32 for HeLa). It is not very likely that all used antibodies would suffer from the same issue: not being able to detect hyper-modified forms of RPA. Moreover, we also observe the reduction of ssDNA using native BrdU staining upon PARGi. Therefore, we consider that the reduction of RPA phosphorylation in the presence of PARGi in cells with replication fork collapse is genuine and is the consequence of the reduced amounts of ssDNA that leads to lower levels of RPA on chromatin, which is the substrate for phosphorylation.

The reviewer likely refers to lanes 2 and 3 in EV 3A, the conditions in which CHK1 inhibition was not used, in a cell line in which we only observe pRPA reduction upon combined treatment with PARGi and CHK1i. We also noted this increase in pRPA upon PARGi alone in the manuscript, which then decreases when cells are co-treated with CHK1i (also Fig. 3B upper panel). We write: "In HU-treated RPE-1 cells, PARGi alone did not reduce the phosphorylated forms of RPA32 or CHK1; these signals were maintained or slightly increased (Fig. 3B and Fig. EV3A)." We think this to be a condition similar to that published by Min et al. in 2013 (thank you again for pointing this out to us in the previous review), where PARylation rather facilitates CHK1 activation than opposes it. Unfortunately, we have no insight at the molecular level, but it is very interesting that PARylation appears to have opposing effects depending on the state of replication forks – while forks are protected PARylation facilitates ATR-CHK1 signaling, and when replication forks collapse PARylation reduces it.

5) If space permits, Rebuttal Fig. R3 could be included in the manuscript as EV or Supplementary figure.

We included the Rebuttal Fig. R3 in the manuscript. It is now shown as EV3D.

6) In the discussion, where the authors discuss that "sustained PARylation promotes fork reversal with increased strand reannealing, reduced parental ssDNA exposure, and consequently diminished RPA loading", it would make sense to also discuss the possibility that sustained PARylation limits access of nucleases to act on stalled/reversed forks. Several nucleases have been described to process replication forks and thus affect ssDNA and RPA binding.

We thank the reviewer for this suggestion. Indeed, this could also be a possible mechanism. We added this possibility to the discussion: "Sustained PARylation may also reduce RPA loading by limiting nuclease-dependent processing of stalled or

reversed forks. Several nucleases, including CtIP–MRE11, EXO1 and DNA2, have been implicated in the processing of replication-stress-induced fork intermediates, and such processing can generate ssDNA substrates for RPA binding. Thus, the reduced chromatin-bound RPA observed after PARG inhibition may arise from at least two complementary mechanisms: increased strand reannealing during fork reversal and reduced nuclease-mediated ssDNA generation at stalled or reversed forks."

Referee #3:

The authors have addressed my comments.

I consider the manuscript suitable for publication.

I only wonder, if the authors can draw a cartoon to indicate what they think happens at the replication fork.

We thank the reviewer for the positive evaluation of our work.

We agree that a schematic could help with conceptual clarity. However, given the current level of mechanistic insight in our study, we are concerned that a replication fork drawing might unintentionally suggest structural or molecular intermediates that we have not directly demonstrated. For this reason, we feel it would be safer not to include a fork schematic in the revised manuscript to avoid potential over-interpretation.

2nd Revision - Editorial Decision 12th Jun 2026

Dr. Gyula Timinszky
HUN-REN Biological Research Centre
Institute of Genetics
Hungary

Dear Dr. Timinszky,

Thank you for the submission of your final revised manuscript to our editorial offices. I now looked through this and your final p-b-p-response and consider the remaining concerns of referee #2 and the editorial requests as adequately addressed.

I am thus very pleased to accept your manuscript for publication in the next available issue of EMBO reports. Thank you for your contribution to our journal.

Your manuscript will be processed for publication by EMBO Press. It will be copy edited and you will receive page proofs prior to publication. Please note that you will be contacted by Springer Nature Author Services to complete licensing and payment information.

You may qualify for financial assistance for your publication charges - either via a Springer Nature fully open access agreement or an EMBO initiative. Check your eligibility: https://link.springer.com/journal/44319/how-to-publish-with-us

Should you be planning a Press Release on your article, please notify the production team via this form in order to coordinate publication and release dates: https://forms.cloud.microsoft/e/eH4A8JVP6g

If you have any questions, please do not hesitate to contact the Editorial Office. Thank you for your contribution to EMBO Reports.

Yours sincerely,

Achim Breiling
Senior Editor
EMBO Reports

>>> Please note that it is EMBO Reports policy for the transcript of the editorial process (containing referee reports and your response letter) to be published as an online supplement to each paper. If you do NOT want this, you will need to inform the Editorial Office via email immediately. More information is available here: https://link.springer.com/partners/embo-press/editorial-policies#Peer%20review

EMBO Press Author Checklist

Corresponding Author Name: Gyula Timinszky, Roberta Fajka-Boja	
Journal Submitted to: EMBO Reports	
Manuscript Number: EMBOR-2025-63018V1	

USEFUL LINKS FOR COMPLETING THIS FORM
The EMBO Journal - Author Guidelines
EMBO Reports - Author Guidelines
Molecular Systems Biology - Author Guidelines
EMBO Molecular Medicine - Author Guidelines

Reporting Checklist for Life Science Articles (updated January
This checklist is adapted from Materials Design Analysis Reporting (MDAR) Checklist for Authors. MDAR establishes a minimum set of requirements in transparent reporting in the life sciences (see Statement of Task: 10.31222/osf.io/9sm4x). Please follow the journal's guidelines in preparing your
Please note that a copy of this checklist will be published alongside your article.

Abridged guidelines for figures
1. Data
The data shown in figures should satisfy the following conditions:
- the data were obtained and processed according to the field's best practice and are presented to reflect the results of the experiments in an accurate and unbiased manner.
- ideally, figure panels should include only measurements that are directly comparable to each other and obtained with the same assay.
- plots include clearly labeled error bars for independent experiments and sample sizes. Unless justified, error bars should not be shown for technical
- if n<5, the individual data points from each experiment should be plotted. Any statistical test employed should be justified.
- Source Data should be included to report the data underlying figures according to the guidelines set out in the authorship guidelines on Data

2. Captions
Each figure caption should contain the following information, for each panel where they are relevant:
- a specification of the experimental system investigated (eg cell line, species name).
- the assay(s) and method(s) used to carry out the reported observations and measurements.
- an explicit mention of the biological and chemical entity(ies) that are being measured.
- an explicit mention of the biological and chemical entity(ies) that are altered/varied/perturbed in a controlled manner.
- the exact sample size (n) for each experimental group/condition, given as a number, not a range;
- a description of the sample collection allowing the reader to understand whether the samples represent technical or biological replicates (including how many animals, litters, cultures, etc.).
- a statement of how many times the experiment shown was independently replicated in the laboratory.
- definitions of statistical methods and measures:
  - common tests, such as t-test (please specify whether paired vs. unpaired), simple χ2 tests, Wilcoxon and Mann-Whitney tests, can be unambiguously identified by name only, but more complex techniques should be described in the methods section;
  - are tests one-sided or two-sided?
  - are there adjustments for multiple comparisons?
  - exact statistical test results, e.g., P values = x but not P values < x;
  - definition of 'center values' as median or average;
  - definition of error bars as s.d. or s.e.m.

Please complete ALL of the questions below.
Select "Not Applicable" only when the requested information is not relevant for your study.

Materials

Newly Created Materials	Information included in the manuscript?	In which section is the information available? (Reagents and Tools Table, Materials and Methods, Figures, Data Availability Section)
New materials and reagents need to be available; do any restrictions apply?	Yes	Materials and Methods: CRISPR-Cas9 nickase-mediated knockout generation

Antibodies	Information included in the manuscript?	In which section is the information available? (Reagents and Tools Table, Materials and Methods, Figures, Data Availability Section)
For **antibodies** provide the following information:<br>- Commercial antibodies: RRID (if possible) or supplier name, catalogue number and or/clone number<br>- Non-commercial: RRID or citation	Yes	Materials and Methods: Reagents and Tools Table

DNA and RNA sequences	Information included in the manuscript?	In which section is the information available? (Reagents and Tools Table, Materials and Methods, Figures, Data Availability Section)
**Short novel DNA or RNA including primers, probes**: provide the sequences.	Yes	Materials and Methods: Reagents and Tools Table

Cell materials	Information included in the manuscript?	In which section is the information available? (Reagents and Tools Table, Materials and Methods, Figures, Data Availability Section)
**Cell lines:** Provide species information, strain. Provide accession number in repository **OR** supplier name, catalog number, clone number, and/**OR** RRID.	Yes	Materials and Methods: Cell lines and culture conditions
**Primary cultures:** Provide species, strain, sex of origin, genetic modification status.	Not Applicable	
Report if the cell lines were recently **authenticated** (e.g., by STR profiling) and tested for mycoplasma contamination.	Yes	Materials and Methods: Cell lines and culture conditions

Experimental animals	Information included in the manuscript?	In which section is the information available? (Reagents and Tools Table, Materials and Methods, Figures, Data Availability Section)
**Laboratory animals or Model organisms:** Provide species, strain, sex, age, genetic modification status. Provide accession number in repository **OR** supplier name, catalog number, clone number, **OR** RRID.	Not Applicable	
**Animal observed in or captured from the field:** Provide species, sex, and age where possible.	Not Applicable	
Please detail **housing and husbandry conditions**.	Not Applicable	

Plants and microbes	Information included in the manuscript?	In which section is the information available? (Reagents and Tools Table, Materials and Methods, Figures, Data Availability Section)
**Plants:** provide species and strain, ecotype and cultivar where relevant, unique accession number if available, and source (including location for collected wild specimens).	Not Applicable	
**Microbes:** provide species and strain, unique accession number if available, and source.	Not Applicable	

Human research participants	Information included in the manuscript?	In which section is the information available? (Reagents and Tools Table, Materials and Methods, Figures, Data Availability Section)
If collected and within the bounds of privacy constraints report on age, sex and gender or ethnicity for all study participants.	Not Applicable	

Core facilities	Information included in the manuscript?	In which section is the information available? (Reagents and Tools Table, Materials and Methods, Figures, Data Availability Section)
If your work benefited from core facilities, was their service mentioned in the acknowledgments section?	Yes	Acknowledgements

Design

Study protocol	Information included in the manuscript?	In which section is the information available? (Reagents and Tools Table, Materials and Methods, Figures, Data Availability Section)
If study protocol has been **pre-registered, provide DOI in the manuscript**. For clinical trials, provide the trial registration number **OR** cite DOI.	Not Applicable	
Report the **clinical trial registration number** (at ClinicalTrials.gov or equivalent), where applicable.	Not Applicable	

Laboratory protocol	Information included in the manuscript?	In which section is the information available? (Reagents and Tools Table, Materials and Methods, Figures, Data Availability Section)
Provide DOI OR other citation details if **external detailed step-by-step protocols** are available.	Not Applicable	

Experimental study design and statistics	Information included in the manuscript?	In which section is the information available? (Reagents and Tools Table, Materials and Methods, Figures, Data Availability Section)
Include a statement about **sample size** estimate even if no statistical methods were used.	Yes	Materials and Methods, Figure Legends
Were any steps taken to minimize the effects of subjective bias when allocating animals/samples to treatment (e.g. **randomization procedure**)? If yes, have they been described?	Not Applicable	
Include a statement about **blinding** even if no blinding was done.	Not Applicable	
Describe **inclusion/exclusion criteria** if samples or animals were excluded from the analysis. Were the criteria pre-established?<br><br>If sample or data points were omitted from analysis, report if this was due to attrition or intentional exclusion and provide justification.	Not Applicable	
For every figure, are **statistical tests** justified as appropriate? Do the data meet the assumptions of the tests (e.g., normal distribution)? Describe any methods used to assess it. Is there an estimate of variation within each group of data? Is the variance similar between the groups that are being statistically compared?	Yes	Materials and Methods

Sample definition and in-laboratory replication	Information included in the manuscript?	In which section is the information available? (Reagents and Tools Table, Materials and Methods, Figures, Data Availability Section)
In the figure legends: state number of times the experiment was **replicated** in laboratory.	Yes	Figure Legends
In the figure legends: define whether data describe **technical or biological replicates**.	Yes	Figure Legends

Ethics

Ethics	Information included in the manuscript?	In which section is the information available? (Reagents and Tools Table, Materials and Methods, Figures, Data Availability Section)
Studies involving **human participants**: State details of **authority granting ethics approval** (IRB or equivalent committee(s), provide reference number for approval.	Not Applicable	
Studies involving **human participants**: Include a statement confirming that **informed consent** was obtained from all subjects and that the experiments conformed to the principles set out in the WMA Declaration of Helsinki and the Department of Health and Human Services Belmont Report.	Not Applicable	
Studies involving **human participants:** For publication of **patient photos**, include a statement confirming that consent to publish was obtained.	Not Applicable	
Studies involving experimental **animals**: State details of **authority granting ethics approval** (IRB or equivalent committee(s), provide reference number for approval. Include a statement of compliance with ethical regulations.	Not Applicable	
Studies involving **specimen and field samples:** State if relevant **permits** obtained, provide details of authority approving study; if none were required, explain why.	Not Applicable	

Dual Use Research of Concern (DURC)	Information included in the manuscript?	In which section is the information available? (Reagents and Tools Table, Materials and Methods, Figures, Data Availability Section)
Could your study fall under dual use research restrictions? Please check biosecurity documents and list of **select agents and toxins** (CDC): https://www.selectagents.gov/sat/list.htm	Not Applicable	
If you used a select agent, is the security level of the lab appropriate and reported in the manuscript?	Not Applicable	
If a study is subject to dual use research of concern regulations, is the name of the **authority granting approval and reference number** for the regulatory approval provided in the manuscript?	Not Applicable	

Reporting

The MDAR framework recommends adoption of discipline-specific guidelines, established and endorsed through community initiatives. Journals have their own policy about requiring specific guidelines and recommendations to complement MDAR.

Adherence to community standards	Information included in the manuscript?	In which section is the information available? (Reagents and Tools Table, Materials and Methods, Figures, Data Availability Section)
State if relevant guidelines or checklists (e.g., **ICMJE, MIBBI, ARRIVE, PRISMA**) have been followed or provided.	Not Applicable	
For **tumor marker prognostic studies,** we recommend that you follow the **REMARK** reporting guidelines (see link list at top right). See author guidelines, under 'Reporting Guidelines'. Please confirm you have followed these guidelines.	Not Applicable	
For **phase II and III randomized controlled trials**, please refer to the **CONSORT** flow diagram (see link list at top right) and submit the CONSORT checklist (see link list at top right) with your submission. See author guidelines, under 'Reporting Guidelines'. Please confirm you have submitted this list.	Not Applicable	

Data Availability

Data availability	Information included in the manuscript?	In which section is the information available? (Reagents and Tools Table, Materials and Methods, Figures, Data Availability Section)
Have **primary datasets** been deposited according to the journal's guidelines (see 'Data Deposition' section) and the respective accession numbers provided in the Data Availability Section?	Not Applicable	
Were **human clinical and genomic datasets** deposited in a public access-controlled repository in accordance to ethical obligations to the patients and to the applicable consent agreement?	Not Applicable	
Are **computational models** that are central and integral to a study available without restrictions in a machine-readable form? Were the relevant accession numbers or links provided?	Not Applicable	
If publicly available data were reused, provide the respective **data citations in the reference list**.	Not Applicable	
